# Electron Transport in a Dioxygenase-Ferredoxin Complex: Long Range Charge Coupling between the Rieske and Non-Heme Iron Center

**DOI:** 10.1371/journal.pone.0162031

**Published:** 2016-09-22

**Authors:** Wayne K. Dawson, Ryota Jono, Tohru Terada, Kentaro Shimizu

**Affiliations:** 1 Department of Biotechnology, Graduate School of Agricultural and Life Sciences, The University of Tokyo, 1-1-1 Yayoi, Bunkyo-ku, Tokyo, 103–8657, Japan; 2 Laboratory of Bioinformatics and Protein Engineering International Institute of Molecular and Cell Biology in Warsaw, ul Ks. Trojdena 4, 02–109, Warsaw, Poland; 3 Laboratory of Functional and Structural Genomics, Centre of New Technologies, University of Warsaw, Banacha 2C, 02–089, Warsaw, Poland; 4 Research Center for Advanced Science and Technology, The University of Tokyo Komaba, Meguro-ku, Tokyo, 153–8904, Japan; 5 Agricultural Bioinformatics Research Unit, Graduate School of Agricultural and Life Sciences, University of Tokyo, 1-1-1 Yayoi, Bunkyo-ku, Tokyo, 103–8657, Japan; Universidade Nova de Lisboa Instituto de Tecnologia Quimica e Biologica, PORTUGAL

## Abstract

Dioxygenase (dOx) utilizes stereospecific oxidation on aromatic molecules; consequently, dOx has potential applications in bioremediation and stereospecific oxidation synthesis. The reactive components of dOx comprise a Rieske structure Cys_2_[2Fe-2S]His_2_ and a non-heme reactive oxygen center (ROC). Between the Rieske structure and the ROC, a universally conserved Asp residue appears to bridge the two structures forming a Rieske-Asp-ROC triad, where the Asp is known to be essential for electron transfer processes. The Rieske and ROC share hydrogen bonds with Asp through their His ligands; suggesting an ideal network for electron transfer via the carboxyl side chain of Asp. Associated with the dOx is an itinerant charge carrying protein Ferredoxin (Fdx). Depending on the specific cognate, Fdx may also possess either the Rieske structure or a related structure known as 4-Cys-[2Fe-2S] (4-Cys). In this study, we extensively explore, at different levels of theory, the behavior of the individual components (Rieske and ROC) and their interaction together via the Asp using a variety of density function methods, basis sets, and a method known as Generalized Ionic Fragment Approach (GIFA) that permits setting up spin configurations manually. We also report results on the 4-Cys structure for comparison. The individual optimized structures are compared with observed spectroscopic data from the Rieske, 4-Cys and ROC structures (where information is available). The separate pieces are then combined together into a large Rieske-Asp-ROC (donor/bridge/acceptor) complex to estimate the overall coupling between individual components, based on changes to the partial charges. The results suggest that the partial charges are significantly altered when Asp bridges the Rieske and the ROC; hence, long range coupling through hydrogen bonding effects via the intercalated Asp bridge can drastically affect the partial charge distributions compared to the individual isolated structures. The results are consistent with a proton coupled electron transfer mechanism.

## Introduction

Reactive oxygen centers (ROCs) are an important means of metabolizing or oxidizing various polyaromatic hydrocarbons (PAH) [1–5]. An ROC often comprises a mononuclear iron ion bound by two His ligands, one Asp ligand, and one or two water molecules that also function as ligands. The ROC is often coupled to an iron–sulfur center, which is an inorganic electron storage structure that typically is found in the form of a rectangular-shaped [2Fe-2S] complex or a parallelepiped-shaped [4Fe-4S] complex (as seen in some photosynthetic Fdx proteins) [6,7]. This study is directed to oxygenase (Ox) proteins, which contain both a ROC and an iron–sulfur center. Ox is found in the form of a mono-oxygenase (mOx) and a dioxygenase (dOx), depending on whether the oxidized form of the PAH gains one or two oxygens. In many Ox proteins, a Rieske structure serves as the [2Fe-2S] center [8,9]. The Rieske is isolated from the ROC by a dense network of hydrophobic residues requiring transmission either via electron tunneling or proton coupled electron transfer (PCET) [10–15] via a collimated network of hydrogen-bonded conjugated residues or aromatic residues.

**Fig 1:**
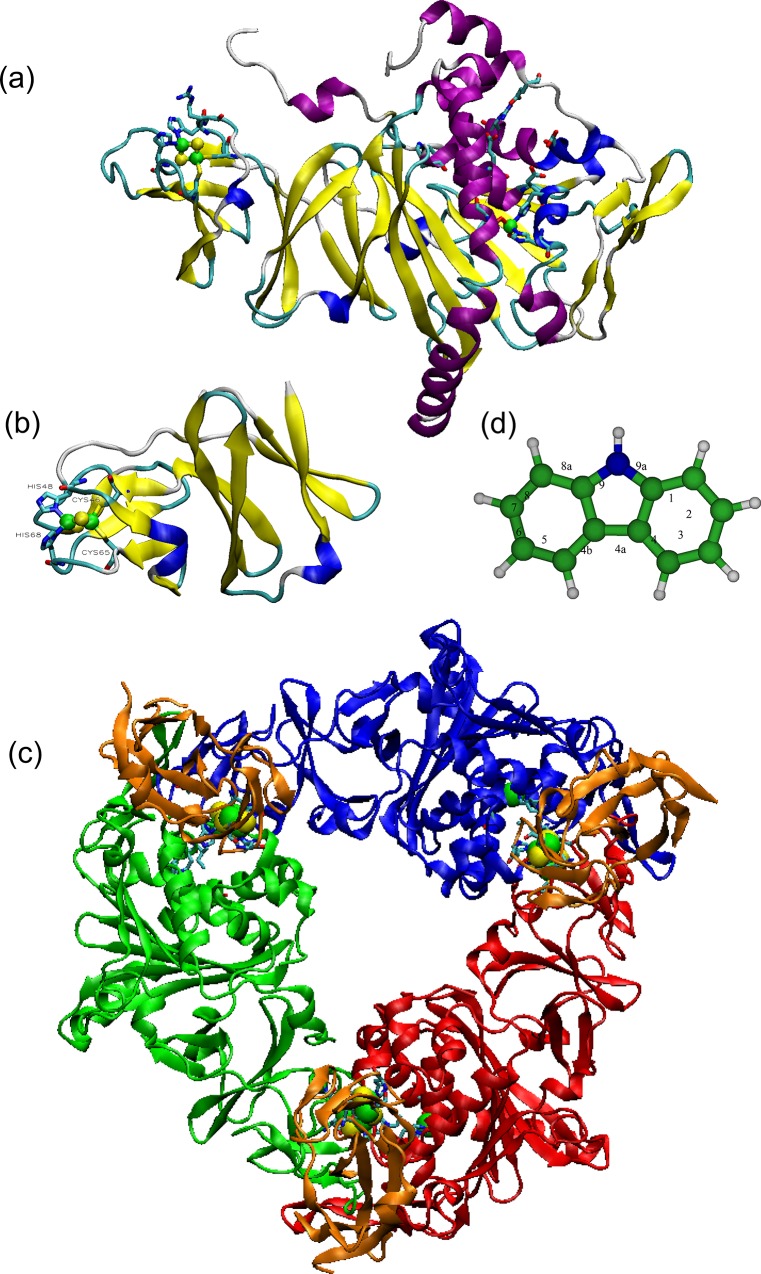
General information about the 1,9a-dioxygenase/ferredoxin complex. (a) The structure of a single subunit of carbazole 1,9a-dioxygenase (CRDo: 1WW9). Amino acids associated with the electron transport network are also shown: (green beads) Fe and (yellow beads) S. (b) The structure of the ferredoxin (CRDf: 1VCK). c) The complete complex of 1,9a-dioxygenase (CRD: 1WW9, red, blue and green) and its partner ferredoxin 1VCK (brown) bound in a trimer complex (2DE5). (d) The structure of carbazole where the oxidation of this structure by CRD occurs at the 1 and 9a positions.

As a complex, where *both* the relatively sedentary dioxygenase (dOx) and the itinerant and elusive ferredoxin (Fdx) molecules are found joined together, only one select group of examples [4] from this family has been successfully crystalized and measured using X-ray crystallography. Specifically, this complex is an X-ray structure of the dOx molecule carbazole 1,9a-dioxygenase (e.g., the oxidized structure PDB: 1WW9, Fig 1A) [16] and its Fdx partner (PDB: 1VCK, Fig 1B) [3] at 1.9 Å resolution (PDB: 2DE5-7, Fig 1C). This class of dOx molecules form crystal in a trimer structure, which is usually abbreviated as α_3_ (Fig 1C). The Fdx binds to the α_3_ complex reproducibly around the boundary region between any two adjoining dOx subunits in the trimer structure (Fig 1C, brown structures). A reduced structure (2DE6) and an oxidized structure containing the carbazole molecule in the redox chamber (2DE7) were also crystallized and measured. In the literature, the components of 2DE5–7 have been labeled CARDO-o for the carbazole 1,9a dOx and CARDO-f for the Fdx cognate [3,16–20]. In this study, these labels are used extensively; consequently, we have abbreviated these labels as CRDo (for CARDO-o) and CRDf (for CARDO-f), respectively.

CRDo oxidizes carbazole at the 1,9a position (Fig 1D). Carbazole is a component of crude oil, a significant byproduct of burning coal at high temperatures, and a byproduct of cigarette smoke [21]. Carbazole is also used in industrial production of dyes, pharmaceuticals, and plastics [21–23]. Bacteria that break down carbazole using dOx pathways have been isolated from marine environments [24,25], possibly reflecting exposure to crude oil. Identifying the mechanism for the breakdown of toxic compounds by these organisms may prove useful in bioremediation [17,21,26–28], synthesis of stereochemical oxidation products, and potentially drug development [28]. Even restricted to bacteria, the process involved in oxidizing PAHs is diverse (Table 1). CRDo is defined as a type-IIIα dOx system [1]. Three components are involved in the ET process. The first component is a reductase, flavin-adenine-dinucleotide (FAD) with a plant-type [2Fe-2S] cluster [29]. The reductase transfers an electron to the second component, which is a small and mobile Fdx with a Rieske-type ligand structure. The reductase can often interact with more than one type of Fdx molecule [29]. The third component is the trimer form of dOx, (dOx)_3_ (Fig 1C, inner region). The Fdx diffuses between the reductase and (dOx)_3_ forming (dOx)_3_:(Fdx)_n_ (n = 0, 1, 2, or 3). When n = 3, as in the case of 2DE5–7, three molecules of Fdx are interacting with the (dOx)_3_ trimer simultaneously: (CRDo)_3_:(CRDf)_n = 3_ (Fig 1C, outer regions, brown structures). In the oxidation process of carbazole, the water molecule(s) that normally bind to the non-heme Fe become replaced with O_2_ [30,31] (Fig B in S1 File). Subsequently, CRDo carries out the first step in the oxidation of the 1 and 9a positions of the carbazole structure (Fig 1D) to produce 2′-aminobiphenyl-2,3-diol. Two additional steps with other specialized dOx proteins eventually break down carbazole such that it can be consumed and excreted through standard biochemical pathways [26,32].

**Table 1:** Examples of known aromatic hydrocarbons frequently encountered in the environment, for which there are known ring hydroxylating dioxgenases and their corresponding ferredoxin partners.

compound	Oxygenase	Ferredoxin	Examples of PDB structures
Carbazole 1,9a	[2Fe-2S]/Fe^2+^	[2Fe-2S]	2DE5/6/7, 1WW9
Phthalate	[2Fe-2S]/Fe^2+^	[3Fe-4S]	2PIA
Benzoate 1,2	[2Fe-2S]/Fe^2+^	None	1KRH
Cumine	[2Fe-2S]/Fe^2+^		1WQL
Dioxin	[2Fe-2S]/Fe^2+^	[2Fe-2S]	
Biphenyl	[2Fe-2S]/Fe^2+^	[2Fe-2S]	2GBX/W
Toluene	[2Fe-2S]/Fe^2+^	[2Fe-2S]	2INC/D,2RDB
Naphthalene	[2Fe-2S]/Fe^2+^	[2Fe-2S]	2HMJ/K/L/M/N/O
Aniline	[2Fe-2S]/Fe^2+^	[2Fe-2S]	

Adapted from Ref [1].

**Fig 2:**
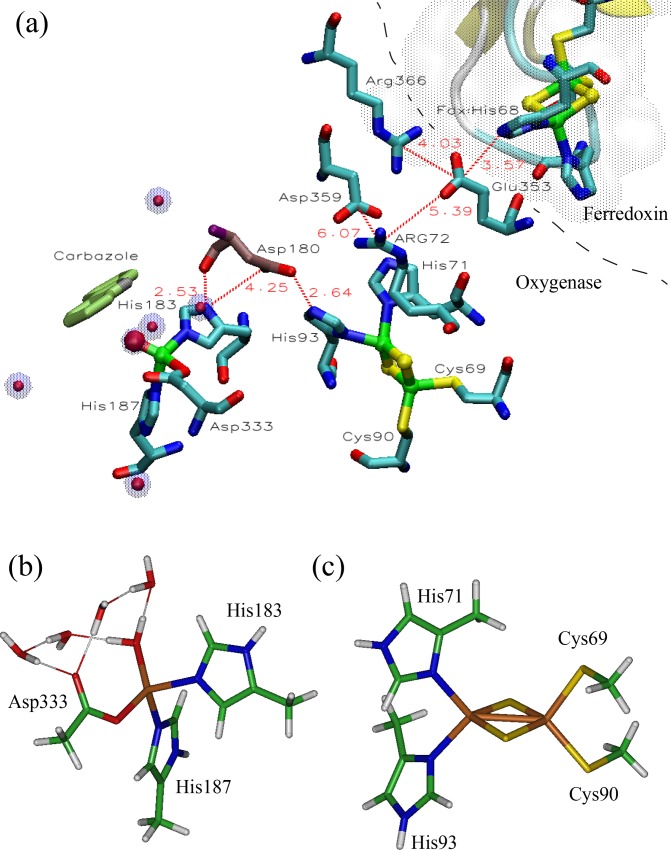
The core reactive domains of the 1,9a-dioxygenase protein/Ferredoxin complex (PDB id: 2DE5). (a) The key components from the trimer complex (Fig 1C). On the left is the non-Heme Iron redox center (ROC: Fe is the green atom) and near the center is the iron-sulfur (Cys)_2_[2Fe-2S](His)_2_ structure (the Rieske domain). Between the Rieske and the ROC is the universally conserved Asp. (b) The core structure of the ROC including the two His residues (methyl imidazole), one Asp residue (acetate), a bound water molecule and four additional optimized water molecules (found in the chamber) configured around the bound water and the Asp residue. (c) The Rieske domain including the two Cys residues (thio methylate) and two His residues (methyl imidazole).

The electron charge transfer and storage in the (dOx)_3_:(Fdx)_3_ complex is facilitated by a Rieske domain in both dOx and Fdx, and the oxidation of the PAH is carried out by the ROC (a non-heme Fe center). For the CRDo/CRDf system, the interaction between the dOx and Fdx is shown in Fig 2A. The ROC (mononuclear Fe) is coordinated with two His ligands (bound at the Nε position), one Asp ligand, and in some cases one water ligand or in others two water molecules [1,33–35]: see Section 2 and Fig B in S1 File for examples of various observed configurations around the non-heme iron. In addition to bound water, in the 2DE5-7 structures, several water molecules tend to coordinate around the bound water and the Asp ligands (Fig 2B). The Rieske domain is an iron–sulfur structure [2Fe-2S] bound by four ligands, two cysteine side chains forming Fe–Sγ bonds, and two histidine side chains forming Fe–Nδ bonds [8,9]. The organization of the residues takes the form (Cys)_2_[2Fe-2S](His)_2_ (Fig 2C). The sequence homologies of Fdx and dOx in the Rieske domains have little resemblance except for the CxH…CxxH pattern.

**Fig 3:**
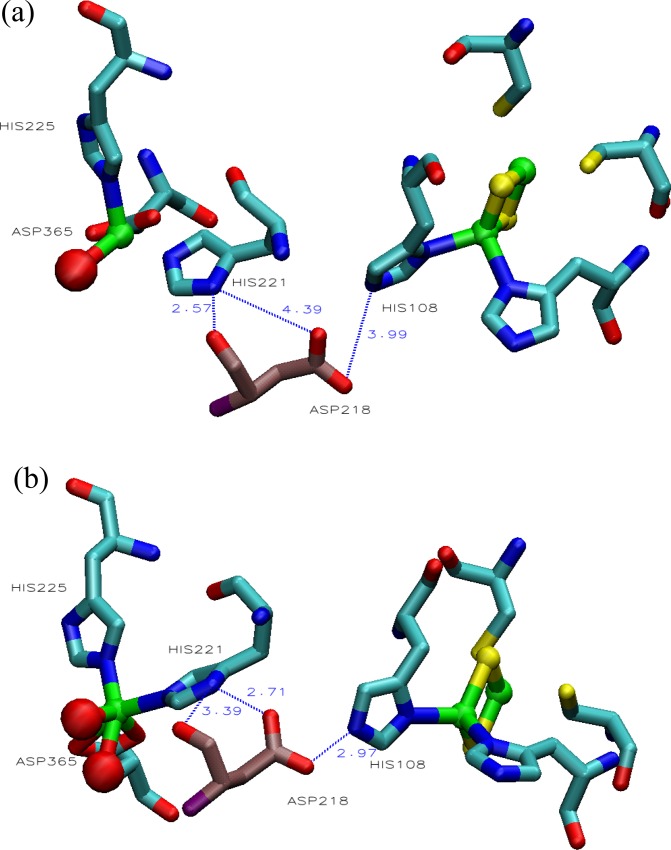
**Relationship between the universally conserved Asp, the Rieske and the reactive oxygen center (ROC) in the oxidized state (a: 1Z01) and the reduced state (b: 1Z02).** In (a), the distance between Asp218 and His221 is comparatively far, whereas in (b), the distance between Asp218 is nearly the same between His108 and His221.

Linking the [2Fe-2S] and the ROC in the dOx is a universally conserved Asp residue (Fig 2A, Asp180, near the center, red tinted residue). In the oxidized state, the carboxyl side chain of the Asp does not tend to interact with both His interfaces (Fig 3A); however, in the reduced state, this triad aligns such that the Rieske His(Hε) and the ROC His(Hδ) interact through the carboxyl [36] (Fig 3B): (ROC)His-Hε…O-C-O…Hδ-His[2Fe-2S]. This conserved Asp residue has also been shown to be essential based on mutation studies of naphthalene dOx [37], phthalate dOx [38], and toluene dOx [39].

From Mossbauer experiments, it is known that the unpaired electron tends to localize on the His side of the Rieske structure [40,41]. In the high temperature regime (*T* > 40 K), the relaxation rate gradually is dominated by the Orbach relaxation process [42–44]. In this regime, the exchange coupling |*J*| of the (Cys)_2_[2Fe-2S](Cys)_2_ structure (4-Cys) is roughly twice the Rieske structure [43,45,46]. Part of this appears to be due to a more observable antisymmetric exchange in the [2Fe-2S]^1+^ cluster [47]: characterized by the higher upward shift of the g-value in many Rieske structures. However, it has also been shown that the exchange coupling of the Rieske can be shifted toward the 4-Cys values by eliminating specific hydrogen bonds (H-bonds) between the protein and the sulfur atom in the bridge [44].

In this study, we examine changes in the partial charges of the universally conserved Rieske-Asp-ROC complex found in all α_3_ type Ox proteins, where the parts are first evaluated separately and then coupled via the conserved Asp (Asp180 in the case of this dOx, PDB ids 1WW9 and 2DE5-7). In this study, we found that the charges and energies within the Rieske and the ROC are affected by the interposition of the Asp180-bridge and that this coupling can be long range between the different ligands and amino acid side chains. Using quantum chemistry/quantum mechanics (QM) techniques, we characterize the normal modes of the ROC, Rieske (and 4-Cys) parts by benchmarking the calculations with known experimental data (when available) for the oxidized and reduced cases and we evaluate the full ROC-Asp-Rieske complex. We also benchmark the partial charges found in this study with the well-established Amber force field. Finally, we construct a set of force field parameters for carrying out simulations on these complexes, which will be used in future studies.

## Results and Discussion

The primary aims of this study are to examine the coupling between the Rieske and the ROC parts via the Asp bridge and to establish reasonable correspondence between different methods such as quantum chemistry and molecular mechanics force fields that are supported with the available experimental data for the individual components of the complex. In as much as possible, the results should largely agree between different choices of density functional theory, the experimental data and the established force field parameters. In achieving this state of affairs, it is hoped that the reliability and utility of the reported results is improved. Therefore, in the sections that follow, we often go rather deeply into comparing the findings here with whatever known experimental data and force field information can be found.

### Evaluating and benchmarking the partial charges

As a benchmark for this study, we compare the partial charges obtained on the ROC-Asp-Rieske complex and the individual components with the partial charges commonly used in Amber ff99SB protein force field for the isolated amino acid residues. Although there is room for debate on what to use, the partial charges for the various amino acids in ff99SB represent a well-tuned and tested standard; hence, benchmarking to this standard is, at the very least, informative.

To determine the partial charges, the developers of the Amber force field recommend the use of Hartree–Fock (HF) methods to obtain the partial charges [48]; however, optimizations of the (MeS)_2_[2Fe-2S](SMe)_2_ complex at the HF/6-311+G(d) level yielded an exceedingly distorted a structure. The Fe–Fe bond lengths were nearly twice the observed bond-length and S–Fe–S bond angles were greatly distorted. Such significant deviations discouraged the use of HF in these calculations.

Density functional (DF) methods proved far better. For the [2Fe-2S] structure, we began the study at the recommended BP86/6-311+G(d) level and arranged the AF spin structure using Generalized Ionic Fragment Approach (GIFA) [6]. For the **D**_2_ symmetry structure of (MeS)_2_[2Fe-2S](SMe)_2_, optimizing at the B3LYP/6-311+G(d) level yielded an Fe–Fe bond length of 2.83 Å, BP86/6-311+G(d) 2.68 Å and OPBE 2.71 Å (Table 2) [6,49,50]. The experimentally observed length for the oxidized state is 2.71–6 Å [6,49]. The B3LYP result is much larger than the BP86 and OPBE results, consistent with previous reports [6].

**Table 2:** Bond lengths dependence on different density functional models for the structure (MeS)_2_[2Fe-2S](SMe)_2_.

State	Source (sym)	Fe–Fe	Fe–S^b^	S^b^–S^b^	Fe–S^t^	ref
Oxidized	BP86(**D**_2h_)	2.77	2.23	3.50	2.36	
	B3LYP(**D**_2h_)	2.93	2.28	3.49	2.38	
	OPBE(**D**_2h_)	2.79	2.23	3.48	2.36	
	BP86(**D**_2_)	2.68	2.21	3.51	2.35	
	B3LYP(**D**_2_)	2.83	2.26	3.53	2.37	
	OPBE(**D**_2_)	2.71	2.21	3.49	2.35	
	X-ray: Pdx	2.71/2.72	2.22	3.55	2.35	Obs (a)
	X-ray: S-o-xyl	2.698	2.209	3.498	2.305	Obs (b)
	X-ray Plant ferredoxin	2.72	2.01	3.54	2.23	Obs (b)
Reduced	BP86(**D**_2h_)	2.78	2.25	3.53	2.39	
	B3LYP(**D**_2h_)	3.01	2.26/2.39	3.55	2.48	
	OPBE(**D**_2h_)	2.83	2.26	3.51	2.41	
	BP86(**D**_2_)	2.75	2.21/2.27	3.54	2.43, 2.21/	
					2.27, 2.41	
	B3LYP(**D**_2_)	2.95	2.24/2.40	3.58	2.47/2.49	
	OPBE(**D**_2_)	2.78	2.20/2.90	3.52	2.44/2.40	
	X-ray: Pdx	2.69/2.74	2.23	3.53	2.30	Obs (a)

The oxidized charge state [(Fe^3+^, *s* = 5/2):(Fe^3+^, *s* = −5/2)] with net multiplicity *S* = 1, where *s* is the spin on the particular Fe ion, (reduced) charge state [(Fe^3+^, *s* = 5/2): (Fe^2+^, *s* = −4/2)] with net multiplicity *S* = 2. Calculations of the spin state were carried out using Gaussian 09 combined with the GIFA method [6]. (a) Ref [49], (Pdx) Putidaredoxin with some mutations at non-ligand binding residues (b) Ref [50], (S-o-xyl) ([2Fe-2S](o-xylylenedithiolate)_2_ structure.

Based on these results, calculations at both the BP86/6-311+G(d) and OPBE/6-311+G(d) levels were used because both yielded reasonable agreement with the observed experimental geometry and computational economy. BP86 was a recommended density functional for these problems [6,51]. For the largest complexes, calculations were carried out using OPBE/cc-pVTZ exclusively due to improved stability.

Further extensive testing of density functions and bases sets showed that optimizing at the OPBE/cc-pVTZ level yielded the best results because of the far better balance in the Dunning triple zeta basis set. In this work, the results are all reported using this level of theory unless otherwise specified. Therefore, the final reported results are from calculations at the OPBE/cc-pVTZ level unless otherwise specified. Final geometry optimizations were done with g03 at the OPBE/cc-pVTZ level for the largest complexes in this work with a final SCF calculation carried out using g09. The final Rieske, 4-Cys, and ROC structures use g09 exclusively at the OPBE/cc-pVTZ level, though these were also calculated independently using g03.

In the optimized Rieske, ROC, and complete complex, partial charges were analyzed using RESP in the Amber 10 package [52] to integrate these substructures into the Amber ff99SB force field. For both the ROC and [2Fe-2S], the individual Antechamber programs had to be run separately (manually) to build the force field files. Antichamber is not designed to assign “BOND” relationships between free molecules that associate with the principal structure of interest, particularly in the case of locally coordinating water molecules. Therefore, it was necessary to assign the BOND relationship in such a way as to link the chain of free water molecules through the oxygen. Improper selection of these “BOND” relationships resulted in unrealistic partial charges that did not correspond to any established water model, let alone the TIP3P water used in these simulations.

The CHelpG (CHarges from Electrostatic Potentials using a Grid) based method [53,54] tended to generate partial charges similar to RESP1 without the additional subjective inclusion of bond definitions. In this respect, the CHelpG approach is more objective. Nevertheless, both methods generally produced comparable partial charges on the main structure and the independent water molecules (required in some of these calculations) when a proper bond selection was determined. Calculations results are benchmarked with existing Amber 94 partial charges when available.

In general, since these parameters were carefully determined through an extensive optimization fitting approach [48,55], the partial charges for structures under similar conditions should reflect similar partial charges in the predictions using CHelpG or RESP. Nevertheless, some RESP results underestimated partial charges on the Fe, particularly for the reduced state of the Rieske structure and some states of the ROC. Therefore, although partial charges were often compared with their RESP equivalents, those reported here are based on CHelpG results.

Details of using g03 and g09 to evaluate the electronic states with GIFA are explained in Section 3 in S1 File. Coordinates for building fragments using GIFA are shown in Listing 1 in S1 File, the mergelist files for building different spin states of the complex are found in Listing 2 in S1 File, and coordinates of the oxidize and reduced complex can be found at Listings 3 and 4 in S1 File, respectively.

### Cluster calculations

**Fig 4:**
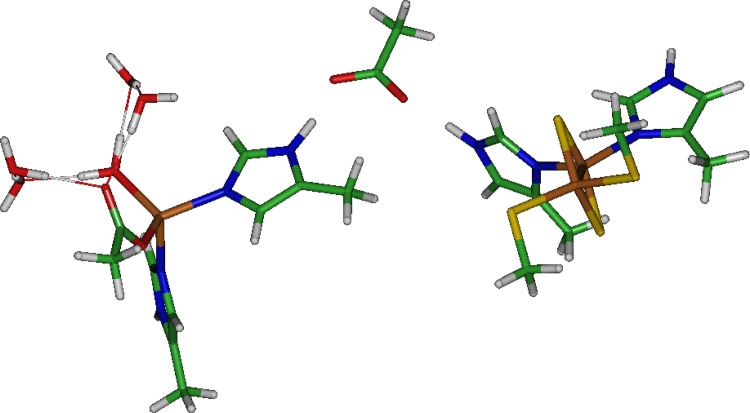
Results of optimization of the Rieske–Asp–non-heme Fe reactive oxygen center (ROC) complex computed with a minimum of two distance constraints: one between the Rieske:Fe (on the His side) and the ROC:Fe, and the other between H183:Cβ and H93:Cδ2. The orientation of the Rieske and ROC and their separation distances reflect what was found in the crystal structures of these compounds.

We sought to examine the long range coupling in the proposed electron transport (ET) network using quantum chemistry and to develop force field parameters for the CRDo and CRDf structures to carry out further verification using MD simulations. To build a proper force field, the quantum chemistry should examine the entire network of bonds, including the array of conjugated bonds between the Fdx interface, the dOx Rieske, and the ROC. At present, building and optimizing the full network structure is an extremely expensive procedure and is certainly beyond available laboratory resources. However, the known interactions between His93, Asp180, and His183 can be constructed using simplified molecules and a minimal number of optimization constraints (Fig 4).

#### 4-Cys-[2Fe-2S] structure

**Fig 5:**
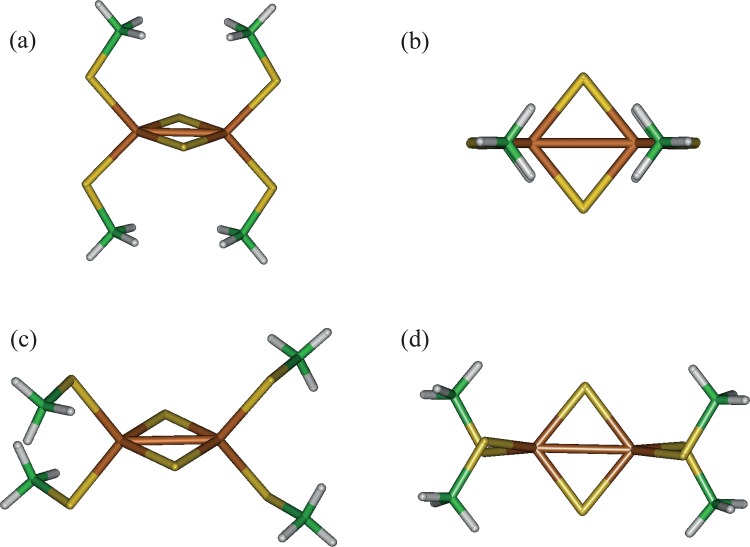
High symmetry structures of the 4-Cys-[2Fe-2S] complex, specifically (MeS)_2_[2Fe-2S](SMe)_2_. structures (a) and (b) with **D**_2h_ symmetry and structures (c) and (d) with **D**_2_ symmetry.

The first step was to determine a level sufficient to ensure that the applied theory could obtain partial charges and force field parameters. To understand the performance, we needed to compare calculations with known experimental data whenever possible. Most of the experimentally obtained spectroscopic data for the [2Fe-2S] structure has been obtained from the 4-Cys: a structure of the form (Cys)_2_[2Fe-2S](Cys)_2_. Therefore, we began by using the 4-Cys system as a benchmarking model. The Cys ligand was simplified to a thiomethyl anion (SMe^−^) resulting in the structure (SMe)_2_[2Fe-2S](SMe)_2_: one structure with **D**_2h_ (Fig 5A and 5B) and the other with **D**_2_ symmetry (Fig 5C and 5D). The Fe ions in the [2Fe-2S] ring exhibit antiferromagnetic (AF) coupling between the electron spins in the ring. To address this using g09 (or g03), the GIFA method [6] was used to feed an initial ionic spin and charge state for the Fe, S and the ligands into the checkpoint file for further g09 calculations.

As a footnote, for the high symmetry structures (in the reduced state) shown in Fig 5, the combination of DFT with the 6–311+G(d) basis sets tended to result in spin contamination in the spin density matrix for B3LYP, BP86, and OPBE. However, the TZVP and cc-pVTZ basis sets generate stable spin density matrices. Hence, although the actual results of bond distances and the normal modes are similar at the OPBE/6-311+G(d) level even with this spin contamination, the results for these structures are all reported based on calculations using OPBE/cc-pVTZ. These problems largely stem from the high-symmetry of the structures (Fig 5). For example, when structures from the PDB are used, they typically lack this level of symmetry and exhibit no significant spin contamination (largely independent of DF and basis set).

#### 4-Cys Bond lengths

Experimentally measured bond lengths for the 4-Cys-[2Fe-2S] structures are listed in Table 2 for the oxidized state [49,50,56–58] and reduced state [59]. From the optimized structural calculations, the bond lengths are determined to be symmetry dependent with the **D**_2h_ structure (Fig 5A and 5B), showing a longer Fe–Fe bond length than the **D**_2_ structure (Fig 5C and 5D). For both the oxidized state and reduced state, the bonds are consistently longer for the **D**_2h_ structures using the same level of DFT. The reduced state consistently shows longer bond lengths for Fe–Fe and Fe–S(b) compared to the oxidized state in the experimental data. This probably reflects the increased electron–electron repulsion generated by the extra charge in the [2Fe-2S] ring. When the predictions are compared with the experimental results (Table 2), it is evident that BP86 and OPBE clearly yield the best results.

#### 4-Cys Normal modes

Table 3 compares the observed normal modes [50,57–62] with the calculated values. An expanded version of Table 3 is also available in S2 File. The AF spin polarized Fe^3+^ ion structures evaluated at the OPBE/6-311+G(d) level show qualitative agreement with the observed normal modes (scaled by 1.06 and 1.1 for the oxidized and reduced states, respectively). In the oxidized state, the DF model underestimates the observed values; a factor of approximately 1.06 is required to achieve a reasonable fit. The reduced state in the experimental data is down-shifted by approximately 10% from the oxidized state. Calculated frequencies are also down-shifted proportionally (Table 3). Indicative of this downshift in frequency in the reduced state is a corresponding extension of the bond length, particularly the Fe–Fe distance (2.71–2.72 Å) and the Fe–S(b) bond (2.21–2.26 Å); i.e., the shorter the Fe–Fe distance, the higher the frequency. This can be seen in the raw data of the uncorrected AF spin coupling where the Fe–Fe bond length reduces to 2.59 Å (see the Tables in S2 File).

**Table 3:** Observed and calculated frequencies of normal modes for the 4-Cys-[2Fe-2S] complex.

State	Oxidized				State	Reduced		
	Observed			Calculated		Observed		Calculated
	Adr	Pdx	SMet	Scaled		Adr	Pdx	Scaled
Normal	ref (a)	ref (a)	ref (b)		Normal	ref (a)	ref (a)	
Mode	[cm^−1^]	[cm^−1^]	[cm^−1^]	[cm^−1^]	Mode	[cm^−1^]	[cm^−1^]	[cm^−1^]
B_2u_^b^	421	426	409	427.6	B_1_^b^	398.0	406.0	406.3
A_g_^b^	393	400	385	390.3	A_1_^b^	377.0	381.0	383.2
B_3u_^b^	349	350	345	342.5	A_1_^b^	307.0	307.0	311.1
B_1u_^t^	341	344	329	329.2	B_2_^t^		319.0	268.1
B_2g_^t^	341	344		308.1	B_2_^t^			251.7
A_g_^t^	329	338	322	304.6	A_1_^t^	307.0	307.0	262.0
B_1g_^b^	317	320	313	298.8	B_1_^b^	276.0	273.0	284.9
B_3u_^t^	291	291	279	261.0	A_1_^t^	258.0	273.0	237.1
A_g_^Fe-Fe^			210	208.7	A_1_			109.6

More complete data are provided in the Tables in S2 File. Scaling factors are 1.06 and 1.1 for the oxidized and reduced states, respectively. (a) Ref [59]: (Adr) bovine adrenodoxin and (Pdx) putidaredoxin (b) Ref [60]: (SMet) the structure (MeS)_2_[2Fe-2S](SMe)_2_

The correspondence between the observed and predicted normal mode frequencies is approximate, and there are also differences in the assignment symmetry. Some of the frequency assignments are strongly dependent on the configuration of the ligands in the structure where a minor rearrangement of the ligands can easily shift the modes in the range of 330–360 cm^−1^. Although several configurations were verified, the most critical modes needed to determine the force constants for bond stretching (Fe–Fe and Fe–S(b)) and the related bending modes (S(b)–Fe–S(b), S(t)–Fe-S(b) and S(t)–Fe–S(t)) can be deduced from data of the normal modes.

Table 4 shows the extrapolated bond stretching and bending constants for the oxidized state [57,60]. From previous studies, we realize that there is considerable variation in the experimental estimates obtained at different times by the same group [57,60]. This indicates the difficulty of determining a precise fit for these force constants. Because of the vast complexity of the interactions, it is expected that the parameters will have a wide range of values. For example, the [2Fe-2S] structure has out-of-plane bending, breathing modes, symmetric stretch, and antisymmetric stretch. Hence, these modes can only be roughly approximated using the simple stretch, angle, and dihedral angle parameters used in MD simulation approaches.

**Table 4:** Reported stretching and bending constants for 4-Cys-[2Fe-2S] in the oxidized state based on observed data.

	Force constant					
Mode	ref (a)		ref (b)		calculated	
	[mdyne/Å]	[kcal/molÅ^2^]	[mdyne/Å]	[kcal/molÅ^2^]	[mdyne/Å]	[kcal/molÅ^2^]
K(Fe–S(b))	1.37	197	1.44	207	1.450	209
K(Fe–S(t))	1.14	164	1.13	163	1.145	165
K(S–C)	2.50	360	2.00	288		
K(C–C)	4.80	691	2.50	360		
H(Fe–S(b)–Fe)	0.45	65	0.27	39	0.627	90.
H(S(b)–Fe–S(b))	0.40	58	0.25	36	0.634	91.
H(S(b)–Fe–S(t))	0.38	55	0.25	36	0.488	70.
H(S(t)–Fe–S(t))	0.35	50	0.20	29	0.488	70.
H(Fe–S–C)	0.35	50	0.15	22		
H(S–C–C)	0.82	118	0.15	22		
K(Fe–Fe)	0.19	27	0.27	39	0.230	33

(a) Experimental data from Ref [60] and (b) from Ref [57]. The calculated data is based on estimates from the computed normal modes in this work. Finite difference calculations of Fe–Fe and Fe–S(b) yielded values within 20% of those estimated from the normal mode. The notation K and H indicate stretching and bending, respectively.

The last column of Table 4 (kcal/molÅ^2^) shows estimates of the force constants based on the calculated normal modes. Some of these were also tested with finite difference techniques.

Referring to Table 3 (column SMet), the normal mode at 210 cm^−1^ (sym: A) is strongly associated with Fe–Fe stretching. The estimated spring constant is 0.22 mdyne/Å (32 kcal/mol·Å^2^), which is close to the experimental estimates (Table 4). The mode at 313 cm^−1^ (sym: B) is strongly associated with Fe–Sγ stretching. The estimated spring constant is 1.01 mdyne/Å (145 kcal/mol·Å^2^), which is also similar to the experimental estimate. The mode at 329 cm^−1^ (sym: B) is strongly associated with Fe−S(b) stretching. The estimated spring constant is 2.03 mdyne/Å (292 kcal/mol·Å^2^), which is overestimated. Nevertheless, the trend qualitatively agrees with the experimental estimates (Table 4) obtained from (Cys)_2_[2Fe-2S](Cys)_2_ measurements of these spectra [50,56–58]. The S(b)–Fe–S(b) bending and Sγ–Fe–Sγ bending should be of similar order; therefore, these were grouped together. Finally, bending modes are determined in the computed spectrum: 137 cm^−1^ (sym: B) Sγ−Fe−S(b). The predicted force constants (Table 4) are general estimates; however, they are within a factor of two of the values used in Refs [57,60]. Moreover, similar variation is also observed in the data provided in Refs [57,60]. These values were also estimated directly using finite difference techniques (Section 4.3.1 in S1 File).

#### Rieske structure

To test the Rieske structure, methyl imidazole and thiomethyl fragments were constructed and attached to the [2Fe-2S] ring (Fig 2C).

**Fig 6:**
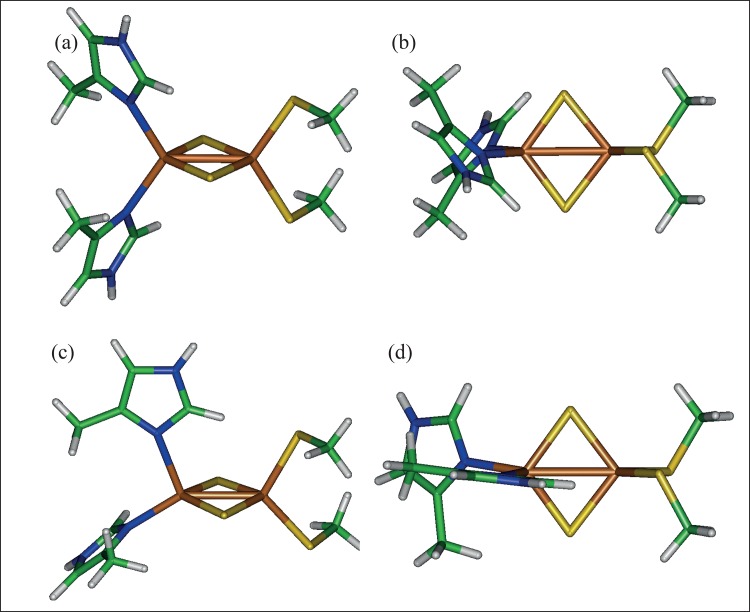
High symmetry structures of the Rieske complex, specifically (Imidazole)_2_[2Fe-2S](SMe)_2_. (a and b) oxidized structure with maximum **C**_2v_ symmetry and (c and d) the reduced structure with **C**_1_ symmetry.

*Rieske bond lengths*. Table 5 shows the bond lengths (Fe–S(b), Fe–Fe, Fe–Sγ, and Fe–Nδ) obtained at the OPBE/cc-pVTZ level along with precise measurements of the bond lengths obtained from x-ray spectroscopy on small molecules. Similar results were also found in Ref [63]. In the Tables in S2 File, the Table containing “bond lengths” shows the average [2Fe-2S] bond lengths for a large collection of PDB structures containing the Rieske structure. The averaged bond lengths and those predicted for the oxidized and reduced states are of similar order to those presented in Table 2. In the calculated results, as in the case of 4-Cys-[2Fe-2S], the elongation of the Fe–Fe and Fe–S(b) bonds in the reduced state is observed: consistent with the possible increase in electron–electron repulsion within the [2Fe-2S] ring in the reduced state. The structure with maximum symmetry in the oxidized state is shown in Fig 6A and 6B. The reduced structure was initially configured in the form shown in Fig 6A and 6B; however, after optimization, the structure was reconfigured to that shown in Fig 6C and 6D. The reconfigured structure also bears the symmetry of the actual structure within the dOx, suggesting that the configuration within the protein is oriented to optimize the reduced state.

**Table 5:** List of observed and calculated bond lengths for the Rieske structures in the oxidized and reduced state of the structure.

State	Source	Fe–Fe	Fe–S^(b)*^(Cys)	Fe–S^(b)*^(His)	S^(b)^–S^(b)^	Fe–S^(t)*^	Fe–N^(t)*^	Ref
Oxidized	BP86	2.59	2.19	2.17	3.50	2.25/2.24	2.10	
	B3LYP	2.59	2.19	2.17	3.50	2.24	2.10	
	OPBE	2.64	2.22/1	2.22	3.48	2.27	2.13	
	X-ray	2.68	2.20	2.20		2.32	2.05	Obs (a)
	X-ray	2.72	2.26	2.26		2.34	2.09	Obs (b)
	X-ray	2.69	2.23	2.23		2.30	2.08	Obs (b)
Reduced	BP86	2.60	2.23	2.18	3.56	2.33/2.32	2.11/2.10	
	B3LYP	2.72	2.30	2.26	3.65	2.35	2.16/2.22	
	OPBE	2.63	2.23	2.19/2.20	3.55	2.31/2.36	2.07/2.27	
	EXAFS	2.68	(2.25)	(2.25)		2.31	2.09	Obs (a)
	X-ray	2.71	(2.24)	(2.24)		2.26	2.15	Obs (a)
	X-ray	2.72	(2.32)	(2.32)		2.28	2.21	Obs (a)
	X-ray	2.67	(2.20)	(2.20)		2.32	2.05	Obs (c)

(a) Review paper by T.A. Link, Ref [9] (see Table VII, *op*. *cit*.). (b) Hunsicker-Wang et al. Ref [12] (see Table 3 and Fig 2, *op*. *cit*.). Ref [12] is primarily about H-bonding with the 4-Cys structure; however, precise measurements are provided by Ref [88]. Albers et al. report on a chelate rather than a true Rieske; nevertheless, Ref [88] provides some bond lengths that are similar to Rieske bond lengths with the related structure. (c) Tsang et al. Ref [129] They measured the Rieske structure for both reduced and oxidized states. Bond length measurements were reported for Fe–Fe, Fe–S, and Fe–N; distinction between Fe–S^(b)^ and Fe–S^(t)^ was not specified.

* Unless specified as len/len, lengths reflect the average of the two similar bonds.

*Rieske normal modes and comparison with 4-Cys*. Values for the experimental and calculated normal modes are listed in Table 6. For the (His)_2_[2Fe-2S](Cys)_2_ structure, in the normal mode analysis of g09 calculations using Molden 4.8, the major bending and stretching energies were distinguished (Table 6) and compared with the values measured experimentally for the oxidized state [64,65] and the reduced state [65]. The stretching modes in the Fe–S ring (the bridge sulfurs (S^(b)^) bonds) appear between 280 and 430 cm^−1^: an antisymmetric stretch in the [2Fe-2S] plane perpendicular to the Fe–Fe bond axis at approximately 433 cm^−1^ (B_1_^(b)^); a [2Fe-2S] breathing mode symmetric stretch at approximately 410 cm^−1^ (A_1_^(b)^); and an antisymmetric stretch along the Fe–Fe bond axis at approximately 385 cm^−1^ (A_1_^(b)^). The g09 calculations of the normal modes in the oxidized structure clearly distinguish these frequencies quite accurately (cf., Table 6, “observed” and “calculated” columns) [64–66]. Scaling factors were 1.03 and 1.01 for the oxidized and reduced structures, respectively. The remaining assignments marginally agree with the observed signals. The symmetry of the Rieske structure in the oxidized state reduced to only one possible **C**_2_ symmetry axis bisecting the [2Fe-2S] plane and running horizontally (Fig 6A and 6B). Therefore, modes associated with Fe–Sγ stretching will tend to split into Fe–Sγ modes and Fe–Nδ contributions, which are definitely observed in the lower symmetry of the frequency spectrum of normal modes.

**Table 6:** Observed and calculated frequencies of normal modes for the Rieske structure in the oxidized and reduced states.

State	Oxidized			Reduced		
	observed		calculated	Observed		calculated
	Pdx	Ana	scaled	Pdx	Ana	scaled
normal mode	Ref (a)	Ref (b)	scaled	Ref (a)	Ref (b)	scaled
	[cm^−1^]	[cm^−1^]	[cm^−1^]	[cm^−1^]	[cm^−1^]	[cm^−1^]
B_1_ [B_u_^b^]	426.0	433.0	432.7	406.0	408.0	415.6
A_1_ [A_g_^b^]	400.0	410.0	407.6	381.0	377.0	387.2
	-	385.0	407.6	-		
His wobble (in-plane)	-	362.0	373.7	-	[362]	371.6
His wobble (in-plane)	-	362.0	373.6	-	[362]	362.9
A_1_ [A_u_^t^]	344.0	350.0	357.3	319.0	315.0	322.1
B_1_ [B_u_^b^]	350.0	332.0	344.6	307.0	[305]	309.2
B_1_ [B_g_^b^]	320.0	321.0	325.1	273.0	297.0	292.2
B_1_ [B_u_^b^]	-	265.0	290.0	-	246.0	278.9
His wobble (out-of-plane)	-		273.0	-		269.7
A_1_ [A_u_^t^]	-		246.1	-		230.3
A_g_^Fe-Fe^	200.0		219.4	-		192.9

**Fig 7:**
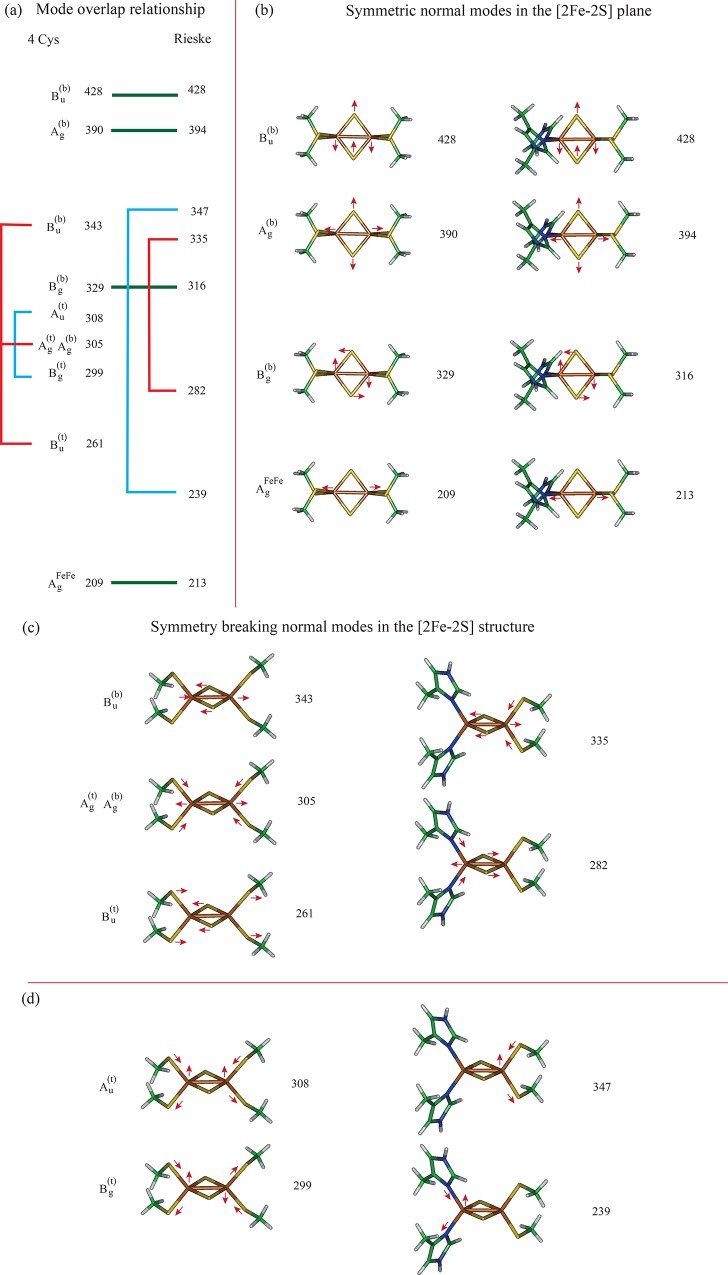
Break down of normal modes for the 4-Cys-[2Fe-2S] and the Rieske structure used in this study. The 4-Cys-[2Fe-2S] is shown on the left along with its calculated frequency and the Rieske structure on the right along with its calculated frequency.

(a) Observed normal modes for putidaredoxin (a 4-Cys ferredoxin) from Ref [59]; (Pdx) putidaredoxin. (b) Experimental data for normal modes in the reduced Rieske structure are from Ref [65]: (Ana) Anabaena 7120 ferredoxin (bacterial oxygenase based protein). The details of mode assignments, based on the 4-Cys structures, can be found in Fig 7. The “[]” indicates the normal mode assignment for the 4-Cys structure.

Comparison of the normal modes of the oxidized state for the (Cys)_2_[2Fe-2S](Cys)_2_ structure and (His)_2_[2Fe-2S](Cys)_2_ are shown in Fig 7. In Fig 7A and listed in Table 6, the vibrational frequencies are shown with the D_2_ symmetry assignment for the 4-Cys-[2Fe-2S] on the left and its relationship to the normal modes of the high symmetry (His)_2_[2Fe-2S](Cys)_2_ structure (Fig 6A and 6B). The green lines connecting the 4-Cys-[2Fe-2S] and Rieske indicate normal modes that are common to both structures involving the D_2_ symmetry of the [2Fe-2S] plane. The corresponding modes of these two structures are compared in Fig 7B. Because half of the Rieske structure is replaced with the His residues, the remaining modes are split and redistributed because of symmetry breaking. The red lines in Fig 7A indicate normal modes where at least one of the Fe ions is moving parallel to the Fe–Fe bond axis and the corresponding modes are compared in Fig 7C. The blue lines in Fig 7A indicate normal modes in which at least one of the Fe ions is moving perpendicular to the [2Fe-2S] plane and the corresponding modes are compared in Fig 7D.

The spectrum range between 200 and 400 cm^−1^ also contains fluctuations between the Cβ and the relatively heavy imidazole (His) ring: approximately 265 cm^−1^ (out-of-plane wobble) and 362 cm^−1^ (in-plane wobble) (Table 6). The 362 cm^−1^ signal can be observed in a number of the spectra listed in Table 6 and in S2 File. At higher wave numbers, the following are seen: Cβ-Sγ twisting (~600–650 cm^−1^), imidazole-Fe and Cβ-Sγ-Fe bending (between 600 and 900 cm^−1^),–CH_3_,–CH_2_, and–CH bending (~900−1200 cm^-1^), C–N and S–CH_3_ asymmetric stretching (~1200 cm^−1^), complex H bending (~1400 cm^−1^), and C–C stretching (~1570 cm^−1^). Above 1570 cm^−1^, the remaining modes all involve H stretching.

In the oxidized state, both the Rieske structure and the 4-Cys show a strong correlation between the respective normal modes for the bridge bonds (Table 6, the [2Fe-2S] modes labeled b). Hence we can use the same procedure to obtain the force constants for these modes [67]. The primary difference is the introduction of vibrational modes from Fe–Nδ stretching and S(b)–Fe–Nδ bending modes. Estimated force constants are shown in Table 7 for the oxidized structures and compared with those presented in Table 4. The A_g_^FeFe^ normal mode prediction for the 4-Cys (Fig 7) is similar to the estimates in Ref [50]. For the Rieske structure, the A_g_^FeFe^ normal mode prediction is almost double the estimates in Ref [50]. The Fe–Fe bond distance of the Rieske is shorter (cf., Tables 2 and 5), suggesting that there is less repulsion in the reduced state of the Rieske structure. This is consistent with the lower electron repulsion due to the His ligands (where the His residues have a neutral formal charge whereas the Cys residues have a formal charge of minus one).

**Table 7:** A comparison of the estimated stretching and bending constants for the oxidized state of the 4-Cys-[2Fe-2S] and Rieske structures, based on calculated normal modes.

	Force constant (oxidized)			
	4-Cys-[2Fe-2S]		Rieske	
Mode	[mdyne/Å]	[kcal/molÅ^2^]	[mdyne/Å]	[kcal/molÅ^2^]
K(Fe-S(b))	1.450	209.	1.095	158.
K(Fe-S(t))	1.145	165.	1.135	163.
K(Fe-N(t))	-	-	0.437	63.
H(Fe-S(b)-Fe)	0.627	90.	0.701	101.
H(S(b)-Fe-S(b))	0.634	91.	0.701	101.
H(S(b)-Fe-S(t))	0.488	70.	0.965	139.
H(S(t)-Fe-S(t))	0.488	70.	0.885	127.
H(S(b)-Fe-N(t))	-	-	0.437	63.
H(N(t)-Fe-N(t))	-	-	0.250	36.
K(Fe-Fe)	0.230	33.	0.378	54.

The notation K and H indicate stretching and bending, respectively.

Table 8 shows the related force field parameters in the reduced structure. In general, the additional electron tends to expand the size of the [2Fe-2S] ring lowering the effective spring constant in the process. Additionally, further splitting of the Fe–S(b) is observed, due to localization of the additional negative charge on the His side of the structure. In the 4-Cys, this effect appears to be less detectable in the charge symmetry of the structure.

**Table 8:** A comparison of the estimated stretching and bending constants for the reduced state of the 4-Cys-[2Fe-2S] and Rieske structures, based on calculated normal modes.

	Force constant (reduced)			
	4-Cys-[2Fe-2S]		Rieske	
Mode	[mdyne/Å]	[kcal/molÅ^2^]	[mdyne/Å]	[kcal/molÅ^2^]
K(Fe-S(b)):His	1.353	195.	0.949	137.
K(Fe-S(b)):Cys	1.353	195.	0.874	126.
K(Fe-S(t))	0.587	84.	1.169	168.
K(Fe-N(t))	-	-	0.411	59.
H(Fe-S(b)-Fe)	0.303	44.	0.373	54.
H(S(b)-Fe-S(b))	0.410	59.	0.373	54.
H(S(b)-Fe-S(t))	0.460	66.	0.335	48.
H(S(t)-Fe-S(t))	0.460	66.	0.335	48.
H(S(b)-Fe-N(t))	-	-	0.401	58.
H(N(t)-Fe-N(t))	-	-	0.401	58.
K(Fe-Fe)	0.164	24.	0.246	35.

The notation K and H indicate stretching and bending, respectively.

The Mossbauer data shows that the unpaired electron tends to localize more on the His side of the Rieske structure [9,40], and the exchange is typically weaker [9,40,44,47] than the 4-Cys [42,43,45,68]. Localization is consistent with a zero formal charge of the His and charge delocalization of the electron within the His ligands, which would tend to encourage the electron to residue on the His side where it can transfer the electron to the ROC. Finally, it is important to reiterate that the asymmetric structure of the reduced Rieske in Fig 6C and 6D is the result of geometric optimization, where the orientation is similar to that observed in the protein structure. Together, these points suggest that the [2Fe-2S] is aimed at storing electric charge and storing it in a somewhat static way on the His side of the structure. Furthermore, natural structures of 4-Cys from Fdx also appear to have such an asymmetric configuration of the Cys residues. The QM studies on these structures showed almost no spin contamination–a characteristic problem with some density functionals and basis sets on the highly symmetric structures in Fig 5 [unpublished data]. This suggests that this asymmetry in the structure is also an evolutionary adaptation toward optimizing electron storage and localization on the active side of the electron transfer point.

For most of the His ring bending parameters, we substituted parameters from the GAFF force field whenever possible. Therefore, we needed only to obtain stretching and bending parameters for N-Fe-S, C-N-Fe (for the His ligands) and S-Fe-S and C-S-Fe (for the Cys ligands).

The energy contribution from dihedral angles is typically an order of magnitude smaller than the bending interactions and the ligands in [2Fe-2S] are constrained by the protein. Therefore, we focused on specific ring bending issues associated with the [2Fe-2S]. One out-of-plane bending in the [2Fe-2S] ring involving dihedral angles is Fe-S^(b)^-Fe-S^(b)^. Other examples are rotation and bending of the His-Nδ and Cys-Sγ rings: twisting of His and Cys around the [2Fe-2S] ring (Sγ-Fe-Fe-Nδ), a His wag (Fe-S^(b)^-Fe-Nδ), a Cys wag (Fe-S^(b)^-Fe-Sγ), rotation of His around the Fe–Nδ bond axis (S^(b)^-Fe-Nδ1-Cγ and S^(b)^-Fe-Nδ1-Cδ2), and rotation of the Cys around the Fe−Sγ bond axis (S^(b)^-Fe-Sγ-C). These parameters were estimated from rotating the residues and scanning the change in the energy. These assignments can be found in Listing 7 in S1 File (with details in Listing 8 in S1 File) and an example of rotation of His about the Fe-N bond axis is shown in Fig C (panel a) in S1 File.

#### ROC region

In general, the ROC structures contain two histidines (both bound at Nε), one Asp (usually in a bidentate configuration), and one bound H_2_O molecule (for five-fold coordination) or two bound H_2_O molecules (for six-fold coordination). However, the X-ray structures of 1WW9, 2DE5 (oxidized), 2DE6 (reduced), and 2DE7 (oxidized) all demonstrate a four-fold coordination around the Fe with two histidine molecules, one Asp (in monodentate configuration), and one bound water molecule (Fig 2B).

Building this structure and optimizing the Fe^2+^ state with *S* = 5 without any additional water molecules and without any constraints always resulted in a bidentate structure (Figs A and D in S1 File; panels b and a, respectively). We eventually discovered that water in the chamber helps stabilize the tetrahedral-like structures (Fig D in S1 File, panel b). Indeed, calculation of the partial charges required the inclusion of both the Fe-ligand complex and water molecules in the chamber region of the ROC structure (Fig 2B). Notably, there is a tendency for monodentate structures to appear in dOx proteins that have a considerable number of water molecules in the reaction chamber (e.g., 2DE5, 3GCF, and 3GKQ). Similarly, there is a strong tendency for chambers lacking water to exist in a bidentate configuration (e.g., 1NDO). The number of coordinating H_2_O ligands is also conditional on there being an excess of water in the chamber in general. For example, the chamber of the structure in Fig 3A and 3B has a similar number of water molecules surrounding the ROC [36] in the respective PDB files.

As a footnote, it is noted that the original study (Ref [36]: PDB 1Z01 and 1Z02) did not mention the differences in the number of coordinating water ligands. We have examined the energy differences of one and two bond water ligands (keeping the total number of water molecules equal to five) and found that the energy difference between the two optimized configurations to be less than 1 kcal/mol. Since the differences in oxidation state are more than an order of magnitude larger, this difference can be considered negligible.

Because the observed reaction chamber has water around this bond, we built the structure of the Fe center with the monodentate Asp, one bound H_2_O, and four additional unbound H_2_O molecules, and optimized the structure under a variety of conditions. The configuration of the water molecules was optimized by running variations of the structure and allowing the structure to optimize in a variety of charge and spin states. Fig 2B shows an optimized configuration of the ROC with four free water molecules around the Asp and one bound water ligand.

**Fig 8:**
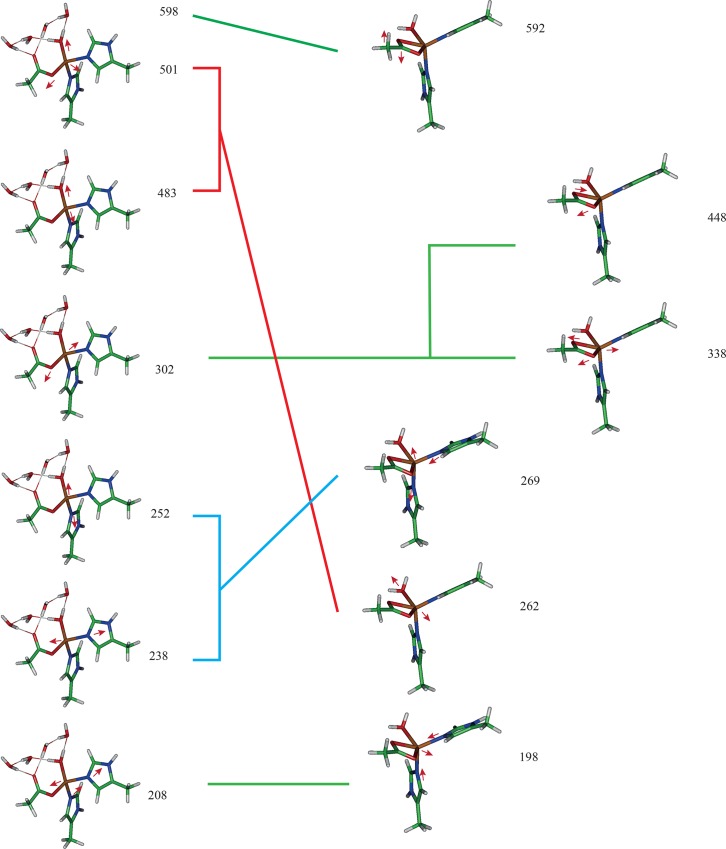
Break down of normal modes for the ROC. **(Left) Modes for the ROC with Asp in the monodentate configuration (including optimized unbound water molecules found in the chamber).** (Right) Modes for the ROC with Asp in the bidentate configuration (typically these chambers contain no water).

The decomposition of important normal modes, excluding the numerous modes involving free molecular water molecules, is shown in Fig 8. The bond stretching and bending parameters, which were extracted from the optimized structure using frequency analysis, are shown in Tables 9 and 10. Unlike the Rieske normal mode data, there are no direct measurements of stretching or bending in these Fe-center structures. The main available bidentate data are those from catecholate structures [69,70], and the main available monodentate data are those from tyrosine binding to Fe in a ligand structure [70]. However, the main Fe–Nε and Fe–OH_2_ interactions are not reported [33,69–71]. The extensive variability of coordination around the Fe also makes it difficult to obtain experimental values.

**Table 9:** Normal modes found in the ROC structure for the hydrated monodentate structure.

Chamber with water			Relationship
cm^−1^	mdyne/Å	kcal/molÅ^2^	
598	0.505	72.7	OAc modulation
501	0.607	87.3	H_2_O–Fe–OAc breathing mode stretch (Fe moves opposite to–OAc and–OH_2_)
483	0.439	63.2	Fe–OH_2_ stretch
302	0.262	37.7	Fe–OAc stretch
252	0.211	30.4	Fe–(Nd)His stretch
238	0.179	25.8	Fe–(Nd)His stretch
208	0.208	29.9	Fe–((Nd)His)2 bend (Fe moves opposite to both His structures

**Table 10:** Normal modes found in the ROC structure for the unhydrated bidentate structure.

Chamber without water			Relationship
cm^−1^	mdyne/Å	kcal/molÅ^2^	
592	0.547	78.7	OAc modulation
448	0.442	63.6	Fe–COO bidentate antisymmetric stretch
338	0.194	27.9	Fe–COO bidentate symmetric stretch
269	0.402	57.8	(Nd)His–Fe–(Nd)His antisymmetric stretch (Fe moving opposite to the two His residues)
262	0.309	44.5	Fe–OH_2_ stretch
198	0.147	21.2	Fe–His stretch

In Fig 8, the normal modes of the optimized monodentate with one bound water molecule and four free water molecules coordinated around the acetic acid (Asp) residue are shown compared to the normal modes of the optimized bidentate without any coordinated free water. These configurations all have **C**_1_ (type A) symmetry. The relationships and correspondences of the normal modes are shown by the lines joining the similar structures; green lines indicate exact correspondence of modes, red and blue lines indicate symmetry breaking normal modes due to the monodentate and bidentate structure of the bound acetate (Asp). The most significant difference is the mode associated with Fe–OH_2_ stretching, in which the value is almost doubled, probably because the water helps stabilize the structure. Numerous modes are observed from the free molecular water molecules (not shown); the free water molecules tend to coordinate with the acetate when the acetate moves against these molecules. The O–Fe–O bond angle in the bidentate is not ideal for an octahedron. The hydrogen bonding between the nearest neighboring Fe–O bond and the network of free water molecules tends to break the Fe–O bond, helps (further) stabilize the acetate, removes the strain of a bidentate, and permits a natural tetrahedral configuration around the non-heme Fe(II).

Dihedral angle parameters can be found in Listing 7 in S1 File (with details in Listing 9 in S1 File). These were handled in the same way as the Rieske structure; the methyl imidazole corresponding to His183 and His187 was rotated around the bond axis (every 30°) and the structure was optimized (Fig D in S1 File, panel b). Similarly, the monodentate Asp was evaluated for various angles around the monodentate bond axis. The internal bonds for the His and Asp force field parameters use standard GAFF parameters.

#### Rieske-Asp-ROC complex

The Rieske and Fe-center studied above are part of a system coupled with an Asp residue (Fig 2A, Fig 3A and 3B and Fig 4). The coupling requires the Asp to act as a bridge between the Rieske and the Fe center. Therefore, on a smaller scale, we were able to compute the [(Cys)_2_[2Fe-2S](His)_2_]^q^ − [Asp]^1−^ − [(His)Fe(His,Asp,H_2_O)]^1+^ + 4H_2_O network (Fig 4), where *q* = 0, −1. Three electronic structures with Fe^2+^ at the ROC were constructed and optimized. The first structure comprised the Rieske in the oxidized state *q* = 0:

$${\left\{{[\text{Fe}}^{3+}(s=5/2)\text{Cys},{\text{Fe}}^{3+}(s=-5/2)\text{His}]⊕{\text{Asp}}^{1-}⊕{\left[{\text{Fe}}^{2+}(s=4/2)\right]}^{1+}\right\}}^{Q=0,S=5}$$

where *s* defines the spin state of the specific Fe ion *Q* is the total charge of the complex and *S* defines the multiplicity of the complex. The Cys and His labels indicate the arrangement of the Fe states in the Rieske structure.

Two additional structures were constructed for the reduced state:

$${\left\{{[\text{Fe}}^{3+}(s=5/2)\text{Cys},{\text{Fe}}^{2+}(s=-4/2)\text{His}{]}^{1-}⊕{\text{Asp}}^{1-}⊕{\left[{\text{Fe}}^{2+}(s=4/2)\right]}^{1+}\right\}}^{Q=-1,S=6}$$

and

$${\left\{{[\text{Fe}}^{3+}(s=-5/2)\text{Cys},{\text{Fe}}^{2+}(s=4/2)\text{His}{]}^{1-}⊕{\text{Asp}}^{1-}⊕{\left[{\text{Fe}}^{2+}(s=4/2)\right]}^{1+}\right\}}^{Q=-1,S=4}$$

The configuration of the spin states were specified using the method Generalized Ionic Fragment Approach (GIFA) [6] with g03. The Fe^3+^ is placed on the Cys side because the two negatively charged MeS^−^ligands tend to encourage the unpaired electron to reside at the His side. The localization of the electron is supported by results from EPR, resonance Raman spectroscopy, and Mossbauer spectroscopy measurements [9,40]. After an initial set up using examples from Ref [6] to construct the spin state, these structures were then optimized at the OPBE/6-311+G(d) level and the electronic structure then evaluated at the OPBE/cc-pVTZ level using g09. The details of using GIFA are explained in Section 3 in S1 File.

Single point calculations of additional states were also carried out to compare different configurations. For example, the reduced Rieske state with the ROC in the Fe^3+^ state was also attempted

$${\left\{{[\text{Fe}}^{3+}(s=5/2)\text{Cys},{\text{Fe}}^{2+}(s=-4/2)\text{His}{]}^{1-}⊕{\text{Asp}}^{1-}⊕{\left[{\text{Fe}}^{3+}(s=5/2)\right]}^{1+}\right\}}^{Q=0,S=7}$$

using the reduced structure and assuming the Fe^3+^ state is highly transitory; i.e., happening so rapidly that the structure does not have time to adjust.

The spin and charge states of the Fe ions do not appear to change drastically between the oxidized and reduced state (Tables 11 through 14). Rather, the average spin on the bridge sulfurs (S^(b)^) and the ligand Sγ (S^(t)^) tended to shift according to the state of the complex. In the Mulliken spin distributions of the reduced structure, the charge and spin of the Fe^2+^ is largely unchanged. Therefore, the additional electron mainly resides on the Rieske structure, as indicated by the sign change between the *S* = 6 and *S* = 4 state (where the charge on the Fe^2+^ of the ROC remains essentially unchanged in both the oxidized and reduced states of the cluster).

**Table 11:** Partial charge and spin for the main components in the Rieske-Asp-ROC complex.

	**Oxidized**		**Reduced**		**Reduced**	
Atom	q = 0	S = 5	q = −1	S = 4	q = −1	S = 6
Rieske						
Fe(Cys)_2_	0.720	3.22	0.853	−3.26	0.800	3.27
Fe(His)_2_	0.867	−3.56	0.666	3.12	0.651	−3.15
S(b)	−0.553	−0.14	−0.789	−0.16	−0.754	0.18
S(b)	−0.593	−0.15	−0.807	−0.14	−0.805	0.14
S(t), Cys69	−0.632	0.31	−0.820	−0.27	−0.804	0.25
S(t), Cys90	−0.569	0.30	−0.677	−0.25	−0.647	0.25
ROC						
Fe^2+^ (ROC)	1.362	3.93	1.412	3.88	1.380	3.89

The numbering for the cysteine residues (Cys69/Cys90) corresponds to the structure of oxygenase (PDB 1WW9 and 2DE5–7). Here, *q* and *S* are the total charge of the complex and the total spin, respectively. The label ‘b’ means the bridge sulfur and ‘t’ means the ligand (tetrahedral) bond.

**Table 12:** Partial charges on the Rieske structure for the Cys residues. The numbering for the Cys residues corresponds to the structure of oxygenase (PDB 1WW9 and 2DE5–7).

ff label	Cys 69		Cys 90		Amber94
	Oxidized	reduced	oxidized	reduced	
CB	0.130	0.201	0.153	0.344	−0.241
HB2	−0.001	−0.027	−0.020	−0.088	0.112
HB3	0.012	−0.013	−0.011	−0.079	0.112
SG	−0.569	−0.677	−0.632	−0.820	−0.884

The parameters for the Amber94 partial charges (benchmarks) for HID and HIE are shown in the last column for additional comparison. Results for the reduced structure are similar for total multiplicity of the complex (*S* = 4 and *S* = 6, and ROC with Fe^2+^).

**Table 13:** Partial charges on the Rieske structure for the His residues.

ff label	His 71		His 93		Amber94
	oxidized	reduced	oxidized	reduced	
CG	0.087	0.042	0.410	0.327	0.187
ND1	−0.237	−0.099	−0.495	−0.524	−0.543
CE1	0.039	−0.029	−0.102	−0.092	0.164
HE1	0.162	0.179	0.196	0.168	0.144
NE2	−0.231	−0.281	0.182	0.175	−0.280
HE2	0.364	0.373	0.170	0.144	0.334
CD2	−0.126	−0.129	−0.408	−0.402	−0.221

The numbering for the His residues corresponds to the structure of oxygenase (PDB 1WW9 and 2DE5–7). The parameters for the Amber94 partial charges (benchmarks) for HID are shown in the last column for additional comparison.

**Table 14:** Partial charges on the His in the ROC.

ff label	His183		His187		Amber94
	oxidized	reduced	oxidized	reduced	
CG	0.321	0.261	0.220	0.255	−0.027
ND1	−0.325	−0.270	−0.196	−0.224	−0.381
HD1	0.377	0.362	0.350	0.351	0.365
CE1	0.120	0.121	0.041	0.103	0.206
HE1	0.143	0.148	0.148	0.140	0.139
NE2	−0.396	−0.448	−0.439	−0.542	−0.573
CD2	−0.198	−0.181	−0.128	−0.113	0.129

The numbering for the His residues corresponds to the structure of oxygenase (PDB 1WW9 and 2DE5–7). The parameters for the Amber94 partial charges (benchmarks) for HIE are shown in the last column for additional comparison.

The exchange coupling of the 4-Cys [45,46,68] is often twice that of the Rieske [43,44] and it can be much larger [47]. The relaxation rate also correlates with the exchange energy. The exchange energy is inferred from the two-phonon Orbach relaxation process, which clearly is observed in these systems for temperatures above 40 K. Vibrational modes in the [2Fe-2S] ring are of a similar order of magnitude as the exchange energy, suggesting the possibly that Orbach relaxation is coupling to modes in the [2Fe-2S] and may even resemble a phenomena known as phonon-assisted electron hopping [47], though the latter is usually associated with macroscopic electron conduction [72–79].

Based on the QM electron spin data derived from g09 and GIFA, the significant difference in the charge between the His side and Cys side of the [2Fe-2S] suggests that the electron is semi-localized on the His side. This is also suggested in the Mossbauer data [9,40]. A PCET mechanism implicitly would assume that the electron is largely localized on the His side of the [2Fe-2S]. Symmetry breaking features found in the physical orientation of the Cys ligands in proteins containing 4Cys-[2Fe-2S] also appear to help weakly localize the electron on one side or the other of the [2Fe-2S] ring (Dawson et al, unpublished data). Another feature that may influence the tendency of electron localization are examples of rare cases where a single Asp [80] or His [66] forms a coordinating prosthetic ligand as (Asp,Cys)[2Fe-2S](Cys)_2_ or (His,Cys)[2Fe-2S](Cys)_2_, respectively. This change of prosthetic ligand also may be important in examples of 4Cys-[4Fe-4S] where a His ligand was found in the place of one of the Cys residues [81].

### Benchmarking of partial charges

Since the Amber ff99SB partial charges (which are the same as the Amber94 *partial charges*) are derived from careful quantum chemistry calculations of isolated amino acid residues including the backbone chain at the neighboring amino acid positions [48,55,82,83], the Amber94 partial charges represent a limiting case for the non-interacting residues. In as much as the parameters of the non-interacting residues in this study approach those of the isolated cases derived in Amber94, they also compare how these interactions are changed by coupling—as in the case of His93. It is not only important to observe how the coupling is changed between the oxidized and reduced state, it is also important to see that this coupling remains largely *unchanged* (from the Amber94 standard) on residues that *are* treated as non-interacting. Moreover, some of the part of the present work is to meld it in with the Amber force field for doing MD simulation studies of dOx/Fdx proteins. Whereas there is room for discussion, these force field parameters are a *de facto* standard. Hence, for the purpose of comparing the partial charges, we will call the Amber ff99SB parameters (in general) the Amber benchmarks for short.

Predicted partial charges in this work are, at least to some extent, of similar weight to the Amber benchmarks. In particular, the residues–which appear to be isolated except for interaction with the Fe^2+^ or [2Fe-2S] (e.g., Cys69, Cys90, His71, His283 and Asp333)–are likely to have comparable weights to the Amber partial charges. For short, we will call such residues “non-interacting” because no further coupling occurs beyond the association with the Fe^2+^ or [2Fe-2S]. This is in contrast to His93 and His187 when the Asp180 bridges the interaction between His93 and His187. The Cys residues in the complex are not interacting with any of the surrounding residues in the cluster calculations. As a result, the partial charges on the Cys69 and Cys90 are very similar, as expected (Tables 11 through 14). The most consistent difference in the predictions of CHelpG appears mostly at the beta carbon position. We were not able to find a way to produce the common positive charge on the Cα atom in the Amber benchmarks with the current model.

Comparing the predicted partial charges in this work (Listing 5 and 6 in S1 File and S3 File) with those obtained for 4-Cys (calculated at the UHF/6-31G* level [56]), the magnitude of the charges tends to be even more polarized than in the Amber benchmarks when calculated at at the UHF/6-31G* level. The partial charges for the amide along the backbone (N-H), the Cα and the Sγ of the Cys are the most dissimilar to the partial charges predicted in the simplified Cys structure (thiomethane) in this study, and they are also larger than the Amber benchmarks (cf., Table 12, and Section 4.2 in S1 File and Listings 5 and 6 in S1 File). The magnitude of the charges of the carbonyl-group atoms is smaller than that found in this work and the Amber benchmarks. Aside from the binding Fe–Sγ interaction, for the most part, the results of the Cys residues of 4-Cys appear consistent with the Amber benchmarks except most notably for Cα.

The similarity between the Amber 94 partial charges and those obtained using CHelPG show that CHelPG can be used to obtain these parameters with reasonable correspondence. Further details on the assignment can be found in Methods and in Section 4 in S1 File.

In the cluster calculation in Fig 4, we have focused on the coupling between the His residues in the structure, leaving the environment and symmetry of the Cys residues unchanged from the isolated Rieske shown in Fig 2C and Fig 6A and 6B (c.f., Fig 4). Obviously, the Cys residues interact with other residues in the protein structure and this non-interacting picture is highly idealized. Nevertheless, this is an important test of the model because we have aimed at assigning partial charges such that the non-interacting residues (defined earlier) approach the Amber benchmarks as closely as possible; e.g., Cys69, Cys90, His71, etc.

Comparing the His ligands on the Rieske structure (Table 13), the His93 that couples with the Asp180 through the His93:(Nε2,Hε2) and the carboxyl of Asp180 show a significant change in partial charge compared to His71 that has no bridging interactions with other residues in the cluster. Moreover, with the exception of atoms near the Fe (Nδ1, Cγ and Cε1), the partial charges on His71 resemble the Amber benchmarks far more than His93 (Table 13, Listings 5 and 6 in S1 File and S3 File). The partial charge of His93:Nε2 is positive in Table 13, whereas the corresponding His71 and Amber benchmark is negative. These changes in the partial charge distribution appear to be largely due to the proximity of the carboxyl Oδ of Asp180.

The remaining Asp333 ligand has a partial charge similar to that of the Amber benchmarks. This drastic shift of the His residues joined by the Asp180 coupling would render the interactions rather attractive. There is also a subtle variation in the partial charge of the Nδ1 of His183 and His187 between the reduced and oxidized structures (Table 14, compare columns 2 and 3 for Nδ1 of His 183). Although the difference between the oxidized and reduced partial charges (compared to the isolated Rieske and ROC) is not quite as dramatic as His93, the reduction in the negative charge of Nδ1 (of His183) would make the interaction between the carboxyl of Asp180 statistically more favorable. This, in turn, suggests that these molecular orbital interactions and charge differences may facilitate the ET. The coupling of Asp180 causes the partial charge on the His93:Nδ2 to change significantly between the reduced and oxidize states of the Rieske. In the reduced sate of the Rieske, the His183:Nδ1 partial charge becomes less negative while the Hδ1 remains essentially constant. This in turn helps attract the carboxyl Oδ of the Asp180 when the Rieske is reduced.

For the non-interacting atoms, the Amber benchmarks and the refined partial charges are similar. This is likely to occur when there is no significant interaction via bridging residues such as Asp180. This also shows that similar partial charges are obtained when the histidines are involved in more generic intra-chain interactions with other amino acids. Because there is a reasonable match to the isolated case (the Amber benchmarks) when residues are treated as isolated, and because there are observed deviations when the residues are clearly coupled, this study is tantamount to revealing the coupling within this protein environment. In as much as this limit can be objectively reached from the techniques used here, the relationship between the partial charges becomes more informative for the structures that deviate from the systematically tuned Amber benchmarks.

Fig 3 shows the experimentally observed results of two structure of 2-oxoquinoline-8-monooxygenase oxygenase (OMO): an oxidized structure (1Z01: Fig 3A) and the reduced form (1Z02: Fig 3B) [36]. Here, references to residues in 1Z01/2 are specified using the label “OMO:” unlabeled residues are assumed to be those of the CRDo protein. In Fig 3, the interaction of OMO:Asp218 would change from a more repulsive interaction with OMO:His221 for the oxidized complex (Fig 3A) to an attractive interaction for the reduced complex (Fig 3B). Hence, binding of the OMO:Asp218 to the OMO:His108 and OMO:His221 in the reduced state is stronger than in the oxidized state. Consequently, as shown in Fig 3B, the OMO:Asp218 would probably bridge between OMO:His108 and OMO:His211 in the reduced state. Similarly, in Fig 2A, binding in the bridge formed by CRDo:Asp180 should be stronger with CRDo:His93 and CRDo:His183 in the reduced state. The results are consistent with the observed behavior of the complex (Fig 3A and 3B).

We have employed quantum chemistry to observe the electronic structure of CRDo:His93 and His183 when the side-chain carboxyl group of Asp180 bridges His93:Hε2 and His183:Hδ1. For the Fe^2+^ state with one H_2_O ligand bound to the ROC, there is only a subtle change in the partial charges of the ROC. This change involves the bridging Nδ1 of His183 and favors bridging of the carboxylate in the reduced state. Significant differences in the partial charge of the His71 and His93 are evident. Indeed, when the Rieske and ROC are evaluated for the uncoupled system (the individual isolated Rieske and ROC structures), this difference between equivalent residues disappears, indicating that it is a long range interaction between the residues.

### Force field testing of Rieske and ROC structures

After an initial set of parameters was determined, short MD simulations were carried out to ensure that the structure around the Rieske and the Fe center was maintained during simulations at ambient temperatures. The purpose of this Section is simply to show that the structures behaved as expected. A far more detailed test of the force field will be reported elsewhere.

**Fig 9:**
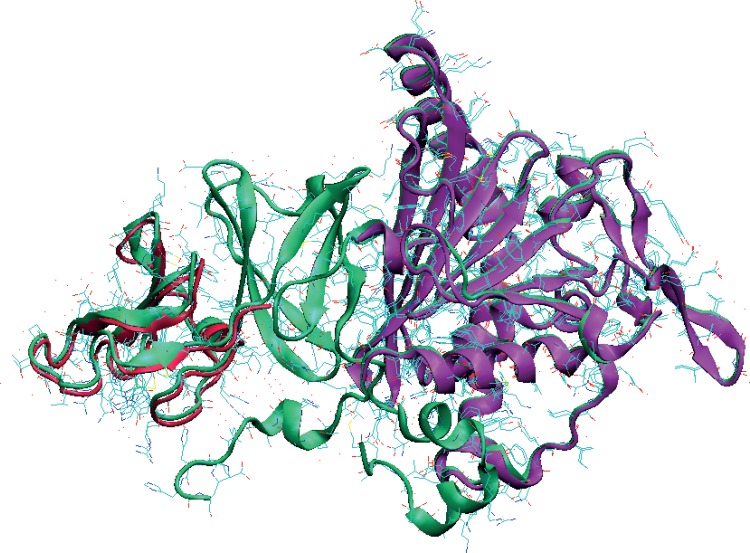
Substructures of 1,9a-dioxygenase (PDB id: 1WW9) that were used as an initial test of the force fields. More extensive tests of the full complex (PDB ids: 2DE5-7) will be reported elsewhere. Left(red), the Rieske region with [2Fe-2S]. Right(purple), the region encompassing the mono-nuclear Fe structure. The [2Fe-2S] half is the structure from PDB id 3GKQ (a related 1,9a-dioxygenase from Sphingomonas, strain KA1 which uses a 4-Cys-[2Fe-2S] Ferredoxin cognate). The simulations are shown in Fig 10.

To test force field parameters, a small substructure of the dOx was constructed. For the Rieske structure, a fragment of 3GKQ that contains the region of the [2Fe-2S] was extracted from amino acid position number 68 to 128 in the PDB file. The cut points were determined based on a visual inspection of the structure for separable domain regions (Fig 9, left, red). For the ROC, a fragment of 1WW9 (144 to 375) that contains the full ROC was extracted. Here, the cutting points were also based on a visual inspection of the structure (Fig 9, right, purple).

Subsequently, these substructures were tested in short MD simulations (with no constraints applied to the protein structure) to verify whether the optimized ligand complex concurred with the original measured X-ray structure at low temperatures. Moreover, test were carried out to verify whether the configurations will relax back into the X-ray structure if the structure is warmed to ambient temperatures and then sampled at random. Frequent reproduction of the low-temperature configuration in the bulk of the high-temperature samples clearly indicates that the force field does not distort the observed native state configurations. The final structures were compared with the initial structures to verify whether they were similar around the Fe-center. The structures were cut from the original dOx protein; thus, there were somewhat predictable distortions in the overall 3D structure during the run.

**Fig 10:**
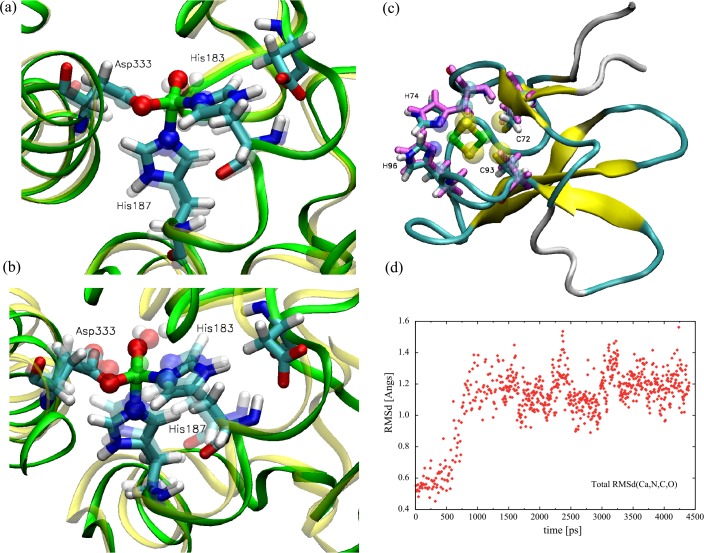
Snapshots taken after a short simulation time showing the variation of the structure from the original PDB file (id: 1WW9). The simulations were carried out using using the force field parmeters developed in this work. (a) The subsequence of dOx comparing the minimized initial structure with the PDB structure (transparent residues). (b) The same substructure of 1WW9 after a short 4 ns MD simulation. Whereas the *truncated structures* tend to distort slightly during the simulation (as expected), this shows that the structure around the Fe center remains stable and relatively unchanged from the original PDB structure (transparent residues). (c) The same study for the Rieske region using a subsequence fragment of a related 1,9a-dioxygenase (PDB id: 3GKQ). The transparent residues are associated with the initial pdb data. (d) The total RMS deviation from the x-ray structure for a full MD simulation of the 3GKQ fragment using the developed force field parameters.

The ROC structure after minimization is shown in (Fig 10A), and a snap shot of the structure after a 4 ns simulation in Fig 10B, where the more transparent residues in the Figure correspond to the initial PDB structure. The orientations of the residues within the ROC are largely unchanged during the simulation (Fig 10B). Perhaps the terms that can most distort the active site are the settings for the bond angles. The distortions in the complex were eventually limited to a root-mean-square deviation (RMSD) of less than 1.3 Å.

The same optimization was also carried out for the Rieske structure using a fragment of 3GKQ (Fig 9, left, red and Fig 10C and 10D). The results after a 4 ns simulation are compared with the original X-ray structure in the PDB file (Fig 10C). Thus, a stable structure could be obtained with the force field parameters of this system, at least within a time period of 5 ns. A full simulation is shown in Fig 10D, where the RMSD largely settles down in the last 4 ns of the simulation. The RMSD around the Rieske site was also limited to less than 1.3 Å.

These are certainly very short simulations that are not able to test the long term stability of the force field. In fact, the force field has been tested in the full (dOx)_3_:(Fdx)_3_ for stability up to 50 ns. These results will be reported elsewhere.

S4 File provides a zipped folder containing an example of a completely prepared 2DE5 complex (2DE5o_fixed.pdb) and scripts for building the 2DE5 complex in a water box, carrying out the warmup cycle (from 0 K), equilibration cycle and then a full scale run for as long as the user desires. The batch submission script must be modified to local system requirements or to operate on one’s local machine. Using these scripts, we have carried out several simulations on these complexes out to 50 ns.

The development of MD simulation parameters is largely an art where one must balance the practical realities of a doable simulation with the weakness of water and ion models, the massive simplification of normal modes and partial charges with QM, pH effect, etc [84]. The task here was no different. Using the files and the scripts in the S4 File, one can see how we chose to achieve that balance. For example, the QM suggests that the water is more networked than the TIP3P water model supports. What we can safely say is that these parameters have been tested multiple times to 50 ns.

### Donor-bridge-acceptor model for the Rieske-Asp-ROC

In the section titled “Benchmarking of partial charges”, we showed that the QM calculations generate partial charge distributions in isolated residues (e.g., the isolated Rieske and ROC units) that resemble the standard values that are found in the Amber ff99SB with g09 at the OPBE/cc-pVTZ level using CHelPG. When the residues interact, the electron density changes significantly, yet the non-interacting ligands in the complex have nearly the same electron density as the isolated case. As discussed in Section 4.5 in S1 File and, based on discussion and examples in the sections that precede it, this meant that the Cys ligands on the Rieske could be treated as equivalent fragments with the tag CYR (Listings 5 and 6 in S1 File). However the His residues had to be separated into the fragments HR1 and HR2 where electron density in HR1 is similar to the isolated case, but HR2 is coupled with the Asp and the ROC. Likewise, the His ligands on the ROC are divided into HI1 and HI2 where HI2 is the non-interacting residue and exhibits a similar electron density to the isolated ROC.

These interacting parts act collectively on the [2Fe-2S] ring, HR2, HI1 and the Asp positioned in between, and the non-heme iron. The Asp residue tends to change the electron density of the ROC and the [2Fe-2S] parts of the clusters. Experimental studies have shown that the Asp is essential for the reactivity of the ROC in naphthalene dioxygenase when the corresponding mutation Asp205Ala is introduced [37], whereas there was no detectable evidence of ET in the mutated sample. Similar results were also suggested for phthalate dOx [38] and toluene dOx [39]. If the ET mechanism relied exclusively on electron tunneling, then changing the residue would not completely suppress the process. The distance between the Rieske iron on the His side and the ROC iron is about 12 Å; a distance that is much shorter than many photosynthetic materials that are known to use an electron tunneling mechanism [85,86]. Therefore, the charge on the Asp changes the electron density of the non-heme iron, the [2Fe-2S] and the His residues that connect it. The OMO [36] also clearly changes orientation when oxidize or reduced (Fig 3). Finally, experimental evidence has been found for fast PCET in an emulated (His)_2_[2Fe-2S] (Cys)_2_ structure, where a phenylbis(benzimidazolyl)-methane ligand replaced the two His ligands and a 1,1′-Biphenyl-2,2′-dithiolate ligand replaced the two Cys ligands [87,88].

Taken together, the experimental evidence and the long range coupling obtained here suggests the Asp residue is not merely the conditional packing of the protein structure, rather it is in integral part of a donor-bridge-acceptor complex [89,90], where the Rieske is the donor, Asp is the bridge, and the ROC the acceptor.

In PCET systems, the H-bond can supply the network of the coupling needed and lower the barrier to electron transport. If electron transfer was the result of tunneling, then the Asp would not have a major influence on this process. In fact, the process is killed when the Asp is missing. This *suggests* that the ET is modulated (or collimated) between His93 (Rieske) and His183 (ROC) through the Asp180; a characteristic of PCET systems [13,15,91].

## Conclusions

In this study, we have used quantum chemistry to examine the coupling in oxygenase (Ox) proteins between the ligands binding the [2Fe-2S] in a Rieske configuration (His)_2_[2Fe-2S](Cys)_2_ and the ligands binding the non-heme mononuclear iron site (ROC, (His)_2_[Fe^2+^]Asp) via the bridging Asp. Whereas the approach was directed to a specific dioxygenase protein involved in oxidizing carbazole at a stereospecific position, the approach is widely applicable to a wide varieties of PAH-related Ox proteins. The quantum chemistry revealed changes in the partial charges when a ligand bound to the Rieske [2Fe-2S] ring was coupled to the Fe^2+^ center via an Asp bridging molecule and a His acceptor bound to the non-heme Fe. These changes also depended on the oxidation state of the Rieske. This has strong implications for assigning partial charges inside an intensely hydrogen-bonded network of conjugated and aromatic bonds such as the protein structure currently under study. This study indicates that the electron density is likely to change as a result of the H-bond network of residues in the protein structure. Subtle changes in the size of the structures also result from changes in the electron density in the [2Fe-2S] ring: the oxidized and reduced states of the Rieske. It may be that the small changes in the size of the modules and the change in electrostatic interaction help influence the switching between different electronic states within the dioxygenase (dOx) structure and the affinity between dOx and its peripatetic ferredoxin partner. The results of this study were used to develop a force field for this complex. Further, the results of the bridging Asp appear to be consistent with networks involving proton coupled electron transport mechanisms.

In a subsequent report, we have taken these findings further in examining the processes associated with the redox center (ROC). The current force field design has been tested to the time range of 50 ns.

## Methods

### Structure construction and quantum chemistry methods

To build the structures consistent with configurations observed in the protein environment, coordinates for the carbazole 1,9a-dioxygenase (CRDo) structure were obtained from the Protein Data Bank (PDB): accession numbers 2DE5, 2DE6, and 2DE7.

Difficult optimizations were first carried out using Gaussian 03 (g03) [92] because they required weeks or even many months of wall clock time on a dedicated machine to converge. After this initial optimization, electrostatic potentials and force fields were later determined using Gaussian 09 (g09) [92,93].

All calculations used density functional theory (DFT) [94] at the BP86/6-311+G(d) and OPBE/6-311+G(d) [95–99] and OPBE/cc-pVTZ [100–102] levels. Selection of the basis set and the BP86 DFT [103,104] approach were based on conclusions drawn from Refs [6,51] and from testing and comparison of results. Selection of OPBE (OPTX exchange [67,105] with PBE correlation [106,107]) was based on the reported charge and spin results of the Fe ions in Ref [100]. For individual substructures (e.g., the Rieske), other DFT and basis sets were also verified: B3LYP/TZVP [108–110], B3LYP/6-311+G(d), BP86/6-311++G(d), BP86/6-311+G(3df,3pd) [95], BP86/cc-pVTZ, BP86/AUG-cc-pVTZ as well as the relativistic version of cc-pVTZ [111]. OPBE yielded results comparable to BP86 for the 6–311+G(d) basis set. Finally, due to the far greater overall stability in the basis set and density function, OPBE/cc-pVTZ was used exclusively in the large Fe complexes in the final results.

### Construction of the complex

The structures and force fields (described within this Section) were built up stepwise. First, simplified molecules of the Rieske and ROC structures as shown in Fig 2C and 2B, respectively, were built by optimizing the structures modeled based on the CRDo structure. Second, the complete Rieske-Asp-ROC interaction (Fig 4) was replicated by combining the separately optimized Rieske and ROC with an optimized acetate anion and arranging the structure in the approximate configuration as seen in the protein structure (using Molden v4.8 [112]). The isolated simplified molecules were also used to obtain frequency data and the approximate partial charges.

#### Optimizing the Rieske structure

To construct the Rieske structure, a methyl thiolate anion was built to approximate the essential electronic structure properties of the cysteine (from the beta carbon). Similarly, based on the crystal structure arrangement, a methyl imidazole (4-methyl-1,3-diazocyclopenta-2,4-diene) was constructed to approximate the essential properties of the histidine (from the Cβ position) and was affixed at the Nδ1 (IUPAC imidazole N3) position to the [2Fe-2S] ring (Fig 2C). The structure was optimized at both the BP86 and OPBE/6-311+G(d) levels. Moreover, the resulting structure was further optimized using the GIFA method [6] to reflect the proper spin structure of the Fe^3+^ (spin ±5/2) in the [2Fe-2S] ring: S = 1 multiplicity, where each Fe^3+^ is arranged with an antiferromagnetic (AF) spin configuration. The reduced state with Fe^2+^ (spin −4/2) and Fe^3+^ (spin +5/2) was also constructed and optimized in the same manner, where the ferrous iron is known to be localized on the His side [9,40]. These Rieske structure results were also compared using the ORCA package [113–115], which allows direct evaluation of broken symmetry in AF systems [116,117]. Final structures were redone at the OPBE/cc-pVTZ level using g09 and GIFA and all results are reported from these calculations.

#### Optimizing the reactive oxygen center (ROC) structure

Irrespective of the water coordination, the mononuclear Fe site is known to be Fe^2+^ in the rest state (Table 1) [1,35,118]; the ligand-bound side-chain carboxyl group of Asp (Oδ) forms either a monodentate or a bidentate structure. The His-imidazole ring binds to the Fe^2+^ at the Nε (IUPAC: N1) position, and water coordinates with the Fe^2+^ as one or two additional ligands, depending on the particular environmental conditions. Therefore, the resulting configuration of the ligand bonds can be tetrahedral (monodentate with one water), trigonal bipyramid (bidentate with one water molecule or monodentate with two water molecules), or octahedral (bidentate with two water molecules) [5,35,118,119].

For the mononuclear Fe, 2DE5–7 contain only a single coordinated water molecule and a monodentate Asp in a tetrahedral configuration. Hence, this study will mainly focus on this system with a single bound water and monodentate Asp configuration, although the bidentate cases were also examined. The bidentate and monodentate structure of the Asp ligand at the ROC, although important in modeling the normal modes, do not otherwise strongly affect the conclusions drawn in this work.

The unconstrained optimized state of single bound water yields a bidentate Asp (Fig D in S1 File, panel a); the monodentate only occurs in the presence of water (Fig D in S1 File, panel b). Therefore, four additional free water molecules were added to the monodentate complex around the Asp, consistent with the observed X-ray structure. Moreover, the whole complex was optimized by running the structure through multiple structural states induced by changes in the charge and spin state of the Fe. These changes in charge and spin caused the ligands to rearrange significantly. Eventually, this cycling yielded a reproducible structure suggesting optimization (Fig 2B). The details of this optimization process will be discussed elsewhere.

#### Building and optimizing the Rieske-Asp180-ROC structure

A composite structure was built based on recent work on the water interactions and experimental studies of a related complex of dOx (PDB: 1Z01, 1Z02, and 1Z03) (Fig 3A and 3B). The complex demonstrated that the Asp residue (1Z01:Asp218) connected to His (1Z01:His108 and 1Z01:His221) when the Rieske domain is reduced (Fig 3B). Thus, by building the complete complex and optimizing under minimal constraints, the difference in the partial charges can be tested for the oxidized and reduced states of the Rieske or the mononuclear Fe. The method for obtaining the proper spin in the Fe was achieved using GIFA with g03 to determine the orientation of the spin in the structure. GIFA offers considerable control over the arrangement of the spins [6]. Details on the procedure for building the structure are provided in Listings 1 and 2 in S1 File.

### Development of force field parameters

For standard amino acids, the Amber ff99SB force field was used. The Rieske structure and the ROC both require additional force field parameters to express the ligands binding to the [2Fe-2S] Rieske structure (Fig 2C) and the ligand binding to the mononuclear Fe (Fig 2B). Both the oxidized and reduced forms of the structure were developed to express the Rieske-ROC complex.

#### Bond, angle, and dihedral angle parameters

Whenever it was possible to use Antechamber (Amber 10 modeling toolkit [52,120]) to obtain equivalents for the bond, angle, and dihedral angle parameters, the equivalents were substituted. Theoretical bonding parameters (stretching, bending) were obtained from Gaussian 09 (g09) using the Freq option with scaling adjusted to fit experimentally obtained vibrational frequencies.

Parameters for stretching and bending were determined by decomposing the calculated normal modes using the Molden 4.8 application [112] and matching these frequencies with the observed symmetry and corresponding normal modes of the structure. The calculated normal modes and force constants were scaled and benchmarked to known experimental data (when available) to obtain the best estimate.

For selected normal modes, these values were also estimated using finite difference techniques to evaluate the second derivative. To measure in this way, 5 to 7 structures were used and the geometry of each structure was built separately using Molden 4.8 and optimized using the starting electronic guess from the previous incremental solution (starting from the optimal structure). All atoms were left free except for those directly associated with the bond stretching mode of interest and those involved in dihedral angles around the ligand-[2Fe-2S] bond (to prevent unwanted rotations of the ligands). The approach was tested on the bonds Fe-Fe, Fe-S(b) (the bridge sulfur), and Fe-St (the tetrahedral sulfur, binding the ligands).

Dihedral angle parameters were determined by changing the respective angles of the ligands: rotating or changing the bond angle of various substructures of interest, constraining the respective angles, optimizing the structure, and comparing the change of energy. One of the His-imidazole rings bound to the Fe in [2Fe-2S] was rotated around the N–Fe bond axes through 360° (30° angles) to obtain the basic tendency of residues uninhibited by the surrounding protein structure. The initial electronic structure guess for the calculations came from the previous consecutive solution in the series. One dihedral angle constraint was applied to the rotated residue and another to any closely neighboring residues; i.e., a maximum of three dihedral angle constraints were applied to the ROC, and two dihedral angle constraints were applied to the Rieske structure. Optimizing the structures with a minimum number of *dihedral angle* constraints ensured that the energy differences reflected actual steric effects in the bonds rather than errors due to distortion in bond lengths and angles. The changes in the bonding of the structure due to rotation of the His are rapid; thus, the ligands have sufficient time to adjust to any mutual changes in the electric field due to the surrounding molecules in the neighborhood. Subsequently, the data were plotted and an approximate trial and error fit using Gnuplot was made based on the observed energy curve (g09 calculations). The results were compared with the corresponding Amber99 data when available.

Further details about on constructing the vibrational parameters and dihedral angles can be found in Section 4.4 in S1 File and the Amber frcmod files can be found in Listing 7 in S1 File. Some further information about the parameters in Listing 7 in S1 File are explained briefly in Listings 8 and 9 in S1 File. Example scripts and files for running Amber simulations can be found in Listings 10 and 11 in S1 File.

#### Partial charges

Partial charges were determined using the RESP charge calculation in Antechamber (Amber 10 modeling toolkit [52,120]) and CHelpG [53,54]. Because these partial charge calculations involve Fe ions and molecules of H_2_O surrounding the ROC chamber, both RESP and CHelpG were compared. To achieve RESP2 type charges, the CHelpG results for chemically equivalent atoms were averaged in the structure, e.g., the Hβ atoms on the Cβ of the amino acids.

Partial charges were obtained from larger structures; e.g., c.f. Fig A in S1 File for “truncated” and “extended”. These were constructed by starting with the already optimized simpler structures from the β carbon of the side chain as a starting template (the “truncated” structure), and mimicking the backbone of the amino acid chain by affixing the structure CH_3_(C = O)NH-CαH-(C = O)NHCH_3_ at the Cα position to the Cβ part of the side chain to build the extended part (Fig A in S1 File); i.e., the amino acid back bone with a terminal formyl group on the N side and a methylamine at the C side. This procedure was similar to that reported in ref [48], where an acetyl and methylamine structure were added on the N side and C side, respectively. The structures were optimized to obtain the partial charges in the full amino acid structure of the various residues of interest here. The partial charges of equivalent atoms were averaged. Fitting the structures generally followed the approach used by Weiner et al. [82,121].

Further details about fitting of the partial charges can be found in Section 4 in S1 File and S3 File. The Amber prepin files can be found in Listings 5 and 6 S1 File for the oxidized and reduced structures, respectively. Scripts and MD simulation files for running Amber simulations can be found in Listings 10 and 11 in S1 File.

### Molecular dynamics simulations

To test the force field parameters, molecular dynamics (MD) simulations were carried out in explicit water (TIP3P) [122] using the Amber10 program [52]. The complete [2Fe-2S] X-ray structure contains water molecules both at the surface and inside the protein complex, and they were retained unaltered.

Amino acid ligands binding the Rieske and ROC were relabeled in the corresponding PDB file and bonds were specified to include the force field in the calculations. Using an in-house application, the histidines were examined for neighboring donor hydrogen bonding residues in the PDB file, and the residues were assigned HID, HIE, or HIP accordingly: depending on the number of interactions and their location relative to Nδ1, Nε2, or both, respectively.

To the X-ray determined structure including the water molecules, counter ions (in this case Na^+^) were added such that the net charge of the whole complex was set to zero. Moreover, precautions were taken to ensure that Na^+^ did not replace the water in the X-ray crystal structure, particularly the internal water molecules. After adding the Na^+^, the whole structure was optimized for 2000 steps to remove any conflicting residue and water-ion interactions. Subsequently, a water box with a box border of 10 Å was added. The complete structure was again optimized for 2000 steps to remove major conflicting interactions with the water.

Warm up, equilibration, and production runs were carried out using SHAKE [52,123] with a 2 fs time step and at constant pressure (1.0 × 10^5^ Pa) in a water box stopping the center-of-mass motion every 10 ps [124]. The Langevan thermostat (with a damping factor of γ = 0.5 s^−1^) was used throughout [52,125,126] because the small damping weight appeared to be more stable than the Berendsen thermostat [127,128]. The warm up comprised a slow, continuous, and linear heating from 0 to 300 K over 100 ps. Warm up was followed by an equilibration period of an additional 100 ps to equilibrate the small fragment. Production runs were carried out for approximately 4 ns after the 100 ps equilibration. The structures were then aligned to identify the changes in the structures relative to the original PDB file. These were then tested with much longer simulations of 40 ns on the full (dOx)_3_:(Fdx)_3_ complex.

In this initial study, the molecular dynamics (MD) simulations were limited to runs testing the force fields to ensure that there were no significant distortions generated by the constructed force field, particularly to the chain regions of the protein. (Note that the test structures used here were relatively small. For a full 2DE5 complex, the warm up time should be extended to 600 ps to help minimize possible fracturing of the interfaces on the complex due to a rapid heating phase.)

## Supporting Information

S1 File Development of force field parameters for [2Fe-2S] and non-heme Fe and the respective complex. This file contains additional details on the methods used and some additional figures not included in the main text.
(DOCX)

S2 File Comparisons of the theoretical and experimental spectroscopic data. This file contains various tables used to construct the average bond length for the Rieske, tables showing all the available experimental data, fits of the raw g09 data to the experimental data, etc.
(XLSX)

S3 File Method for setting the partial charges and comparing the results with the standard AMBER force field parameters. This File shows how to build the partial charge model from the large cluster, the extended small clusters, and compares the results with established force field partial charges.
(XLSX)

S4 File Set up scripts for MD simulations. This file is a folder containing all the scripts and files necessary to build and use the force field reported in this work: an example of a completely prepared 2DE5 complex (2DE5o_fixed.pdb) and scripts for building, warming up, equilibrating and evaluating the full 2DE5 complex. Similar scripts are also in S1 File under the title “Listings”. These are provided as an aid in understanding how to apply the information in this study.
(ZIP)

## Abbreviations

AF: antiferromagnetic
CHelPG: CHarges from Electrostatic Potentials using a Grid
CRDo: carbazole 1,9a-dioxygenase (PDB id: 1WW9)
CRDf: the ferredoxin cognate of CRDo (PDB id: 1VCK
CRD: an identifier for the CRDo/CRDf complex (PDB id: 2DE5,6,7)
DF: density functional
dOx: dioxygenase
EPR: electron paramagnetic resonance
ET: electron transfer
FAD: flavin-adenine-dinucleotide
Fdx: Ferredoxin
GIFA: Generalized Ionic Fragment Approach
g03: Gaussian 03
g09: Gaussian 09
H-bond: hydrogen bond
mOx: monoxygenase
Ox: oxygenase
PAH: polyaromatic hydrocarbons
PCET: proton coupled electron transfer
QM: quantum mechanics/quantum chemistry
ROC: reactive oxygen center (non-heme iron and the corresponding ligands)
