# Supplementary material for: Electron Transport in a Dioxygenase-Ferredoxin Complex: Long Range Charge Coupling between the Rieske and Non-Heme Iron Center
Source: PLoS One. 2016 Sep 22;11(9):e0162031. doi: 10.1371/journal.pone.0162031 (PMC5033481; doi:10.1371/journal.pone.0162031)
Supplement: S1 File — This file contains additional details on the methods used and some additional figures not included in the main text. (DOCX) [file pone.0162031.s001.docx]

Electron transport in an dioxygenase-Ferredoxin complex: long range charge coupling between the Rieske and non-heme iron center

S1 File: Development of force field parameters for [2Fe-2S] and non-heme Fe and the respective complex

Wayne K Dawson^1,2,3*^, Ryota Jono^4^, Tohru Terada^1, 5^, and Kentaro Shimizu^1^

^1^Department of Biotechnology, Graduate School of Agricultural and Life Sciences, The University of Tokyo, 1-1-1 Yayoi, Bunkyo-ku, Tokyo 103-8657 Japan

^2^Laboratory of Bioinformatics and Protein Engineering International Institute of Molecular and Cell Biology in Warsaw,​ul Ks. Trojdena 4, 02-109 Warsaw Poland

^3^Laboratory of Functional and Structural Genomics, Centre of New Technologies, University of Warsaw, Banacha 2C, 02–089 Warsaw Poland

^4^Research Center for Advanced Science and Technology, The University of Tokyo Komaba, Meguro-ku, Tokyo 153-8904 Japan

^5^Agricultural Bioinformatics Research Unit, Graduate School of Agricultural and Life Sciences, University of Tokyo, 1-1-1 Yayoi, Bunkyo-ku, Tokyo 103-8657 Japan

*Corresponding author:

e-mail: [wdawson@genesilico.pl](mailto:wdawson@genesilico.pl) (WKD), [dawson@bi.a.u-tokyo.ac.jp](mailto:dawson@bi.a.u-tokyo.ac.jp) (WKD)

# 1 Introduction

The carbazole dioxygenase structures (PDB ids: 2DE5, 2DE6 and 2DE7) consist of both ferredoxin and dioxygenase. For all these structures, the complex is a trimer of dioxygenase molecules where each of the ferredoxin molecules is found bound to the surface of dioxygenase trimer in a region where two dioxygenase molecules interface each other (Fig 1, main text). The 2DE5 consists of the oxidized form of the Rieske structure: (Cys)_2_[2Fe-2S](His)_2_. To describe the location of the charge and spin state on the iron, the following notation will be used to describe the 2DE5 Rieske structure

$$\{\text{Fe}^{3+}(s=+5/2)\text{Cys},\text{Fe}^{3+}(s=-5/2)\text{His}\}^{q=0,S=1}$$

where *s* indicates the spin state of the Fe, the labels Cys and His indicate the location of the iron in the Rieske structure (S1A Figure, panel a), *q* indicates the net charge of the cluster and $S$ is the multiplicity of the cluster. The 2DE6 is the reduced form of [2Fe-2S] with

$$\{\text{Fe}^{3+}(s=+5/2)\text{Cys},\text{Fe}^{2+}(s=-4/2)\text{His}\}^{q=-1,S=2}$$

The 2DE7 structure includes the carbazole in the reaction chamber region of the dioxygenase subunit and is in the oxidized state.

The non-heme iron consists of two His ligands positioned at right angles to each other, an Asp ligand forming a monodentate bond (single bond) with the Fe and a water ligand. (An Asp in the bidentate form is shown in S1A Figure (panel b) and examples of the non-heme site are shown in S1B Figure.) The approximate overall coordination is tetrahedral. The total charge and spin state of the ROC will be described $\text{\{Fe}^{2+}(s=4/2)\}^{q=+1,S=5}$ for the ferrous state and $\text{\{Fe}^{3+}(s=5/2)\}^{q=+2,S=6}$ for the ferric state.

The total charge ($Q$) and spin multiplicity ($S$) of the complex including the Rieske, ROC and aspartic acid sandwiched between them is written

$$\text{\{[Fe}^{3+}(s=5/2)\text{Cys},\text{Fe}^{3+}(s=-5/2)\text{His}]^{0}\oplus\text{Asp}^{1-}\oplus[\text{Fe}^{2+}(s=4/2)]^{1+}\}^{Q=0,S=5}$$

for the oxydized state of the Rieske, and

$$\text{\{[Fe}^{3+}(s=5/2)\text{Cys},\text{Fe}^{2+}(s=-4/2)\text{His}]^{1-}\oplus\text{Asp}^{1-}\oplus[\text{Fe}^{2+}(s=4/2)]^{1+}\}^{Q=-1,S=6}$$

for the reduced state of the Rieske.

Building a force field (ff) for the dioxygenase and ferredoxin requires a number of steps. First, one must find partial charges, stretching, bending and dihedral angle parameters for the Rieske structure and the non-heme Fe reactive oxygen center (ROC). Second, one needs to restructure the partial charges in terms of the full donor, bridge and acceptor complex of this electron transfer complex. Third, one must find a strategy to integrate all these components (the ligands, Rieske structure and ROC) into the protein chain.


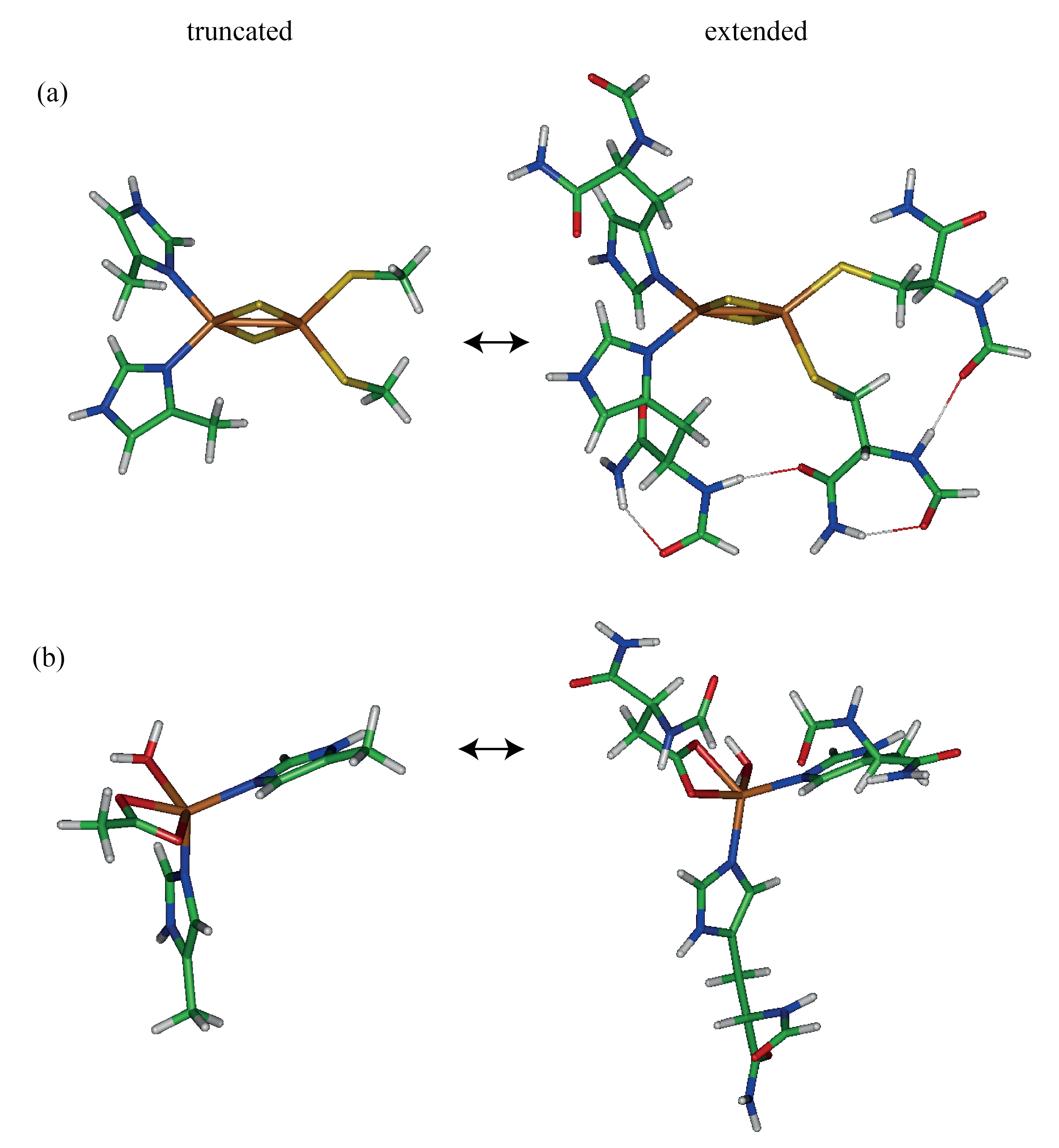


**S1A Figure. The truncated structures and the extended structures used in the calculations.** (a) the Rieske structure in truncated and extended from and (b) the non-heme Iron center in truncated and extended form.

Ideally, one would just do simulations using quantum chemistry (QC) to avoid the approximations used in molecular dynamics (MD) simulations. The entire complex could then be modeled with the full electronic structure. One would then simply identify the partial charges in the electron transfer (ET) network found between the ferredoxin Rieske and the dioxygenase Rieske (Fig 2, main text). However, optimization of such a large complex remains problematical with the computational resources currently available and the level of accuracy or resolution achievable: it is intractable to compute the entire electron transport network all the way from the ferredoxin to the Fe center with all the bridging amino acids and the iron complexes because optimizing structures with more than 200 *atoms* is generally quite difficult even using g09. It is also questionable to incorporate excessive detail in the ff because the molecular dynamics (MD) simulation technique is a semi-empirical model that must settle for a tenuous compromise between Newtonian mechanics and quantum chemistry.

Therefore, we aimed at a small subsection of the network: a critical junction where the size of the problem was manageable with current computational facilities. The problem was broken down to finding a reasonable set of force field parameters for the ferredoxin alone, the ROC alone, and for a complex consisting of the Rieske donor, the Asp180 bridging molecule and the ROC acceptor: i.e., a donor/bridge/acceptor (DBA) complex [[1-3](#_ENREF_1)], or more explicitly, proton coupled electron transfer (PCET) where transfer [[4-6](#_ENREF_4)] can occur through a relay of hydrogen bonds [[7](#_ENREF_7),[8](#_ENREF_8)]. The current study is meant to establish justification for this process through consideration of the long range influence on partial charges using the computational approach of quantum chemistry. Such long range interactions have not been explored extensively in this problem prior to this work.

In this Supplement, we expand on the details of generating the spin state of the iron in the quantum chemistry calculations, the development of Amber ff parameters to consider the donor-bridge-acceptor (DBA) complex and how to assemble the structure for MD simulations. The organization of this Supplement is as follows. In Section 2, we briefly discuss the structure of the ROC for various oxygenase structures and the observed process of oxidation of small aromatic molecules. In Section 3, we explain how to set up the quantum chemical calculations for the Rieske structure and for the full complex. Section 4 explains how the force field is constructed including the prepin and frcmod files and how to set up the actual MD simulation where some examples of the files and scripts are shown.

# 2 Structure of the ligands in the non-heme Fe(II) site

The main function of the Fe(II) site is to facilitate the oxidation of polyaromatic hydrocarbons (PAH). The Fe helps facilitate this process because it has the ability to change valence and donate electrons to this process. These PAH species are typically toxic to microorganisms and, therefore, there is evolutionary pressure to devise ways to get rid of such toxic materials in the environment. The dioxygenase is an example of a strategy where the PAH is oxidized and used or excreted from the microorganism. This suggests one possible solution to managing unavoidable toxic waste is to engineer bacteria that can make a living on it.

There are many varieties of mononuclear Fe redox centers, for a review see Refs [[9-11](#_ENREF_9)]. A common ligand binding motif for the iron center consists of two His ligands and one carboxylate ligand (Asp or Glu), where these homologous Rieske oxygenase proteins appear to exclusively incorporate Asp. Therefore, we will call these 2His-1Asp redox-center (2H1D, S1B Figure), where the general classification is 2His-1carboxy non-heme Fe redox centers. The carboxylate-ligand usually binds to the Fe at the Oδ position in a bidentate configuration and the histidine ligands bind to the Fe at the Nε position [[12](#_ENREF_12)].


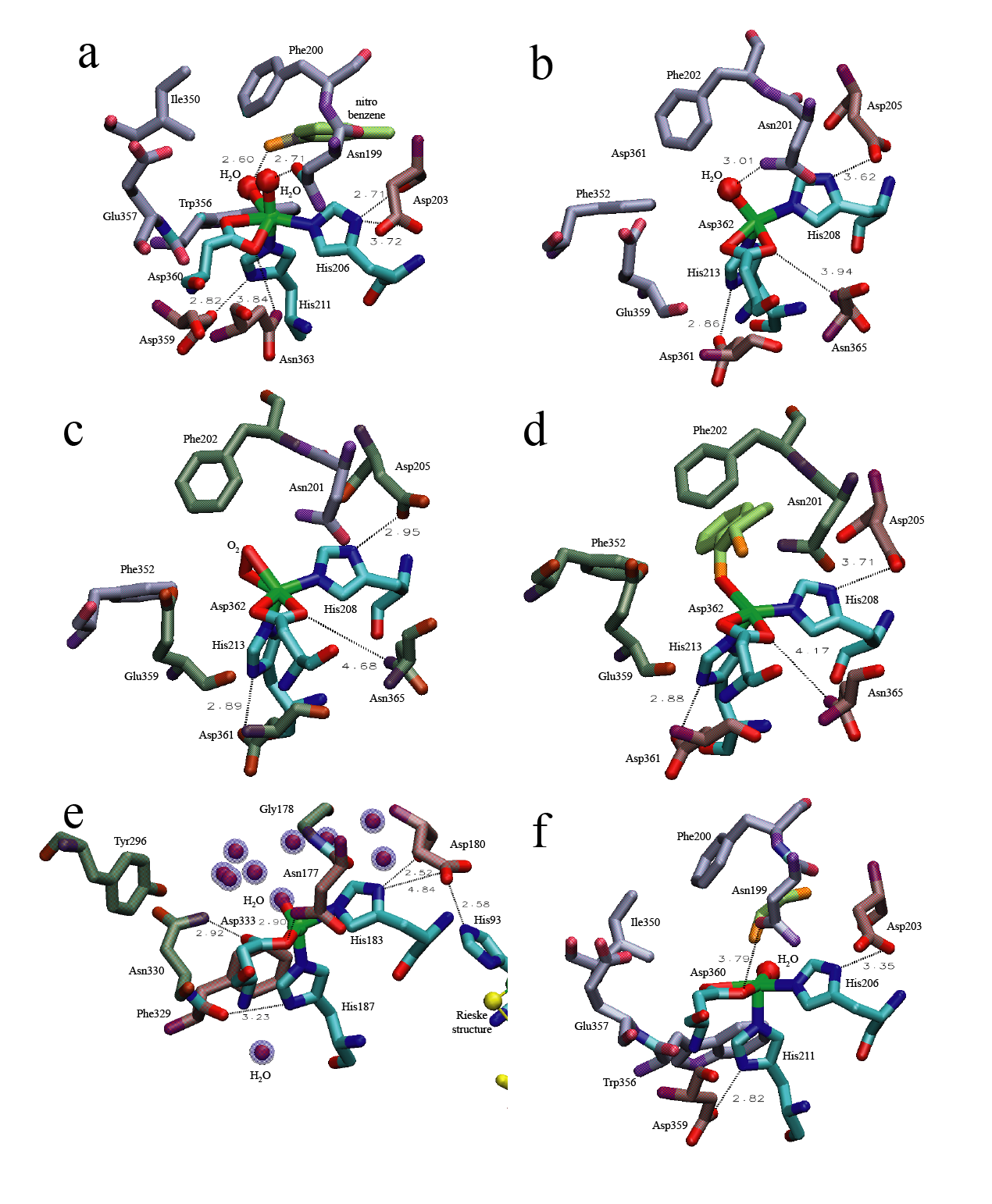


**S1B Figure. Examples of the 2His-1Carboxy redox center in different coordination environments and various ligands.** Included in the Figure are additional residues around the chamber that interact with the ligands bound to the iron. (a) An example of a structure showing an octahedral arrangement of ligands (PDB: 2BMR). Electron transfer between the Rieske and the Fe center occurs primarily through His 206. (b) An example of a structure exhibiting a distorted square pyramid/trigonal bipyramid arrangement of the ligands (PDB: 1O7G). Electron transfer occurs through His 208. (c) The water in panel b is exchanged for an O_2_ ligand (PDB: 1O7M). (d) The same protein with the reacted naphthalene (PDB: 107P). (e) An example of a structure showing a largely tetrahedral arrangement of ligands (PDB: 2DE6). Electron transfer occurs primarily through His 183. Secondary solvent shell interactions involve the protein backbone chain as well as Asp and are indicated by the red-tinted residues: Asn177, Asp180, Phe329 and Asp332. (f) An example of a typical NDO type structure (PDB: 2BMO) where the secondary solvent shell contains two Asp residues (Asp203 and Asp359) that interact directly with the histidine ligands of the non-heme Fe (His206 and His211). Note that only structure in panel e has extensive amounts of water in the chamber and it is the only monodentate (as is the case for (e)). Water appears to be a strong factor in the structure of the 2His-1Carboxy redox center. Color coding: standard colors (white: H, cyan: C, blue: N, red: O, green: Fe). Silver tinted: residues that interact with the bound water ligand and are within 4 Å. Red tinted: negatively charged electrophilic residues that interact with the binding ligand including the universally conserved Asp which is the subject of this study.

The iron is generally in the ferrous Fe(II) state in this complex [[9](#_ENREF_9),[10](#_ENREF_10),[13](#_ENREF_13),[14](#_ENREF_14)]. Precise measurements using magnetic circular dichroism (MCD) [[15](#_ENREF_15)] suggest a coordination number of six ligands in a distorted octahedron (S1B Figure, panel a) or five ligands in a distorted square-planer or trigonal-bipyramid (S1B Figure, panel b) arrangement [[9](#_ENREF_9),[14](#_ENREF_14),[16](#_ENREF_16)]. Studies of the redox reaction have suggested the following sequence of events [[10](#_ENREF_10),[13](#_ENREF_13),[17](#_ENREF_17),[18](#_ENREF_18)]. When the chamber is empty of the substrate or in the inactivated state, the His, Asp and water ligands form in either a five-fold coordination with one water molecule (S1B Figure, panel b) or in a six-fold coordination with two water molecules (S1B Figure, panel a). When a specific PAH enters the chamber, the coordination number typically changes to fivefold (a distorted square pyramid or trigonal-bipyramid arrangement) [[16](#_ENREF_16)]. In the course of the process, O_2_ is thought to displace the water (S1B Figure, panel c) [[16](#_ENREF_16),[19](#_ENREF_19),[20](#_ENREF_20)]. The oxygen reacts with the PAH at a stereo-specific position by accepting electrons from the Fe redox center and the [2Fe-2S] Rieske charge reservoir (S1B Figure, panel d) [[11](#_ENREF_11),[16](#_ENREF_16),[18-25](#_ENREF_18)]. Under these conditions, the Fe is changed to the Fe(III) state. An additional electron is then supplied by a ferredoxin protein that shuttles between Ferredoxin NADP reductase and the oxygenase, whereupon the Fe-center is thought to return to the Fe(II) state and this in turn is thought to permit release of the stereo-specifically oxidized PAH [[13](#_ENREF_13),[17](#_ENREF_17)]. S1B Figure (panel e) shows the monodentate Asp of 2DE6 and, in contrast, S1B Figure (panel f) shows the bidentate Asp of 2BMO with the secondary solvent shell Asp residues (D203 and D359) interacting with the H206 and H211, around the chamber.

# 3 Cluster calculations with iron atoms

## 3.1 Assigning spin structure with Gaussian (using GIFA)

On the isolated Rieske structure and the isolated ROC, the ORCA package [[26](#_ENREF_26)] can be used with the broken symmetry option. Recent versions of the Gaussian 09 package (g09) [[27](#_ENREF_27)] also offer this feature; however, our version did not have it and we needed to use a dedicated machine that only had the Gaussian 03 package (g03) [[28](#_ENREF_28)] (which does not permit calculation using broken symmetry). To optimize these very large clusters, we had to use g03. To achieve the spin structure on the irons, we used an approach called Generalized Ionic Fragment Approach (GIFA) [[29](#_ENREF_29)]. This method was also of value because we wanted to be able to assign a particular configuration of spins. Therefore, we have approached the problem in the same way for both g03 and the Gaussian 09 package (g09) [[27](#_ENREF_27)].

GIFA [[29](#_ENREF_29)] can be downloaded from the following website

<http://chemistry.montana.edu/~szilagyi/GIFA>

The first stage of this approach consists of removing the ligands bound to the Fe and solving them as much smaller isolated structures with a formal charge assigned as though they were ionic species bound to an ionic charge state of Fe. For example, the Cys residue and the Asp residue would bind to the Fe with a charge state of minus one, the S in the [2Fe-2S] ring would bind with a charge state of minus two. Specifically, we built fragments for Cys, Asp and His in the following form. For histidine, we constructed two types of methyl imidazole molecules: one with a δ1 Hydrogen (4-methyl 1,3-diazocyclopenta 1,4-diene) and the other with an ε2 Hydrogen (4-methyl 1,3-diazocyclopenta 2,4-diene). Both of these structures have a neutral charge. For cysteine, we constructed a thiomethyl ion (charge -1). For aspartic acid, we constructed an acetate ion (charge -1). In addition, we had to construct water molecules for the ROC. The initial xyz structure input files used with GIFA are shown in S1 File (Listing 1). Note that there are two kinds of His residue files MeImid14_dH.xyz and MeImid24_dH.xyz, corresponding to these respective structures.

The Fe is then assigned a specified charge and spin state appropriate for the particular state of interest. Each fragment must be optimized separately with its particular charge and spin state. Hence, for the Rieske [2Fe-2S] structure, we solved the His and Cys clusters separately, then the S^2-^ was solved separately, where all of these structures were assigned a spin multiplicity of one. Finally each Fe was solved with the specific spin and charge. When the magnitude of the charge and spin on both Fe ions is the same, only one Fe needs to be solved; however, when the spin and or the charge is different on the Fe ions, then more than one state of Fe must be specified and solved separately. Finally, all these fragments were given an initial optimization at the UHF/TZVP level to generate separate chk files, and these chk files were converted to fchk files. Both the geometry and the electronic structure of these small fragments should be optimized.

In the second step, we built the full ligand structure. This can be done either by some molecular structure builder or from some experimental structure that supplies the coordinates of the molecule. Whatever way the structure of the full cluster is obtained, the next step is to obtain a first guess by running Gaussian with Guess(Only, Save) in the route. This produces the Hückel guess that will be used as a “scaffold” for merging the fully optimized ionic fragments. The guess file must be generated using the same theory level as the initial fragments, and in constructing the molecule, the order of the atoms is crucial, so they must correspond exactly to the order of the atoms in the individual ionic fragments above. The tedious step is making sure that the fragments and the cluster have the same order of atoms with the corresponding internal coordinates. When this is done correctly, the guess scaffolding can be built.

In the next step, a file called mergelist must be constructed. For example, the Rieske in the rest state would require a file of the following format (for g03):

> cat mergelist

aFe3+.fchk

bFe3+.fchk

aS2-.fchk

aS2-.fchk

aSMet-.fchk

aHis_db.fchk

aSMet-.fchk

aHis_db.fchk

bFe3+.fchk

aFe3+.fchk

bS2-.fchk

bS2-.fchk

bSMet-.fchk

bHis_db.fchk

bSMet-.fchk

bHis_db.fchk

Rieske_oxidized.fchk

where the “a” and “b” prefix specifies the spin up, spin down state of the structure. Since the Fe(III) is antiferromagnetic (AF), this AF structure is accomplished by specifying one of the irons as alpha (spin up) and the other as beta (spin down). The His is bound at the delta position, and the mergelist file indicates that the initial guess file was Rieske_oxidized.fchk.

To set up g09 in the same way, the syntax of the input file is simpler

> cat mergelist

aFe3+.fchk

bFe3+.fchk

aS2-.fchk

aS2-.fchk

aSMet-.fchk

aHis_db.fchk

aSMet-.fchk

aHis_db.fchk

Rieske_oxidized.fchk

where the newer version of GIFA only requires listing the first set and automatically assigns the second set. The program mergeWFN.exe (available at the above mentioned web site) is then used to construct a file merged.fchk from the above scaffold file Rieske_oxidized.fchk.

From here, the first step is to solve the structure ionically at the same UHF/TZVP level of theory. This reconstructed merge file provides a “better” guess, so this step is very important. From this step, one gradually introduces covalent mixing using more advanced theory. The details of this process will be discussed in the various Sections that follow.

The GIFA method was used in this work to achieve the antiferromagnetic (AF) coupling in the Rieske structure and also to couple the total spin of the Rieske with the Fe^2+^ on the ROC. This method was particularly useful on the very large molecules such as the one describe here because it was possible to control the arrangement of the spins on the various Fe atoms.

The structural files and mergefile lists for building the large Rieske-Asp-ROC complex are shown in Listings 1 and 2 in S1 File, in the pages that follow. The strategy for building the files will be explained in Section 3.2.3.

## S1 Listing 1: Coordinates for structural fragments used to build Rieske and ROC for quantum chemistry calculations

### Iron

> cat Fe.xyz

1

Fe 0.000000 0.000000 0.000000

### Sulfur

> cat S.xyz

1

S 0.000000 0.000000 0.000000

### 4-methyl 1,3-diazocyclopenta 1,4-diene (for His with δ1 hydrogen)

> cat MeImid14_dH.xyz

12

scf done: -263.912584

N -0.035230 -0.001227 0.010479

C -0.018356 -0.000591 1.294660

N 1.242387 0.006846 1.787054

C 2.098943 0.010690 0.710160

C 1.286422 0.006046 -0.369456

H -0.879759 -0.005075 1.931409

H 1.496737 0.008238 2.744742

H 1.568026 0.007191 -1.402245

C 3.584970 0.019215 0.854138

H 4.049410 0.017904 -0.123278

H 3.931290 0.901134 1.384795

H 3.940730 -0.854809 1.391564

### 4-methyl 2,4-diazocyclopenta 1,4-diene (for His with ε2 hydrogen)

MeImid24_eH.xyz

12

scf done: -263.913062

N 0.008733 -0.008851 -0.001536

C 0.018754 -0.002880 1.286259

N 1.268969 -0.001807 1.785282

C 2.122510 -0.007992 0.704687

C 1.328861 -0.012070 -0.389229

H -0.848684 0.001100 1.914994

H 1.522002 0.000801 2.742613

H 3.185384 -0.008331 0.817634

C 1.713576 -0.019299 -1.833383

H 2.791391 -0.016682 -1.950549

H 1.319138 -0.898290 -2.331383

H 1.313680 0.851365 -2.341507

### thiomethyl anion (for Cys)

> cat SMe-.xyz

5

S 0.000000 0.000000 0.000000

C 0.000000 0.000000 1.840236

H 1.038973 0.000000 2.178621

H -0.501173 0.886709 2.228408

H -0.504121 -0.885855 2.228733

### acetate anion (for Asp)

> cat Acetate-.xyz

7

O 0.000000 0.000000 0.000000

C 0.000000 0.000000 1.262043

O 1.042311 0.000000 1.972579

C -1.348574 -0.016854 1.977166

H -1.229728 0.090139 3.055333

H -1.984538 0.786830 1.596747

H -1.862002 -0.960104 1.765864

### water

> cat water.xyz

3

O 0.000000 0.000000 0.000000

H 0.000000 0.000000 0.990870

H 0.941515 0.000000 -0.306607

## S1 Listing 2: g09 mergelist using GIFA for complex

### Oxidized structure $\text{\{[Fe}^{3+}(s=5/2)\text{Cys},\text{Fe}^{3+}(s=-5/2)\text{His}]^{0}\oplus\text{Asp}^{1-}\oplus[\text{Fe}^{2+}(s=4/2)]\}^{Q=0,S=5}$

> cat mergelist

aFe3+.fchk

bFe3+.fchk

aS2-.fchk

aS2-.fchk

aSMe-.fchk

aSMe-.fchk

aMeImi.fchk

aMeImi.fchk

aacetate-.fchk

aMeImi.fchk

aMeImi.fchk

aFe2+.fchk

aacetate-.fchk

awater.fchk

awater.fchk

awater.fchk

awater.fchk

awater.fchk

Rieske-FeHis2AcW_qp0S5.fchk

### **Reduced (maximum spin) structure**

$$\text{\{[Fe}^{3+}(s=5/2)\text{Cys},\text{Fe}^{2+}(s=-4/2)\text{His}]^{1-}\oplus\text{Asp}^{1-}\oplus[\text{Fe}^{2+}(s=4/2)]\}^{Q=-1,S=6}$$

> cat mergelist

aFe3+.fchk

bFe2+.fchk

aS2-.fchk

aS2-.fchk

aSMe-.fchk

aSMe-.fchk

aMeImi.fchk

aMeImi.fchk

aacetate-.fchk

aFe2+.fchk

aMeImi.fchk

aMeImi.fchk

aacetate-.fchk

awater.fchk

awater.fchk

awater.fchk

awater.fchk

awater.fchk

Rieske-FeHis2AcW_qm1S6.fchk

### Reduced (minimum spin) structure

$$\text{\{[Fe}^{3+}(s=-5/2)\text{Cys},\text{Fe}^{2+}(s=4/2)\text{His}]^{1-}\oplus\text{Asp}^{1-}\oplus[\text{Fe}^{2+}(s=4/2)]\}^{Q=-1,S=4}$$

> cat mergelist

bFe3+.fchk

aFe2+.fchk

aS2-.fchk

aS2-.fchk

aSMe-.fchk

aSMe-.fchk

aMeImi.fchk

aMeImi.fchk

aacetate-.fchk

aFe2+.fchk

aMeImi.fchk

aMeImi.fchk

aacetate-.fchk

awater.fchk

awater.fchk

awater.fchk

awater.fchk

awater.fchk

Rieske-FeHis2AcW_qm1S4.fchk

## 3.2 Specific quantum chemistry methods

Simplified ligands were built by truncating the amino-acid side-chain region from the beta carbon (Cβ) of the respective amino acid. In his way, a methyl imidazole molecule was used for the His residues, acetic acid for the Asp residues and a thiomethane anion was used for the Cys residues. In assembling the full structure of these truncated side chains into a full complex, the order of the atoms of each substructure was maintained in order to apply the GIFA method [[29](#_ENREF_29)] with the desired initial AF coupling between the Rieske and the ROC Fe ions.

The smaller structures were optimized at the BP86/6-311+G(d) level [[30-33](#_ENREF_30)], based upon suggestion from Ref [[29](#_ENREF_29)] and computational economy. The final electronic structure of the DBA-complex was first only optimized at the OPBE/6-311+G(d) level [[34](#_ENREF_34)] and final refinement was done using OPBE/cc-pVTZ. Most of the results reported in this study are from OPBE, through relatively good results can also be obtained using BP86 as recommended. Overall, we eventually chose OPBE over BP86 because it did appear to make slightly better predictions and it was also a bit more stable in optimizing very large complexes.

### 3.2.1 The Rieske structure

The His was modeled with a methyl imidazole (H bound to the respective delta N atom) and Cys with methyl thiolate. GIFA was used to restructure the spin environment of the Fe on the Rieske to the oxidized configuration Fe^3+^(*s*=+5/2):Fe^3+^(*s* = –5/2) or the reduced configuration Fe^3+^(*s* = +5/2):Fe^2+^(*s* = –4/2). The new spin structure was again optimized with this spin structure. The oxidized and reduced structures were optimized at the BP86/6-311+G(d) level and OPBE/cc-pVTZ using g09. Partial charges were obtain using the RESP procedure and CHelPG [[35](#_ENREF_35),[36](#_ENREF_36)] (CHarges from Electrostatic Potentials using a Grid based method).

First, an initial structure of the Rieske was constructed using the molecular builder Molden 4.8 [[37](#_ENREF_37)]. An example of such a structure is shown in S1A Figure (panel a). The fragments for methyl imidazole, thiomethyl ion, S^2-^ and Fe were constructed and optimized at the UHF/TZVP level [[38](#_ENREF_38),[39](#_ENREF_39)]. The full structure was constructed such that the same order of the atoms as the fragments was maintained. From this initial full structure, an initial guess was obtained and saved. The wave function of the different fragments (g03: formchk) could then be merged with the rest of the information in the fchk file using the GIFA application. The arrangement of the fragments and their spins was specified in a mergelist (as mentioned above). By rigorously following the order, it is possible to specify an exact spin structure: as though the Fe^2+^/ Fe^3+^ ions were isolated with no wave function overlap. From here, an SCF calculation was done at the UHF/TZVP level, followed by an SCF calculation at the UBHandHLYP/TZVP [[40](#_ENREF_40)] level to begin to bring a more mixing of the electron density. This is followed by an SCF calculation at the BP86/TZVP level independently OPBE/TZVP level. From this stage, optimizations were done at the BP86/6-311+G(d) level or the OPBE/6-311+G(d) level or the OPBE/cc-pVTZ level. Although these other levels were tested, we report only calculations done at the OPBE/cc-pVTZ level.

Another way to construct the fragments is to take them directly from the PDB structure and organize them in xyz files. Examples of the fragment files are shown in Listing 1 in S1 File.

### 3.2.2 The non-Heme Fe-center structure

Similar to the Rieske structure, the H183 and H187 were modeled using a methyl imidazole (H bound to the respective epsilon N atom), acetic acid substituted for the Asp residue, and one bound water ligand. The 4-fold coordination of the two His ligands, the monodentate Asp ligand, the single water molecule were bound to the Fe-center were set up in such a way that four additional water molecules could be added to the structure to produce an optimizable water configuration for four free water molecules around the unbound Oδ of the acetate carboxyl and the O of the bound water. The structures were optimized to yield reproducible results on multiple trials. The various spin states for the Fe were all optimized at the BP86/6-311+G(d) level [[30](#_ENREF_30),[31](#_ENREF_31)]. and subsequently redone with optimization at the OPBE/cc-pVTZ level.

To ensure that the results were not basis set or density function dependent, other levels of theory were also tested: B3LYP/TZVP [[39](#_ENREF_39)], B3LYP /6-311+G(d), BP86/6-311++G(d), BP86/6-311+G(3df,3pd), BP86/cc-pVTZ, BP86/AUG-cc-pVTZ as well as the relativistic version of cc-pVTZ [[33](#_ENREF_33)]. Only minor changes occurred. Nevertheless, based on the overall expected symmetry of some of the outcomes, the best configurations were generated by OPBE/cc-pVTZ level theories.

### 3.2.3 The full complex: Rieske, Asp180 and non-Heme Fe-center

The Rieske structure, Asp (AcO^-^) bridging ligand and the non-Heme Fe center were assembled by combining the separate units together and arranging them with the aid of the molecular editor Molden (v 4.8) [[37](#_ENREF_37)]. The structure was then optimized at the B3LYP/6-311+G(d) level applying a distance constraint between the non-Heme Fe center and the Rieske structure. Specifically in terms of the structures discussed in the last two subsections (see also Figs 2b and 2c, main text), two distance constraints were applied: one between Fe(2) and Fe(46) and one between N(29) and C(55): see coordinates in Listings 3 and 4 in S1 File for the oxidized and reduced states, respectively. The remaining coordinates were free to vary in the optimization process.

#### *3.2.3.1 Initial optimization of the Rieske-Asp-ROC complex*

To help settle the structure into a stable configuration, a loose optimization was done at the B3LYP/6-311+G(d) level for charge (*q*) and multiplicity (S) such that $q=0$ and $S=5$. This corresponds to $\{\text{Fe}^{3+}(\text{Cys}),\text{Fe}^{3+}(\text{His})\}^{q=0,S=1}$ on the Rieske with two ligands of 4-methyl imidazole of zero charge and two ligands of thiomethyl (charge $-1$), the non-heme iron (Fe^2+^) with , two acetate molecules (charge $-1$), and 5 waters of (charge 0). The sum is therefore 0. In the early stage, the B3LYP was used because it maintained a better tetrahedral configuration around the ROC. This initial step allowed partial optimization of the bond lengths so that the SCF calculations could be done. However, without the aid of the GIFA application, the g03 program cannot directly compute the proper AF spin symmetry around the [2Fe-2S] structure [[41-43](#_ENREF_41)].

#### *3.2.3.2 Using GIFA to introduce spin states*

The GIFA program was used to reconfigure these spins and to build multiple spin states. For the oxidized Rieske structure in the DBA-complex, the non-Heme Fe-center has a spin state *s*=4/2 and the Rieske has an antiferromagnetically coupled spin state Fe^3+^(Cys, *s*=+5/2):Fe^3+^(His, *s*= –5/2). The total multiplicity of the complex is $S=5$ due to the high spin ferrous state of the non-heme iron. The reduced Rieske introduces two possibilities: low spin and high spin. The low spin state has Rieske Fe^3+^(Cys, *s* = –5/2):Fe^2+^(His, *s* = +4/2) and non-Heme Fe^2+^(*s* = +4/2) with corresponding multiplicity *S*=4. The high spin state has Rieske Fe^3+^(Cys, *s* = +5/2):Fe^2+^(His, *s* = –4/2) and non-Heme Fe^2+^(*s* = +4/2) with corresponding multiplicity $S=6$.

After building the rough-optimization structure, all the individual fragments constructed (see 3.1) were arranged in a mergelist file that mapped the fragments to the full structure with the desired spin configuration. The fragments were then merged with the coordinates for the full structure based on their respective fchk files. This merged checkpoint file was used to carry out further calculations. To introduce covalent effects into the electronic structures, a single point SCF calculation was carried out at the UHF/TZVP level. This was followed by a SCF calculation at the UBHandHLYP/TZVP level, followed by OPBE/TZVP to gradually introduce covalency to the molecular orbitals (MOs). From this stage, full optimization with DFT was introduced. The structure was optimized at the OPBE/6-311+G(d) level. In optimizing the structure, OPBE was the main density functional approach; however, B3LYP and BP86 were attempted independently in the same way.

Optimizations were done in vacuo. After completing these *in vacuo* optimizations, the optimized structures were then computed at full convergence at the OPBE/6-311+G(d) level with an implicit solvent environment around the structures [[44](#_ENREF_44),[45](#_ENREF_45)]. This was tried for various reaction field solvents: using g03 (with Radii=uff , OFac=0.8, Rmin=0.5) water, benzene and ethanol (EPS=10.0), and using g09 n-hexane (with default settings). We find that n-hexane in g09 yields a good reaction field: better than benzene, ethanol or water. When using reaction field calculations, the results suggest strongly that n-hexane yields the most credible results.

In the final step, the partial charges were calculated using CHelPG [[35](#_ENREF_35),[36](#_ENREF_36)] and also with RESP. Methods for obtaining RESP charges are explained in Section 4. Results for the n-hexane are similar to the *in vacuo* results for the partial charges.

Listing 2 in S1 File shows examples of the GIFA file needed for modifying the Rieske-Asp-ROC complex using g09. The g03 setup requires the reversed spins listing in addition to the first.

A full geometry optimization (*in vacuo*) was carried out with a minimal number of restraints: the iron on the His side of the Rieske to the ROC Fe^2+^ and N(ε2) on the Rieske side to the ROC C(ε1). The initial attempt at optimization with the oxidized structure required more than 9 months (wall clock) with 8 processors. With this initial structure, further geometry optimization with GIFA (also *in vacuo*) was carried out for both the oxidized and the reduced (high spin) states also required a wall clock time of about 9 months. A single-point calculation with implicit solvent can sometimes succeed in approximately one day. Optimizing the structure was used to ensure that the difference between electronic states was minimized. Whereas some reactions may occur before the structure has a chance to settle, the reduced and oxidized states of the Rieske are known to be stable for extended periods of time. Hence, these stable states should be optimized separately.

The coordinates of the resulting optimizations are shown in Section 3.3: Listing 3 (oxidized complex) and Listing 4 (reduced complex) in S1 File. After achieving geometry optimization using g03, a full SCF optimization was carried out on all these structures using g09. All resulting partial charges reported in this work are based on these g09 SCF calculations using CHelPG.

In the reduced complex, the energy difference between the lower multiplicity complex (S=4) and the high multiplicity complex (S=6) was about 0.75 kcal/mol in favor of the high spin state. Since the spin state on the Rieske structure nicely mirrors the change in the multiplicity, it suggests that the coupling with the Fe^2+^ spin on the ROC is weakly favored toward ferromagnetic. Nevertheless, the difference is small and close to that of thermal energy at ambient temperatures.

### 3.3 Coordinates obtained from geometry optimization using g03 and GIFA

In the next two pages, the coordinates (in xyz format) are shown for the geometry optimization of the oxidized and reduced structure using g03 combined with the features of GIFA: Listing 3 and 4 in S1 File, respectively.

These files were used subsequently to do SCF calculations with g09 for other types of complexes, with the assumption that the electronic state for all other conditions is short-lived compared to the physical arrangement of the atoms (the Born-Oppenheimer approximation).

### S1 Listing 3: Optimized ROC-Asp-Rieske complex (oxidized) using g03/GIFA

$$\text{\{[Fe}^{3+}(s=5/2)\text{Cys},\text{Fe}^{3+}(s=-5/2)\text{His}]^{0}\oplus\text{Asp}^{1-}\oplus[\text{Fe}^{2+}(s=4/2)]\}^{Q=0,S=5}$$

> cat complex_qp0S5.xyz

92

Fe -6.147715 2.186448 -0.322407

Fe -5.922999 -0.476251 -0.165630

S -6.214701 0.714159 -1.964207

S -5.918055 0.944248 1.489813

S -8.104299 3.367795 -0.363322

C -8.019636 4.501490 1.056782

H -8.903740 5.149754 1.036805

H -8.014261 3.948691 2.002564

H -7.122565 5.127357 1.007415

S -4.313885 3.553155 -0.436680

C -4.457487 4.441132 -2.017671

H -3.638520 5.167065 -2.085352

H -4.382075 3.750441 -2.864624

H -5.409659 4.978065 -2.082551

N -7.309499 -2.009339 0.203376

C -6.968162 -3.080375 0.920968

N -8.028314 -3.882464 1.095101

C -9.107959 -3.313338 0.470331

C -8.661346 -2.138392 -0.088538

H -5.986695 -3.295478 1.322560

H -8.025794 -4.753058 1.607295

H -10.083999 -3.780764 0.476253

C -9.441326 -1.146864 -0.874239

H -10.488557 -1.458912 -0.938146

H -9.410468 -0.151872 -0.415783

H -9.054810 -1.045002 -1.895075

N -4.100621 -1.493348 -0.219979

C -3.199061 -1.436307 0.766159

N -2.151775 -2.224265 0.501518

C -2.380761 -2.833084 -0.705286

C -3.593293 -2.376413 -1.164522

H -3.287652 -0.835459 1.661729

H -1.360126 -2.408735 1.135177

H -1.676520 -3.533622 -1.135804

C -4.285948 -2.723373 -2.434625

H -3.691308 -3.446025 -3.002702

H -5.271943 -3.170327 -2.256046

H -4.436571 -1.840349 -3.066962

O 0.051887 -2.778010 2.097762

C 0.892514 -3.219748 2.926801

O 2.129375 -2.937903 2.917266

C 0.390928 -4.164538 4.017754

H 1.171581 -4.411108 4.744075

H -0.461779 -3.719598 4.545526

H 0.031799 -5.096969 3.561991

Fe 6.961163 0.133419 -0.353812

N 4.999433 -0.322890 0.107123

C 4.564252 -1.136807 1.071912

N 3.231909 -1.277670 1.018651

C 2.754207 -0.520645 -0.028322

C 3.868570 0.063874 -0.585390

H 5.184783 -1.623499 1.813742

H 2.680020 -1.871258 1.691500

H 3.925278 0.735243 -1.434422

C 1.314227 -0.439252 -0.393424

H 0.910955 -1.424197 -0.649313

H 0.708963 -0.040569 0.428677

H 1.185934 0.218360 -1.259092

N 7.689437 -1.316133 -1.658245

C 8.960918 -1.555721 -1.968816

N 9.037572 -2.547185 -2.875869

C 7.766098 -2.986770 -3.178327

C 6.941301 -2.202508 -2.406449

H 9.828420 -1.049634 -1.564805

H 9.897782 -2.911825 -3.261072

H 5.860602 -2.229053 -2.341435

C 7.491776 -4.090595 -4.133767

H 6.416223 -4.279433 -4.193114

H 7.848621 -3.851688 -5.144078

H 7.980754 -5.022008 -3.820549

O 6.879487 1.901809 -1.247053

C 6.858652 3.091260 -0.744429

O 6.873272 3.349562 0.478364

C 6.798300 4.226109 -1.745781

H 7.521847 5.004890 -1.479154

H 5.802696 4.686586 -1.698496

H 6.978856 3.889483 -2.769774

O 8.237635 -0.027365 1.237737

H 7.851711 -0.041466 2.154745

H 9.052317 0.534131 1.282350

O 7.049575 0.097433 3.632352

H 6.644331 0.991845 3.587829

H 7.622649 0.092249 4.406247

O 10.410635 1.555602 1.511359

H 11.180372 1.472460 0.939578

H 10.171090 2.507534 1.521432

O 5.893985 2.570196 3.140990

H 6.165014 2.809394 2.237822

H 4.931667 2.598236 3.149650

O 9.505369 4.195076 1.484190

H 8.578365 4.106902 1.205831

H 9.490443 4.666549 2.323734

### S1 Listing 4: Optimized ROC-Asp-Rieske complex (reduced) using g03/GIFA

$$\text{\{[Fe}^{3+}(s=5/2)\text{Cys},\text{Fe}^{2+}(s=-4/2)\text{His}]^{1-}\oplus\text{Asp}^{1-}\oplus[\text{Fe}^{2+}(s=4/2)]\}^{Q=-1,S=6}$$

> cat complex_qm1S6.xyz

92

Fe 6.502396 2.181390 0.065701

Fe 5.869226 -0.369271 0.284417

S 6.473097 0.955953 1.911718

S 5.859697 0.817927 -1.552507

S 8.661548 3.002285 -0.226343

C 8.678440 3.656444 -1.919862

H 9.657618 4.111231 -2.123202

H 8.502613 2.858773 -2.651284

H 7.902881 4.420236 -2.051024

S 5.056736 3.996149 0.124857

C 5.506870 4.889020 1.639786

H 4.907088 5.806680 1.710343

H 5.314147 4.275756 2.528212

H 6.568556 5.162492 1.633022

N 7.081772 -2.131335 0.025167

C 6.644608 -3.173433 -0.667008

N 7.662544 -4.008109 -0.980119

C 8.816980 -3.472535 -0.456845

C 8.450184 -2.301919 0.168103

H 5.617441 -3.349865 -0.958754

H 7.589291 -4.849716 -1.530367

H 9.779230 -3.954984 -0.578609

C 9.319813 -1.326549 0.874760

H 10.367030 -1.649635 0.840204

H 9.242525 -0.331059 0.423402

H 9.022940 -1.212904 1.923883

N 4.061200 -1.462984 0.453415

C 3.137212 -1.536277 -0.505651

N 2.085113 -2.285378 -0.120459

C 2.342798 -2.723046 1.150968

C 3.569567 -2.207379 1.512100

H 3.206666 -1.039259 -1.465410

H 1.277106 -2.562104 -0.696827

H 1.640402 -3.349226 1.688159

C 4.292953 -2.356475 2.804681

H 3.703653 -2.957209 3.507720

H 5.267277 -2.845161 2.673172

H 4.489395 -1.379992 3.263650

O -0.165862 -3.144351 -1.525266

C -1.027417 -3.758770 -2.201433

O -2.274385 -3.517209 -2.205579

C -0.544388 -4.885936 -3.118520

H -1.376772 -5.464904 -3.531738

H 0.028299 -4.453548 -3.950560

H 0.137579 -5.553654 -2.577191

Fe -7.018177 0.199031 0.248840

N -5.083539 -0.383068 -0.139652

C -4.652904 -1.427750 -0.855827

N -3.317002 -1.545934 -0.811808

C -2.827687 -0.526163 -0.029898

C -3.936699 0.184616 0.379676

H -5.283951 -2.101661 -1.423247

H -2.771450 -2.325294 -1.336981

H -3.978047 1.070107 1.004824

C -1.381116 -0.325089 0.257721

H -0.793696 -0.219667 -0.660759

H -1.238829 0.579281 0.858951

H -0.956831 -1.171609 0.808482

N -7.625849 -0.562073 2.087856

C -8.242644 -1.713304 2.326454

N -8.373782 -1.912650 3.661746

C -7.816806 -0.840978 4.327151

C -7.362658 -0.015359 3.326119

H -8.593590 -2.410073 1.575330

H -8.800700 -2.720589 4.092951

H -6.862702 0.941632 3.420416

C -7.775877 -0.730243 5.807968

H -7.292757 0.207884 6.098683

H -8.781056 -0.735452 6.252958

H -7.204738 -1.549460 6.267275

O -6.980216 2.220103 0.460173

C -7.177353 3.136056 -0.403685

O -7.446228 2.943063 -1.621026

C -7.055290 4.567037 0.089159

H -7.856283 5.187710 -0.327822

H -6.106756 4.985699 -0.273602

H -7.064419 4.627247 1.181082

O -8.445998 -0.743905 -0.990899

H -8.141397 -0.938404 -1.909906

H -9.310249 -0.286032 -1.084781

O -7.480491 -1.062476 -3.501660

H -7.033172 -0.200638 -3.675077

H -8.030612 -1.254531 -4.265150

O -10.847393 0.578758 -1.415442

H -11.460143 0.741589 -0.692723

H -10.643092 1.458553 -1.807400

O -6.333301 1.405311 -3.721615

H -6.664697 1.936909 -2.969484

H -5.373367 1.422964 -3.661989

O -10.092014 3.015711 -2.464759

H -9.147606 3.092011 -2.207902

H -10.119261 3.141861 -3.417396

# 4 Constructing and benchmarking the force field

The reasoning on partial charge development used here is that, since the Amber99ffSB has already been optimized to a large extent for the standard amino acid residues and their interactions with water, whatever force field we generate should meld closely with it. If for no other reason, having similar conditions yielding similar results renders it possible to benchmark the results to some standard. Since these parameters are intended to be used in the Amber force field calculations, it makes good sense to compare the results with these parameters.

As a footnote, in Amber 99ffSB, the partial charges are the same as Amber ff94 for proteins, but there are some differences in the rest of the force field. We only claim these parameters to be a *de facto* standard in the MD simulation business, much the same as B3LYP is the *de facto* standard for quantum chemistry calculations. We therefore call these parameters the “Amber benchmarks”.

The most efficient way to accomplish this is use the amino acids in the existing Amber99ffSB prepin information (Amber 94 partial charges) as a template to map the partial charges of the respective atoms of the corresponding ligand to a new prepin file of the same form but with different names for specialized interactions. The prepin files given in Listings 5 and 6 (S1 File) correspond to this strategy, where the name of the residue and the partial charge are different; however, as much as possible, the rest remains largely unchanged (unless it actually is different).

Second, we expect that the partial charges will have some similarity with the corresponding Amber benchmarks except near the interface between the Fe and the respective ligand and near the terminators (Cβ, Hβ). As one moves further away (for example the epsilon atom at the Fe-interface), there should be a closer match to the isolated residue case. Hence, one can build the force field progressively by adding more of the structure onto the optimized results and progressively optimizing these larger and larger complexes.

To improve the correlation between the partial charges in the Amber benchmarks and the newly constructed residues, the truncated structures with terminal Cβs were extended to the full structure. For example, S1A Figure shows the truncated (left) and extended (right) Rieske (S1A Figure, panel a) and ROC (S1A Figure, panel b) structures. Since the Amber benchmarks were built with considerable testing, some reasonable similarity was expected for the residues that are present in similar default chemical environments as that for which the Amber benchmarks were intended to model. Hence, partial charges from the Cβ truncated structure, from the extended structure and from the Amber benchmarks are compared side by side (see S3 File [Excel Tables]).

Following the strategy described in Wiener et al [[46](#_ENREF_46),[47](#_ENREF_47)], we built up the partial charge in the following manner. The region around Cβ of the Cβ-truncated structure (S1A Figure, panels a and b “truncated”) typically had very different partial charges than the extended structure (S1A Figure, panels a and b “extended”) or the Amber benchmarks. In the extended structures, the C, O, and N-H (forming the amide of the principal chain) exhibited similar (±10%) partial charges as the corresponding partial charges found in the Amber benchmarks (prepin files). The extended structure often shared a relatively similar correspondence with the Amber benchmarks at the Cβ/Hβ atoms. However, no matter what we tried, the Cα/Hα atoms appear to be noticeably different in the extended structures compared to the Amber benchmarks. This was true even with the RESP charges. Just like our results, Ref [[48](#_ENREF_48)] also finds a large positive charge in the Cα/Hα location even using UHF/6-31G* (the level of theory recommended by the Amber developers [[49](#_ENREF_49),[50](#_ENREF_50)]). In fact, those values are even more positive than our own. The reason is not clear. The extended structure and the Cβ-truncated structure tended to have similar partial charges (±5%) over the interface region between the ligand and the respective Fe ion with the exception of the Cα. As a result, we favored partial charges from the extended calculation at the interface between Cα and Cβ. The truncated partial charges (S1A Figure, “truncated”) were used in the partial charge assignment starting from the Cγ position (and higher positions: δ, ε, ζ, etc.); the principal chain along with Cβ (i.e., all N, H, C, O, Cα, Cβ, and Hβ atoms) were replaced with values obtained from the extended structure calculations (S1A Figure “extended”).

## 4.1 Calculation of partial charges (prepin files)

Partial charges were obtained from the resulting g03 optimizations (and later recalculated using g09) using RESP [[50](#_ENREF_50),[51](#_ENREF_51)] (Restrained Electrostatic Potential). For obtaining RESP charges, the command “Pop=(MK,ReadRadii) iop(6/33=2, 6/41=4, 6/42=4)” was included in the route information of the g03/g09 input file. For the Fe, the radius of the Fe was based on data from the heme iron: 1.1 Å. In addition, partial charges were obtained using CHelPG [[35](#_ENREF_35),[36](#_ENREF_36)] (CHarges from Electrostatic Potentials using a Grid based method).

Frequency calculations were all done using g09 using default settings with additional request for Raman information. Early calculations used g03. The results were similar.

### 4.1.1 Rieske structure and the corresponding ligands:

Optimization of the Rieske [2Fe-2S] in various states with the specific ligands was evaluated for the RESP charges and processed directly using Antechamber, in the Amber Tools package. This was also evaluated using CHelpG. The resulting partial charges were similar in both cases. However, the RESP method combined with the density functional methods and the 6-311 basis set appears to have a tendency to underestimate the charge on the iron. It therefore is still better in our opinion to use CHelpG with these calculations.

### 4.1.2 Non-Heme Fe center:

Because the Asp residue on the Fe center also interacts with several free water molecules in the nearest neighbor interactions, the partial charges are influenced by the local environment and this condition is not likely to be exactly the same as the isolated fragment of the non-heme-Fe-center. The 2DE5-7 structures all exhibit a less common tetrahedral configuration of ligands: two His bonds at the Nε position, a monodentate bond Asp and a single water molecule bond to the Fe. The far more commonly encountered structure consists of a bidentate Asp: resulting in a trigonal bipyramid or octahedral structure (depending on how many water molecules coordinate with the Fe).

Unlike the [2Fe-2S] structure, Antechamber is not designed to handle this complex coordination of water around the Fe center and cannot be configured to do so. Even though the 2DE5-type structure has tetrahedral coordination around the Fe in this case, there was no proper way to specify the configuration of the chains such that Antechamber could find this coordination directly in the initial setup steps within Antechamber. This meant that the various modules of the Antechamber had to be run separately and the files had to be configured and manipulated manually. The bond configurations (label: BOND) in the ANTECHAMBER_AC.AC0 output file had to be modified to inform other applications that comprise the Antechamber suite how to process the structure. Successive operations on the remaining files eventually yielded a prepin file.

The assignment for the connectivity of the water molecules around the Fe center (using BOND in ANTECHAMBER_AC.AC0) showed particularly strong configuration dependence in the predicted partial charges using ESP [[52](#_ENREF_52)]. In particular, because of the weak hydrogen bonding and dipole interactions in this system, to obtain partial charges that resemble those expected of water, it was important to have a good specification of the connectivity of the H-bonded water molecules. The best results came from specifying BOND to the network of Oxygen atoms on the water molecules surrounding the H_2_O ligand. A correct connectivity was defined as one that yielded partial charge assignments that resemble the partial charges of the standard TIP3P water molecule typically used in many Amber based MD simulations.

Because of the subjective nature of the bond assignment using Antechamber (though amenable to rules of thumb), we investigated some alternative methods that would yield good values without having to depend on the selection of the bond arrangement in Antechamber applications. After some testing, we found that CHelPG [[35](#_ENREF_35),[36](#_ENREF_36)] in the Gaussian package yields partial charge values that matched fairly closely with the expected partial charge of TIP3P water and were similar to the best choice of H-bond connectivity used in the manually modified Antechamber files with the manual RESP calculations. After assigning the partial charges using CHelPG, the symmetric locations such as Cβ hydrogen in the amino acid were averaged in similar spirit to the RESP approach. NBA [[53](#_ENREF_53)] was also attempted; however, comparisons of the partial charges suggested that CHelpG was more appropriate for this problem. Therefore, for these problems, we relied exclusively on CHelpG.

Since CHelpG yielded similar partial charges as RESP1, the combined Rieske-Asp-ROC (the Rieske donor, an Asp bridge residue, and the non-Heme Fe center structure along with the local explicit water molecules) were evaluated using CHelPG.

## 4.2. Results of partial charge calculations for oxidized and reduced complex of Rieske-Asp-non-heme Fe center

Up to this point, we have described matters in terms of generalities. Here we discuss how the prepin files were actually built. The prepin files themselves are shown for the oxidized and reduced cases in Listing 5 and 6 in S1 File, respectively. These files are provided in S4 File as a zipped folder. The general contents of the prepin file are built from traditional His, Asp, and Cys residues and the partial charges added to the respective residue based upon the g09 optimization and CHelPG determination.

The first step was to divide the structures into pieces that can be assembled together from the contents in the PDB file. Therefore, the Rieske structure is disassembled into the [2Fe-2S] unit and its ligands. Likewise the ROC is disassembled into the iron and the ligands.

For the Rieske structure, the [2Fe-2S] structure became (FES) and three different ligands were constructed: CYR corresponding to the Cys (Cys69 and Cys90), HR1 (His71) and HR2 (His93). These residues have force field parameters that permit binding them to the [2Fe-2S] (FES). Hence, two unique His residues were required. Since the Cys does not interact with any residues in the simplified complex, its partial charges turned out to be rather similar to the Amber benchmarks except at the Fe-S interface and to some extent the Cβ region. These ligands are shown in Listings 5 and 6 (S1 File) for the oxidized and reduced structures, respectively.

The non-heme-Fe was decomposed into four structures. Two residues (HI1 and HI2) were constructed to model His183 and His187 on the non-heme Fe center, ASB was constructed to model Asp333 and a special Fe-OH_2_ ligand (FE2) was also constructed to complete the ROC structure (Listings 5 and 6 in S1 File).

The use of different His residues reflects the fact that the interaction between the bridging Asp180 tends to change the partial charges breaking the symmetry of the residues in the ROC and the [2Fe-2S] (FES). Hence HR2 (His93) and HI1 (His183) show a coupling that is not seen in HR1 (His71) and HI2 (His187). Indeed, those HR1 and HI2 (although different conformations of HID and HIE) show fairly strong similarity with the original Amber files, where HR2 and HI1 do not (see Tables 11-14 of main manuscript and S3 File [Excel Tables]). Interestingly, the Asp180, although involved in the DBA-complex, is not drastically altered from the Amber benchmarks, and therefore was left unchanged (data not shown).

Finally, the whole dioxygenase:ferredoxin complex must have an integral charge or the Leap program will strongly complain. For the Ferredoxin structure, the partial charges at the interface of the truncated structure and the extended structure were scaled in such a way as to satisfy the unit charge requirements. This consisted of either scaling the boundary positive charges (H, CA, C) to better match the Amber benchmarks or adjusting the boundary negative charges (N, CB, O) in a similar way. This scaling was generally kept to within ±5% of the set value. In short, small changes were searched for that would minimize the degree of change as much as possible. The process can be understood from the S3 File [Excel Tables].

The final partial charges are built such that each Rieske and ROC is of unit charge and the Rieske structure has a unit charge of exactly 0 or -1, depending on whether the structure is in the oxidized state or the reduced state. The last column of S3 File [Excel Tables] lists the adjusted partial charges and the scaling factors that were used are also indicated. The reader can see that the adjustments mostly happen on residues that heavily deviate from the Amber benchmarks parameters and are located in inconsequential regions of the main chain or the side chains, which are restrained by the ROC structure or the [2Fe-2S] structure (label FES) from the very start. Hence, there is some reasonable assurance that they are unlikely to skew any MD simulations, any more than the whole principle of a static force field applied to a quantum mechanical system that is inherently dynamical in nature skews the results.

See S1 Section 4.5 for examples on how to use leap to set up the top and crd files in Amber to build the full complex for running simulations.

### S1 Listing 5: Oxidized Rieske (Fe^3+^:Fe^3+^) structure and non-heme Fe^2+^ (S=5)

0 0 2

Rieske (His)2[2Fe-2S](Cys)2 cluster for the oxidized state (S_net = 5)

FES_I1v.m12

FES INT 0

CORRECT OMIT DU BEG

0.0000

1 DUMM DU M 0 -1 -2 0.000 .0 .0 0.00000

2 DUMM DU M 1 0 -1 1.449 .0 .0 0.00000

3 DUMM DU M 2 1 0 1.522 111.1 .0 0.00000

4 FE1 FE M 3 2 1 1.540 111.208 180.000 0.71963

5 S1 Sb E 4 3 2 2.114 142.534 -90.000 -0.57315

6 S2 Sb E 4 3 2 2.098 37.265 -91.759 -0.57315

7 FE2 FE M 4 3 2 2.538 90.000 -90.000 0.86659

LOOP

FE2 S1

FE2 S2

IMPROPER

DONE

Cysteine bound to Fe2S2 Rieske cluster: for 2DE5, Cys69 and 93.

CYR_I1v.m12

CYR INT 1

CORRECT OMIT DU BEG

0.0000

1 DUMM DU M 0 -1 -2 0.000 .0 .0 0.00000

2 DUMM DU M 1 0 -1 1.449 .0 .0 0.00000

3 DUMM DU M 2 1 0 1.522 111.1 .0 0.00000

4 N N M 3 2 1 1.540 116.600 180.000 -0.67071

5 H H E 4 3 2 1.020 119.800 0.000 0.31353

6 CA CT M 4 3 2 1.477 121.900 180.000 0.30844

7 HA H1 E 6 4 3 1.103 106.600 300.000 0.01714

8 CB CT 3 6 4 3 1.529 109.900 60.000 0.14191

9 HB2 HC E 8 6 4 1.100 109.900 300.000 0.00714

10 HB3 HC E 8 6 4 1.104 110.400 60.000 0.00714

11 SG SH E 8 6 4 1.848 111.600 180.000 -0.61565

12 C C M 6 4 3 1.556 109.500 180.000 0.64976

13 O O E 12 6 4 1.235 122.600 0.000 -0.57961

LOOP

IMPROPER

-M CA N H

CA +M C O

DONE

Histidine ligand of the Rieske structure (H epsilon): for 2DE5, His 71

HIR_I1v.m12

HR1 INT 1

CORRECT OMIT DU BEG

0.0000

1 DUMM DU M 0 -1 -2 0.000 .0 .0 0.00000

2 DUMM DU M 1 0 -1 1.449 .0 .0 0.00000

3 DUMM DU M 2 1 0 1.522 111.1 .0 0.00000

4 N N M 3 2 1 1.540 116.600 180.000 -0.59527

5 H H E 4 3 2 1.027 119.800 0.000 0.33875

6 CA CT M 4 3 2 1.470 121.900 180.000 0.47867

7 HA H1 E 6 4 3 1.106 106.100 300.000 -0.00816

8 CB CT 3 6 4 3 1.530 109.700 60.000 -0.17728

9 HB2 HC E 8 6 4 1.102 109.000 300.000 0.05965

10 HB3 HC E 8 6 4 1.104 109.500 60.000 0.05965

11 CG CC S 8 6 4 1.495 114.500 180.000 0.08745

12 ND1 NB S 11 8 6 1.404 121.700 180.000 -0.23650

13 CE1 CR B 12 11 8 1.339 107.000 180.000 0.03931

14 HE1 H5 E 13 12 11 1.086 125.700 180.000 0.16198

15 NE2 NA B 13 12 11 1.359 110.000 0.000 -0.23069

16 HE2 H E 15 13 12 1.015 125.700 180.000 0.36374

17 CD2 CW S 15 13 12 1.387 108.300 0.000 -0.12617

18 HD2 H4 E 17 15 13 1.086 121.900 180.000 0.16903

19 C C M 6 4 3 1.562 109.900 180.000 0.46967

20 O O E 19 6 4 1.229 122.900 0.000 -0.55866

LOOP

CD2 CG

IMPROPER

-M CA N H

CB CD2 CG ND1

HE1 NE2 CE1 ND1

CE1 CD2 NE2 HE2

CG HD2 CD2 NE2

CA +M C O

DONE

Histidine ligand of Rieske structure (H epsilon donor): for 2DE5, His 93

HIR_I1v.m12

HR2 INT 1

CORRECT OMIT DU BEG

0.0000

1 DUMM DU M 0 -1 -2 0.000 .0 .0 0.00000

2 DUMM DU M 1 0 -1 1.449 .0 .0 0.00000

3 DUMM DU M 2 1 0 1.522 111.1 .0 0.00000

4 N N M 3 2 1 1.540 116.600 180.000 -0.59527

5 H H E 4 3 2 1.027 119.800 0.000 0.33875

6 CA CT M 4 3 2 1.470 121.900 180.000 0.47867

7 HA H1 E 6 4 3 1.106 106.100 300.000 -0.00816

8 CB CT 3 6 4 3 1.530 109.700 60.000 -0.44731

9 HB2 HC E 8 6 4 1.102 109.000 300.000 0.12903

10 HB3 HC E 8 6 4 1.104 109.500 60.000 0.12903

11 CG CC S 8 6 4 1.495 114.500 180.000 0.40969

12 ND1 NB S 11 8 6 1.404 121.700 180.000 -0.49471

13 CE1 CR B 12 11 8 1.339 107.000 180.000 -0.10190

14 HE1 H5 E 13 12 11 1.086 125.700 180.000 0.19617

15 NE2 NA B 13 12 11 1.359 110.000 0.000 0.18190

16 HE2 H E 15 13 12 1.015 125.700 180.000 0.16988

17 CD2 CW S 15 13 12 1.387 108.300 0.000 -0.40840

18 HD2 H4 E 17 15 13 1.086 121.900 180.000 0.21834

19 C C M 6 4 3 1.562 109.900 180.000 0.46967

20 O O E 19 6 4 1.229 122.900 0.000 -0.55866

LOOP

CD2 CG

IMPROPER

-M CA N H

CB CD2 CG ND1

HE1 NE2 CE1 ND1

CE1 CD2 NE2 HE2

CG HD2 CD2 NE2

CA +M C O

DONE

ACTIVE CENTER: mono-nuclear Fe2+ with one water ligand

FE2

FE2 INT 1

CORR OMIT DU BEG

0.00000

1 DUMM DU M 0 -1 -2 0.000 0.000 0.000 0.00000

2 DUMM DU M 1 0 -1 1.000 0.000 0.000 0.00000

3 DUMM DU M 2 1 0 1.000 90.000 0.000 0.00000

4 FE FE M 3 2 1 1.000 90.000 180.000 1.36153

5 O OW M 3 2 1 2.058 90.000 180.000 -1.15153

6 H1 HW E 5 4 3 0.977 128.913 0.000 0.59542

7 H2 HW E 5 4 3 0.977 112.915 180.000 0.59542

LOOP

H1 H2

DONE

Histidine ligand bound to the mono-nuclear Fe2+ site: for 2DE5, His183

HIJ_I1v.m12 (original file)

HI1 INT 0

CORRECT OMIT DU BEG

0.0000

1 DUMM DU M 0 -1 -2 0.000 0.000 0.000 0.00000

2 DUMM DU M 1 0 -1 1.449 0.000 0.000 0.00000

3 DUMM DU M 2 1 0 1.522 111.100 0.000 0.00000

4 N N M 3 2 1 1.540 116.600 180.000 -0.63510

5 H H E 4 3 2 1.019 119.800 0.000 0.34275

6 CA CT M 4 3 2 1.470 121.900 180.000 0.27390

7 HA H1 E 6 4 3 1.104 106.100 300.000 0.02477

8 CB CT 3 6 4 3 1.543 110.900 60.000 -0.45486

9 HB2 HC E 8 6 4 1.101 109.100 300.000 0.14557

10 HB3 HC E 8 6 4 1.104 107.800 60.000 0.14557

11 CG CC S 8 6 4 1.498 114.100 180.000 0.32075

12 ND1 NA B 11 8 6 1.390 123.800 180.000 -0.32499

13 HD1 H E 12 11 8 1.017 126.100 0.000 0.37669

14 CE1 CR B 12 11 8 1.365 108.700 180.000 0.11989

15 HE1 H5 E 14 12 11 1.087 124.200 180.000 0.14310

16 NE2 Np S 14 12 11 1.337 110.100 0.000 -0.39576

17 CD2 CV S 16 14 12 1.394 106.200 0.000 -0.19768

18 HD2 H4 E 17 16 14 1.089 120.300 180.000 0.14547

19 C C M 6 4 3 1.562 111.300 180.000 0.71519

20 O O E 19 6 4 1.231 122.100 0.000 -0.55286

LOOP

CD2 CG

IMPROPER

-M CA N H

CB CD2 CG ND1

CG CE1 ND1 HD1

HE1 ND1 CE1 NE2

CG HD2 CD2 NE2

CA +M C O

DONE

Histidine ligand bound to the mono-nuclear Fe2+ site: for 2DE5, His187

HIT_I1v.m12 (original file)

HI2 INT 0

CORRECT OMIT DU BEG

0.0000

1 DUMM DU M 0 -1 -2 0.000 .0 .0 0.00000

2 DUMM DU M 1 0 -1 1.449 .0 .0 0.00000

3 DUMM DU M 2 1 0 1.522 111.1 .0 0.00000

4 N N M 3 2 1 1.540 116.600 180.000 -0.66036

5 H H E 4 3 2 1.019 119.800 0.000 0.33055

6 CA CT M 4 3 2 1.471 121.900 180.000 0.36189

7 HA H1 E 6 4 3 1.104 106.700 300.000 0.03106

8 CB CT 3 6 4 3 1.533 111.100 60.000 -0.46820

9 HB2 HC E 8 6 4 1.106 108.600 300.000 0.13926

10 HB3 HC E 8 6 4 1.104 107.200 60.000 0.13926

11 CG CC S 8 6 4 1.500 114.200 180.000 0.22043

12 ND1 NA B 11 8 6 1.392 121.600 180.000 -0.19588

13 HD1 H E 12 11 8 1.016 125.900 0.000 0.35010

14 CE1 CR B 12 11 8 1.371 108.800 180.000 0.04137

15 HE1 H5 E 14 12 11 1.086 124.500 180.000 0.14754

16 NE2 Nv S 14 12 11 1.341 109.500 0.000 -0.43888

17 CD2 CV S 16 14 12 1.396 106.800 0.000 -0.12801

18 HD2 H4 E 17 16 14 1.093 119.200 180.000 0.16057

19 C C M 6 4 3 1.564 111.000 180.000 0.69632

20 O O E 19 6 4 1.231 122.000 0.000 -0.54234

LOOP

CD2 CG

IMPROPER

-M CA N H

CB CD2 CG ND1

CG CE1 ND1 HD1

HE1 ND1 CE1 NE2

CG HD2 CD2 NE2

CA +M C O

DONE

Aspartic acid ligand bound to the mono-nuclear Fe2+ site: for 2DE5, Asp333

ASB_I1v.m12

ASB INT 0

CORRECT OMIT DU BEG

0.0000

1 DUMM DU M 0 -1 -2 0.000 .0 .0 0.00000

2 DUMM DU M 1 0 -1 1.449 .0 .0 0.00000

3 DUMM DU M 2 1 0 1.522 111.1 .0 0.00000

4 N N M 3 2 1 1.540 116.600 180.000 -0.59403

5 H H E 4 3 2 1.032 119.800 0.000 0.35347

6 CA CT M 4 3 2 1.472 121.900 180.000 0.23333

7 HA H1 E 6 4 3 1.107 107.600 300.000 0.03327

8 CB CT 3 6 4 3 1.539 111.500 60.000 -0.35546

9 HB2 HC E 8 6 4 1.105 107.300 300.000 0.09095

10 HB3 HC E 8 6 4 1.100 108.600 60.000 0.09095

11 CG C B 8 6 4 1.529 116.200 180.000 1.01384

12 OD1 OS E 11 8 6 1.295 114.900 -123.000 -0.90423

13 OD2 O2 E 11 8 6 1.261 119.900 57.000 -0.83617

14 C C M 6 4 3 1.568 110.700 180.000 0.63443

15 O O E 14 6 4 1.232 122.500 0.000 -0.53827

LOOP

IMPROPER

-M CA N H

CB OD1 CG OD2

CA +M C O

DONE

STOP

### S1 Listing 6: Reduced Rieske (Fe^2+^:Fe^3+^) structure and non-heme Fe^2+^ (S=6)

0 0 2

Rieske (His)2[2Fe-2S](Cys)2 cluster for the reduced state (S_tot = 6)

FES_I1v.m12

FES INT 0

CORRECT OMIT DU BEG

0.0000

1 DUMM DU M 0 -1 -2 0.000 .0 .0 0.00000

2 DUMM DU M 1 0 -1 1.449 .0 .0 0.00000

3 DUMM DU M 2 1 0 1.522 111.1 .0 0.00000

4 FE1 FE M 3 2 1 1.540 111.208 180.000 0.79968

5 S1 Sb E 4 3 2 2.114 142.534 -90.000 -0.77958

6 S2 Sb E 4 3 2 2.098 37.265 -91.759 -0.77958

7 FE2 FE M 4 3 2 2.538 90.000 -90.000 0.65086

LOOP

FE2 S1

FE2 S2

IMPROPER

DONE

Cysteine bound to Fe2S2 Rieske cluster: for 2DE6, Cys69 and 93.

CYR_I1v.m12

CYR INT 1

CORRECT OMIT DU BEG

0.0000

1 DUMM DU M 0 -1 -2 0.000 .0 .0 0.00000

2 DUMM DU M 1 0 -1 1.449 .0 .0 0.00000

3 DUMM DU M 2 1 0 1.522 111.1 .0 0.00000

4 N N M 3 2 1 1.540 116.600 180.000 -0.67071

5 H H E 4 3 2 1.020 119.800 0.000 0.31353

6 CA CT M 4 3 2 1.477 121.900 180.000 0.29030

7 HA H1 E 6 4 3 1.103 106.600 300.000 0.01714

8 CB CT 3 6 4 3 1.529 109.900 60.000 0.21701

9 HB2 HC E 8 6 4 1.100 109.900 300.000 -0.02168

10 HB3 HC E 8 6 4 1.104 110.400 60.000 -0.02168

11 SG SH E 8 6 4 1.848 111.600 180.000 -0.75803

12 C C M 6 4 3 1.556 109.500 180.000 0.63343

13 O O E 12 6 4 1.235 122.600 0.000 -0.57961

LOOP

IMPROPER

-M CA N H

CA +M C O

DONE

Histidine ligand of the Rieske structure (H epsilon): for 2DE6, His 71

HIR_I1v.m12

HR1 INT 1

CORRECT OMIT DU BEG

0.0000

1 DUMM DU M 0 -1 -2 0.000 .0 .0 0.00000

2 DUMM DU M 1 0 -1 1.449 .0 .0 0.00000

3 DUMM DU M 2 1 0 1.522 111.1 .0 0.00000

4 N N M 3 2 1 1.540 116.600 180.000 -0.59527

5 H H E 4 3 2 1.027 119.800 0.000 0.33875

6 CA CT M 4 3 2 1.470 121.900 180.000 0.47867

7 HA H1 E 6 4 3 1.106 106.100 300.000 -0.00816

8 CB CT 3 6 4 3 1.530 109.700 60.000 -0.05134

9 HB2 HC E 8 6 4 1.102 109.000 300.000 0.01710

10 HB3 HC E 8 6 4 1.104 109.500 60.000 0.01710

11 CG CC S 8 6 4 1.495 114.500 180.000 -0.06857

12 ND1 NB S 11 8 6 1.404 121.700 180.000 -0.05243

13 CE1 CR B 12 11 8 1.339 107.000 180.000 -0.05050

14 HE1 H5 E 13 12 11 1.086 125.700 180.000 0.18815

15 NE2 NA B 13 12 11 1.359 110.000 0.000 -0.28322

16 HE2 H E 15 13 12 1.015 125.700 180.000 0.36934

17 CD2 CW S 15 13 12 1.387 108.300 0.000 -0.07984

18 HD2 H4 E 17 15 13 1.086 121.900 180.000 0.15373

19 C C M 6 4 3 1.562 109.900 180.000 0.45763

20 O O E 19 6 4 1.229 122.900 0.000 -0.58295

LOOP

CD2 CG

IMPROPER

-M CA N H

CB CD2 CG ND1

HE1 NE2 CE1 ND1

CE1 CD2 NE2 HE2

CG HD2 CD2 NE2

CA +M C O

DONE

Histidine ligand of Rieske structure (H epsilon donor): for 2DE6, His 93

HIR_I1v.m12

HR2 INT 1

CORRECT OMIT DU BEG

0.0000

1 DUMM DU M 0 -1 -2 0.000 .0 .0 0.00000

2 DUMM DU M 1 0 -1 1.449 .0 .0 0.00000

3 DUMM DU M 2 1 0 1.522 111.1 .0 0.00000

4 N N M 3 2 1 1.540 116.600 180.000 -0.59527

5 H H E 4 3 2 1.027 119.800 0.000 0.33875

6 CA CT M 4 3 2 1.470 121.900 180.000 0.47867

7 HA H1 E 6 4 3 1.106 106.100 300.000 -0.00816

8 CB CT 3 6 4 3 1.530 109.700 60.000 -0.21572

9 HB2 HC E 8 6 4 1.102 109.000 300.000 0.06509

10 HB3 HC E 8 6 4 1.104 109.500 60.000 0.06509

11 CG CC S 8 6 4 1.495 114.500 180.000 0.28479

12 ND1 NB S 11 8 6 1.404 121.700 180.000 -0.47809

13 CE1 CR B 12 11 8 1.339 107.000 180.000 -0.11165

14 HE1 H5 E 13 12 11 1.086 125.700 180.000 0.18469

15 NE2 NA B 13 12 11 1.359 110.000 0.000 0.14437

16 HE2 H E 15 13 12 1.015 125.700 180.000 0.17054

17 CD2 CW S 15 13 12 1.387 108.300 0.000 -0.38474

18 HD2 H4 E 17 15 13 1.086 121.900 180.000 0.20801

19 C C M 6 4 3 1.562 109.900 180.000 0.45763

20 O O E 19 6 4 1.229 122.900 0.000 -0.58295

LOOP

CD2 CG

IMPROPER

-M CA N H

CB CD2 CG ND1

HE1 NE2 CE1 ND1

CE1 CD2 NE2 HE2

CG HD2 CD2 NE2

CA +M C O

DONE

ACTIVE CENTER: mono-nuclear Fe2+ with one water ligand

FE2

FE2 INT 1

CORR OMIT DU BEG

0.00000

1 DUMM DU M 0 -1 -2 0.000 0.000 0.000 0.00000

2 DUMM DU M 1 0 -1 1.000 0.000 0.000 0.00000

3 DUMM DU M 2 1 0 1.000 90.000 0.000 0.00000

4 FE FE M 3 2 1 1.000 90.000 180.000 1.37951

5 O OW M 3 2 1 2.058 90.000 180.000 -1.02011

6 H1 HW E 5 4 3 0.977 128.913 0.000 0.53089

7 H2 HW E 5 4 3 0.977 112.915 180.000 0.53089

LOOP

H1 H2

DONE

Histidine ligand bound to the mono-nuclear Fe2+ site: for 2DE6, His183

HIJ_I1v.m12 (original file)

HI1 INT 0

CORRECT OMIT DU BEG

0.0000

1 DUMM DU M 0 -1 -2 0.000 0.000 0.000 0.00000

2 DUMM DU M 1 0 -1 1.449 0.000 0.000 0.00000

3 DUMM DU M 2 1 0 1.522 111.100 0.000 0.00000

4 N N M 3 2 1 1.540 116.600 180.000 -0.63510

5 H H E 4 3 2 1.019 119.800 0.000 0.34275

6 CA CT M 4 3 2 1.470 121.900 180.000 0.27390

7 HA H1 E 6 4 3 1.104 106.100 300.000 0.02477

8 CB CT 3 6 4 3 1.543 110.900 60.000 -0.39105

9 HB2 HC E 8 6 4 1.101 109.100 300.000 0.13065

10 HB3 HC E 8 6 4 1.104 107.800 60.000 0.13065

11 CG CC S 8 6 4 1.498 114.100 180.000 0.27302

12 ND1 NA B 11 8 6 1.390 123.800 180.000 -0.28575

13 HD1 H E 12 11 8 1.017 126.100 0.000 0.36529

14 CE1 CR B 12 11 8 1.365 108.700 180.000 0.12371

15 HE1 H5 E 14 12 11 1.087 124.200 180.000 0.14479

16 NE2 Np S 14 12 11 1.337 110.100 0.000 -0.44190

17 CD2 CV S 16 14 12 1.394 106.200 0.000 -0.19122

18 HD2 H4 E 17 16 14 1.089 120.300 180.000 0.17595

19 C C M 6 4 3 1.562 111.300 180.000 0.71737

20 O O E 19 6 4 1.231 122.100 0.000 -0.55286

LOOP

CD2 CG

IMPROPER

-M CA N H

CB CD2 CG ND1

CG CE1 ND1 HD1

HE1 ND1 CE1 NE2

CG HD2 CD2 NE2

CA +M C O

DONE

Histidine ligand bound to the mono-nuclear Fe2+ site: for 2DE6, His187

HIT_I1v.m12 (original file)

HI2 INT 0

CORRECT OMIT DU BEG

0.0000

1 DUMM DU M 0 -1 -2 0.000 .0 .0 0.00000

2 DUMM DU M 1 0 -1 1.449 .0 .0 0.00000

3 DUMM DU M 2 1 0 1.522 111.1 .0 0.00000

4 N N M 3 2 1 1.540 116.600 180.000 -0.66037

5 H H E 4 3 2 1.019 119.800 0.000 0.33055

6 CA CT M 4 3 2 1.471 121.900 180.000 0.36189

7 HA H1 E 6 4 3 1.104 106.700 300.000 0.03106

8 CB CT 3 6 4 3 1.533 111.100 60.000 -0.43744

9 HB2 HC E 8 6 4 1.106 108.600 300.000 0.12667

10 HB3 HC E 8 6 4 1.104 107.200 60.000 0.12667

11 CG CC S 8 6 4 1.500 114.200 180.000 0.21867

12 ND1 NA B 11 8 6 1.392 121.600 180.000 -0.20180

13 HD1 H E 12 11 8 1.016 125.900 0.000 0.35504

14 CE1 CR B 12 11 8 1.371 108.800 180.000 0.06501

15 HE1 H5 E 14 12 11 1.086 124.500 180.000 0.14054

16 NE2 Nv S 14 12 11 1.341 109.500 0.000 -0.52348

17 CD2 CV S 16 14 12 1.396 106.800 0.000 -0.11600

18 HD2 H4 E 17 16 14 1.093 119.200 180.000 0.15312

19 C C M 6 4 3 1.564 111.000 180.000 0.69844

20 O O E 19 6 4 1.231 122.000 0.000 -0.54234

LOOP

CD2 CG

IMPROPER

-M CA N H

CB CD2 CG ND1

CG CE1 ND1 HD1

HE1 ND1 CE1 NE2

CG HD2 CD2 NE2

CA +M C O

DONE

Aspartic acid ligand bound to the mono-nuclear Fe2+ site: for 2DE6, Asp333

ASB_I1v.m12

ASB INT 0

CORRECT OMIT DU BEG

0.0000

1 DUMM DU M 0 -1 -2 0.000 .0 .0 0.00000

2 DUMM DU M 1 0 -1 1.449 .0 .0 0.00000

3 DUMM DU M 2 1 0 1.522 111.1 .0 0.00000

4 N N M 3 2 1 1.540 116.600 180.000 -0.59403

5 H H E 4 3 2 1.032 119.800 0.000 0.35347

6 CA CT M 4 3 2 1.472 121.900 180.000 0.23833

7 HA H1 E 6 4 3 1.107 107.600 300.000 0.03327

8 CB CT 3 6 4 3 1.539 111.500 60.000 -0.31731

9 HB2 HC E 8 6 4 1.105 107.300 300.000 0.08380

10 HB3 HC E 8 6 4 1.100 108.600 60.000 0.08380

11 CG C B 8 6 4 1.529 116.200 180.000 0.93253

12 OD1 OS E 11 8 6 1.295 114.900 -123.000 -0.78525

13 OD2 O2 E 11 8 6 1.261 119.900 57.000 -0.87719

14 C C M 6 4 3 1.568 110.700 180.000 0.63443

15 O O E 14 6 4 1.232 122.500 0.000 -0.53827

LOOP

IMPROPER

-M CA N H

CB OD1 CG OD2

CA +M C O

DONE

STOP

## 4.3 Building the Force Field (frcmod)

Whenever possible, already existing parameters for bond stretching and bending were favored when available. If no such values were available, then the next step was to look for any available experimental data, favoring these values over calculated ones. Hence, as much as possible, the force field parameters were borrowed from gaff when reasonable equivalents for bond stretching, bending and dihedral angles could be found.

Since the SHAKE algorithm is used, the high frequency modes can be treated as static on the 2 fs time step sampling interval of MD simulations. The Van der Waals interactions tend to impose further constraints on the remaining freedom of movement of the amino acids bound to the [2Fe-2S] and neighboring amino acids in the corresponding chains.

Calculations of these force field parameters were done with both g03 and g09. All reported results are from g09 calculations; however, the differences for bond parameters are not so drastically different.

### 4.3.1 Direct calculation of the force field:

Calculation of the force constants is a very inexact approach in general because the vibrations describing normal modes involve large substructures that are all highly coupled, yet the only parameters at our discretion are individual bond stretching, bending and dihedral angles. The dihedral angles are perhaps the most separable of the three since they are typically an order of magnitude smaller than the bending energies. However, in ring structures like [2Fe-2S], the various symmetries of the normal modes (breathing, flapping like a bird between the S and Fe in the [2Fe-2S] ring, and numerous other symmetric or antisymmetric coupling effects of substructures) are not easily described by these narrowly defined parameters. Thus, directly estimating the force constants turned out to be particularly challenging in this problem because there are few ways to isolate the particular excitation: as in the case of a simple molecule like ethane, which is largely where this parameterization approach was originally conceived.

Nevertheless, to add weight to experimentally obtained parameters, we also tried estimating these values directly from finite difference techniques using g03: optimizing the structure for a incrementally different Fe-Fe bond distance in the symmetric 4-Cys-[2Fe-2S], using the prior AF spin polarized optimization and solving for several points very close to the equilibrium position (±0.005 Å). The constructed structures were optimized with constraints on the specific bond distance and on ligands bound to the Fe that might rotate. Initially, we had tried the Scan option; however, the second derivatives tended to be far too overestimated, perhaps due to excessive distortion.

A footnote: when using the Scan in the route, the atomic arrangements of the surrounding atoms cannot be optimized to find the most stable configuration of the distortion. Building individual structures and optimizing them with a single bond distance constraint and possibly a few rotational constraints allowed the electronic structure and surrounding atoms to adjust more naturally to the distortion.

The second derivative from the finite difference calculations contains components of both the effective spring constant and the weight of the reduced mass. Because the A_g_ symmetric Fe-Fe normal mode at 210 cm^-1^ involves the two irons and the two sulfurs within the plain, the reduced mass is not simply that of the Fe-Fe bond (27.9 amu, from $\mu_{\text{Fe-Fe}}=m_{\text{Fe}}/2$), rather it is associated with all the atoms involved combined with the particular interaction $\mu_{\text{Fe-Fe}}=m_{\text{Fe}}m_{\text{Fe}}/(2m_{\text{Fe}}+2m_{\text{S}})$, which is closer to a weight of 1/3. Hence, for the Fe-Fe bond, the finite difference yielded 0.75 mdyne/Å, and after weighing by the reduced mass correction, this yielded 0.23 mdyne/Å (33 kcal/mol·Å^2^). The value is close to that reported from experimental studies of 4-Cys-[2Fe-2S] related structures. Hence, at least with careful consideration of symmetry in the structure and the effective reduced mass, it is possible to estimate these values quantitatively.

### 4.3.2 Rieske structure:

Force field parameters were based upon experimental data from resonance Raman spectroscopy measurements of the Fe-S bonds in (His)_2_[2Fe-2S](Cys)_2_ (Rieske) [[54-56](#_ENREF_54)] and (Cys)_2_[2Fe-2S](Cys)_2_ (4-Cys) structures [[55](#_ENREF_55),[57-59](#_ENREF_57)] in conjunction with QM calculations using g09. For Fe-S stretching modes, the QM predicted values and the experimental values agreed within 10% of each other (Table 7 in the main text and S2 File, “Rieske normal modes”). The remaining parts within the ligands of the structure are only estimated based upon the values obtained from the QM calculation or from experiment.

Dihedral angles had to be handled separately for the His in the Fe center and the Rieske center. To obtain force field parameters for the dihedral angles associated with rotation about the Fe-Nδ bond or the Fe-Sγ bond, Cys and His ligand bound to the Rieske were rotated one at a time and optimized using two constraints: one to keep the rotated dihedral angle in place and the other to prevent the neighboring ligands from rotating excessively. Only two constraints are used because we assume that the rate at which these residues rotate is much slower than the resulting response of the structure to small changes in the orientation of some of its ligands.

From these rotated structures, an approximate dihedral angle function was obtained. The results of this rotation about the Fe-N bond are plotted in S1C Figure (panel a) and a fit of these data are shown using a combination of three dihedral angle functions.

### 4.3.3 Non-Heme Fe-center:

For the Fe-center, there is very little experimental data available: particularly for the monodentate. Therefore, all that could be done was to calculate the Fe-N and Fe-O stretching parameters using g09 and applying the lessons learned from solving the Rieske and 4-Cys-[2Fe-2S] structures. An experimental value for the Fe-O stretch in a catacholate Fe complex (forming a bidentate FeO bond) is 583 cm^-1^ [[60](#_ENREF_60),[61](#_ENREF_61)] and monodentate Fe-O vibrational structure can be inferred to be around 570 cm^-1^ [[62](#_ENREF_62),[63](#_ENREF_63)]. In Fig 8 (main text), it appears that the characteristic bidentate frequency in the catacholate signal is the result of bending of the Cγ−Oδ−Fe bond of the Asp ligand. This can be seen in the assignments 598 cm^−1^ and 592 cm^−1^ for the monodentate and bidentate structures, respectively. This is consistent with the observed frequency of 570 to 583 cm^−1^. Hence, from the vibrational data in Fig 8 (main text), this largely corresponds to motion of the carboxyl-Fe bond of the bidentate Asp or a water coordinated monodentate Asp. In Fig 8 (main text), there are clear normal modes for Fe-N stretching at 238 and 252 cm^-1^ in the calculated normal modes. The Fe-OAc stretching modes appear at 302 cm^-1^ and the Fe-OH_2_ bond stretching occurs at 483 cm^-1^. In the oxobridge of the diiron(III) cluster (Fe-O-Fe), this value is 514 cm^-1^[[64](#_ENREF_64)]. Hence, the latter frequency is consistent with the predicted Fe-OH_2_ bond stretching mode.


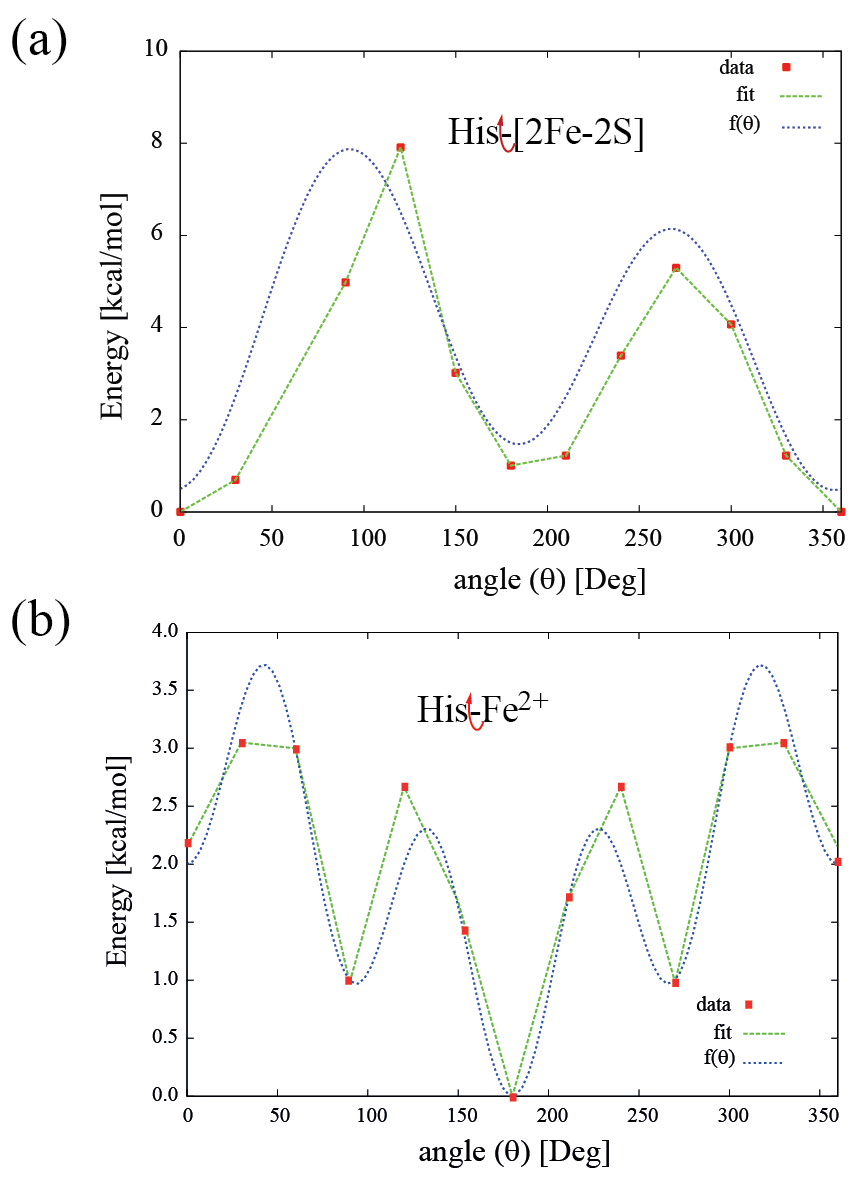


**S1 Figure C.**  **Fit of the energy of dihedral angle for His rotation around Fe bonds.** (a) Rotation of the His residue about the Fe in the (Cys)_2_[2Fe-2S](His)_2_ structure. (b) The change in energy as the H183 is rotated about the Fe-Nε bond of the ROC.

To obtain dihedral angles, as with the ROC structure, the H183, H187 (imidazole) and D333 (acetate) ligands within the ROC were rotated one at a time about the complex with the other structures held in their default positions and orientations (those found in the PDB files for these structures): Fe-Nε and Fe-Oδ respectively. For example, S1C Figure (panel b) shows the rotation of the H183 ring about the Fe-Nε axis, where the ring was rotated every 30^o^ to obtain the approximate energy profile.

The major issue with the ROC is that a dry environment (i.e., one where there is no free molecular water present) results in a bidentate optimal structure for the ROC (S1D Figure, panel a), which is counter to the observed structure in the case of 1WW9 and 2DE5-7. Therefore, it was necessary to develop procedures to optimize water molecules around the Asp333 residue. A considerable number of trials were done to generate a clearly optimal structure as shown in S1D Figure (panel b). Nevertheless, the structure shown in S1D Figure (panel b) is a fully optimized structure that was reproduced several times. The method for optimizing the water molecules will be reported elsewhere.


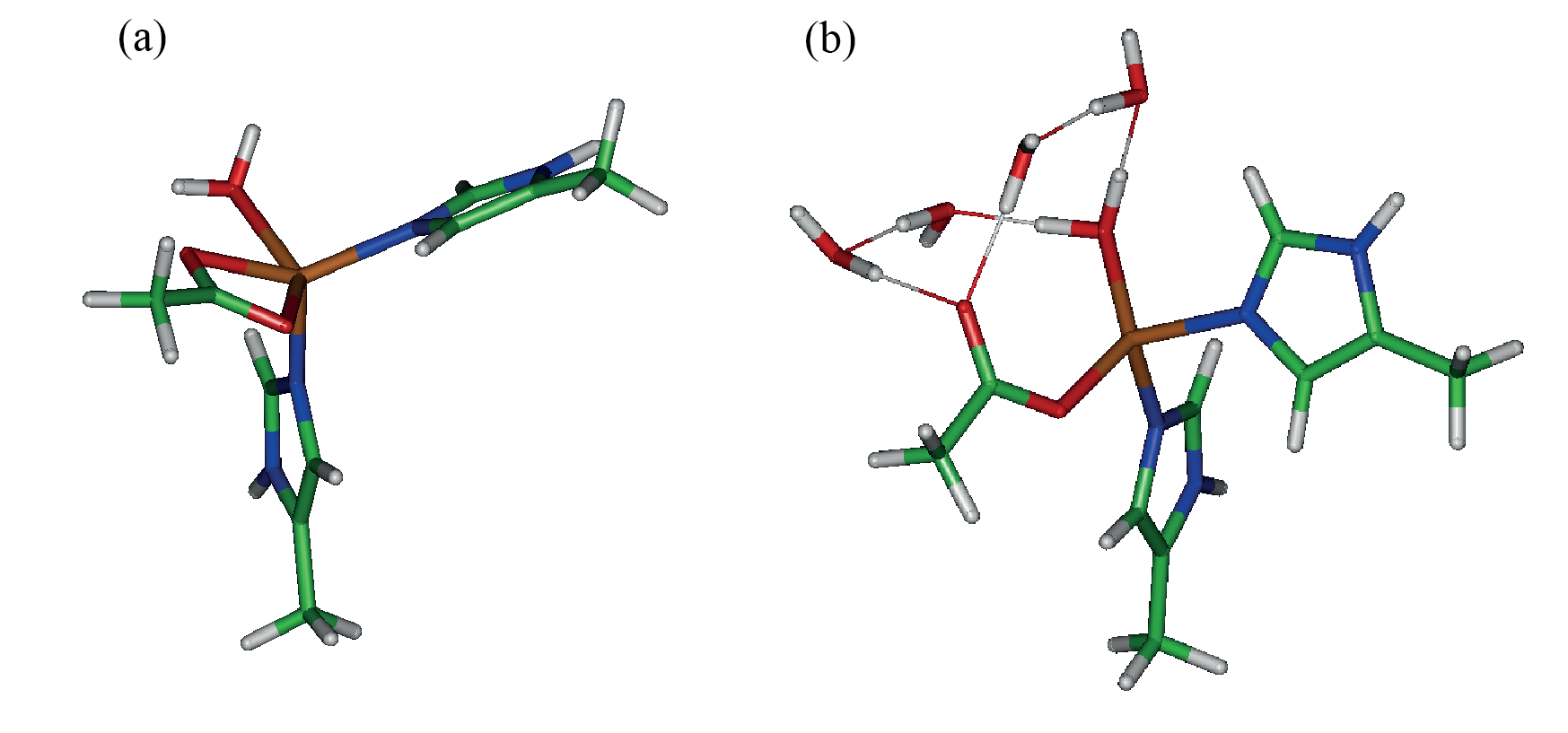


**S1 Figure D. Optimal structure of the non-heme Fe reactive oxygen center with and without water.** (a) dry condition and (b) wet condition.

## 4.4 Results of frcmod files

In the pages that follow, the results of the determined frcmod file (Listing 7 in S1 File) will be presented and then the interactions are broken down into a bit more detail (Listing 8 and 9 in S1 File). These files are provided in S4 File as a zipped folder.

### S1 Listing 7: Force field spectral parameters

remark goes here

MASS

FE 55.80 1.000

Sb 32.06 2.900 bridge sulfur, same as ss

SH 32.060 2.900 ligand sulfur, same as ss

OS 16.000 0.465 (same as os)

Np 14.010 0.530 (same as n2)

Nv 14.010 0.530 (same as n2)

BOND

FE-FE 39.00 2.64 estimated from QC calc and experiment

FE-SH 220.00 2.261 estimated from QC calc and experiment

FE-Sb 200.00 2.184 estimated from QC calc and experiment

FE-NB 140.00 2.089 estimated from QC calc and experiment

FE-OS 86.02 2.013 (d from 2Asp+Asn(wc))

FE-Np 311.60 2.020 (d from 2Asp+Asn(wc))

FE-Nv 378.80 2.031 (d from 2Asp+Asn(wc))

FE-OW 137.30 2.028 (d from 2Asp+Asn(wc))

OS-C 411.30 1.291 (d from 2Asp+Asn(wc), F from c -os)

Np-CR 411.10 1.352 (d from 2Asp+Asn(wc), F from c2-na)

Np-CV 411.10 1.396 (d from 2Asp+Asn(wc), F from c2-na)

Nv-CR 411.10 1.345 (d from 2Asp+Asn(wc), F from c2-na)

Nv-CV 411.10 1.393 (d from 2Asp+Asn(wc), F from c2-na)

ANGLE

FE-Sb-FE 33.000 73.900 PDB averages and QC

FE-SH-CT 21.700 102.525 PDB averages and QC

FE-FE-Sb 40.00 52.841 PDB averages and QC

FE-FE-NB 13.000 133.400 QC averages

Sb-FE-Sb 38.900 105.900 PDB averages and QC

Sb-FE-SH 36.000 114.300 PDB averages and QC

Sb-FE-NB 36.000 107.500 PDB averages and QC

SH-FE-SH 28.800 90.300 PDB averages and QC

SH-FE-FE 8.600 130.500 QC averages

SH-CT-HC 42.700 107.870 (same as hc-c3-sh)

NB-FE-NB 36.000 89.000 from QM, avg PDB and table 1 of RR (Vittal et al.)

FE-NB-CR 21.600 123.532 ""

FE-NB-CC 21.600 128.671 ""

FE-OS-C 116.500 103.000 (d from 2Asp+Asn(wc) + F(th) from qm calc, maybe should lower F(th))

FE-Np-CR 31.500 127.000 (d from 2Asp+Asn(wc) + F(th) from qm calc)

FE-Np-CV 31.500 127.000 (d from 2Asp+Asn(wc) + F(th) from qm calc)

FE-Nv-CR 31.500 127.000 (d from 2Asp+Asn(wc) + F(th) from qm calc)

FE-Nv-CV 31.500 127.000 (d from 2Asp+Asn(wc) + F(th) from qm calc)

FE-OW-HW 35.000 128.000 (d from 2Asp+Asn(wc) + F(th) from qm calc, rounded)

OS-FE-Np 35.000 103.000 (d from 2Asp+Asn(wc) + F(th) from qm calc, rounded)

OS-FE-Nv 35.000 95.000 (d from 2Asp+Asn(wc) + F(th) from qm calc, rounded)

OS-FE-OW 70.000 130.000 (d from 2Asp+Asn(wc) + F(th) from qm calc, rounded)

Np-FE-Nv 35.000 110.000 (d from 2Asp+Asn(wc) + F(th) from qm calc, rounded)

Np-FE-OW 50.000 130.000 (d from 2Asp+Asn(wc) + F(th) from qm calc, rounded): QM ~35.00, but unstable

Nv-FE-OW 50.000 110.000 (d from 2Asp+Asn(wc) + F(th) from qm calc, rounded): QM ~35.00, but unstable

OS-C -O2 76.200 123.800 (d from 2Asp+Asn(wc) + F(th) from o -c -os)

OS-C -CT 69.300 116.100 (d from 2Asp+Asn(wc) + F(th) from c3-c -os)

Np-CR-NA 73.700 111.700 (d from 2Asp+Asn(wc) + F(th) from na-c2-na)

Np-CR-H5 51.200 124.800 (d from 2Asp+Asn(wc) + F(th) from ha-c2-na)

Np-CV-CC 69.800 109.200 (d from 2Asp+Asn(wc) + F(th) from c2-c2-na)

Np-CV-H4 51.200 121.400 (d from 2Asp+Asn(wc) + F(th) from ha-c2-na)

CR-Np-CV 67.800 105.300 (d from 2Asp+Asn(wc) + F(th) from c2-na-c2)

Nv-CR-NA 73.700 111.300 (d from 2Asp+Asn(wc) + F(th) from na-c2-na)

Nv-CR-H5 51.200 125.900 (d from 2Asp+Asn(wc) + F(th) from ha-c2-na)

Nv-CV-CC 69.800 109.600 (d from 2Asp+Asn(wc) + F(th) from c2-c2-na)

Nv-CV-H4 51.200 121.300 (d from 2Asp+Asn(wc) + F(th) from ha-c2-na)

CR-Nv-CV 67.800 105.400 (d from 2Asp+Asn(wc) + F(th) from c2-na-c2)

DIHE

FE-Sb-FE-FE 1 0.000 0.000 0.000 Not used because it is meaningless

FE-Sb-FE-Sb 1 5.000 180.000 1.000 similar order: His(CR-NB-CC-CW or CR-NB-CC-CT)

FE-SF-FE-NB 1 0.380 180.000 -3.000 new: same as hc-c3-c2-c2

FE-SF-FE-NB 1 1.150 0.000 1.000 additional term

FE-SH-CT-HC 1 0.156 0.000 3.000 same as X -c3-c3-X (used X=H1)

FE-SH-CT-CT 1 0.156 0.000 3.000 same as X -c3-c3-X (used X=CT)

FE-FE-NB-CR 1 4.000 180.000 4.000 from rotation (barrier much larger)

FE-FE-NB-CC 1 4.000 180.000 4.000 from rotation (barrier much larger)

CT-SH-FE-Sb 1 1.000 0.000 3.000 Cb-Sg-Fe-Sb

Sb-FE-FE-Sb 1 5.000 0.000 1.000 similar order: His(CR-NB-CC-CW or CR-NB-CC-CT)

Sb-FE-FE-NB 1 1.000 180.000 2.000 normal mode coupling not so clear

Sb-FE-FE-SH 1 1.000 180.000 2.000 normal mode coupling not so clear

Sb-FE-NB-CR 1 0.500 180.000 -12.000 **Not so simple to pull out specific dih angs.

Sb-FE-NB-CR 1 0.500 0.000 4.000 additional term

Sb-FE-NB-CC 1 0.500 180.000 -12.000 **Not so simple to pull out specific dih angs.

Sb-FE-NB-CC 1 0.500 0.000 4.000 additional term

SH-FE-Sb-FE 1 0.380 180.000 -3.000 new: same as hc-c3-c2-c2

SH-FE-Sb-FE 1 1.150 0.000 1.000 additional term

SH-FE-SH-CT 1 1.000 0.000 3.000 Sg-Fe-Sg-Cb

SH-FE-FE-NB 2 4.000 180.000 2.000 doesn't bend much but can bend

CT-SH-FE-FE 1 0.300 180.000 3.000 encourage a +/-120 deg position

FE-NB-CR-X 4 20.000 180.000 2.000 same as twice "X -CR-NB-X" with X=NA,H5,CW,CT

NB-FE-Sb-FE 1 0.380 180.000 -3.000 new: same as hc-c3-c2-c2

NB-FE-Sb-FE 1 1.150 0.000 1.000 additional term

CR-NB-FE-NB 1 1.000 180.000 4.000 CR-> His:Ce QM scan on Fe2

CC-NB-FE-NB 1 1.000 180.000 4.000 CC-> His:Cg

FE-OS-C -O2 1 0.625 180.000 2.000 (dha: 2DE6 th= 208; f: X -c2-na-X)

FE-OS-C -CT 1 0.625 180.000 2.000 (dha: 2DE6 th=-154; f: X -c2-na-X)

FE-Np-CR-X 4 9.600 180.000 2.000 same as twice "X -CV-NB-X" with X=NA,H5,CC,H4

FE-Nv-CR-X 4 9.600 180.000 2.000 same as twice "X -CV-NB-X" with X=NA,H5,CC,H4

X -Nv-FE-X 2 2.500 180.000 2.000 (dha: 2DE6/QM; f: doubled X -c2-na-X) X1=OS, X2=CR,CV

X -Np-FE-X 2 2.500 180.000 2.000 (dha: 2DE6/QM; f: doubled X -c2-na-X) X1=OS, X2=CR,CV

OS-FE-OW-HW 1 1.250 90.000 2.000 (dha: 2DE6/QM; f: doubled X -c2-na-X)

C -OS-FE-Np 1 0.625 144.000 2.000 (dha: 2DE6; f: X -c2-na-X)

C -OS-FE-Nv 1 0.625 -6.000 2.000 (dha: 2DE6; f: X -c2-na-X)

C -OS-FE-OW 1 3.625 218.000 2.000 (dha: 2DE6; f: X -c2-na-X)

Np-FE-Nv-CR 1 0.625 132.000 2.000 (dha: 2DE6; f: X -c2-na-X)

Np-FE-Nv-CV 1 0.625 110.000 2.000 (dha: 2DE6; f: X -c2-na-X)

Np-FE-OW-HW 1 0.625 75.000 3.000 (dha: 2DE6/QM cmprms; f: X -c2-na-X)

Nv-FE-Np-CR 1 0.625 32.000 2.000 (dha: 2DE6; f: X -c2-na-X, rrdr)

Nv-FE-Np-CV 1 0.625 66.000 2.000 (dha: 2DE6; f: X -c2-na-X, rrdr)

Nv-FE-OW-HW 1 0.625 80.000 2.000 (dha: 2DE6/QM cmprms; f: X -c2-na-X)

CV-Np-FE-OW 1 3.000 150.000 2.000 (dha: 2DE6; f: X -c2-na-X)

CR-Np-FE-OW 1 3.000 150.000 2.000 (dha: 2DE6; f: X -c2-na-X)

CV-Nv-FE-OW 1 0.625 19.000 2.000 (dha: 2DE6; f: X -c2-na-X)

CR-Nv-FE-OW 1 0.625 19.000 2.000 (dha: 2DE6; f: X -c2-na-X)

FE-OW-HW-HW 1 0.000 0.000 0.000 meaningless, but must write something

OS-C -CT-HC 1 0.000 180.000 3.000 (same as X -c -c3-X)

Np-CR-NA-CC 1 0.625 180.000 2.000 (same as X -c2-na-X)

Np-CR-NA-H 1 0.625 180.000 2.000 (same as X -c2-na-X)

Np-CV-CC-NA 1 6.650 180.000 2.000 (same as X -c2-c2-X)

Np-CV-CC-CT 1 6.650 180.000 2.000 (same as X -c2-c2-X)

Nv-CR-NA-X 2 4.650 180.000 2.000 (same as half X -CR-NA-X ) X=CC,H

Nv-CV-CC-X 2 10.300 180.000 2.000 (same as half X -CC-CV-X ) X=NA,CT

CR-Np-CV-X 2 9.500 180.000 2.000 (same as X -cc-nc-X ) X=CC,H4

CV-Np-CR-X 2 8.300 180.000 2.000 (same as X -c2-n2-X, rrdr, X=NA,H5)

CR-Nv-CV-X 2 9.500 180.000 2.000 (same as X -cc-nc-X ) X=CC,H4

CV-Nv-CR-X 2 8.300 180.000 2.000 (same as X -c2-n2-X, rrdr, X=NA,H5)

IMPROPER

CC-CR-NB-FE 1.1 180.0 2.0 Using default value

CC-CR-NA-H 1.1 180.0 2.0 Using default value

CT-O2-C -OS 1.1 180.0 2.0 Using default value

CR-CV-Np-FE 1.1 180.0 2.0 Using default value

CT-CV-CC-NA 1.1 180.0 2.0 Using default value

C*-CN-CB-CA 1.1 180.0 2.0 Default (atoms are: CG CE2 CD2 CE3)

NA-CA-CN-CB 1.1 180.0 2.0 Default (atoms are: NE1 CZ2 CE2 CD2)

H5-NA-CR-Np 1.1 180.0 2.0 Using default value

CC-HC-CV-Np 1.1 180.0 2.0 Using default value

CR-CV-Nv-FE 1.1 180.0 2.0 Using default value

H5-NA-CR-Nv 1.1 180.0 2.0 Using default value

CC-H4-CV-Nv 1.1 180.0 2.0 Using default value

NONBON

FE 1.2000 0.0500 taken from heme

Sb 2.0000 0.2500 same as ss

SH 2.0000 0.2500 same as ss

OS 1.6837 0.1700 (same as os)

Np 1.8240 0.1700 (same as nc)

Nv 1.8240 0.1700 (same as nc)

### S1 Listing 8: Details of Rieske dihedral angles parameters

Out of plain bending of the [2Fe-2S] ring

FE-Sb-FE-Sb 1 5.000 180.000 1.000

Sb-FE-FE-Sb 1 5.000 0.000 1.000

similar order: His(CR-NB-CC-CW or CR-NB-CC-CT)

CR-NB-CC-CW = Cε1-Nδ1-Cγ-Cδ2

CR-NB-CC-CT = Cε1-Nδ1-Cγ-Cβ

Bending between the Cys.Sγ and the His.Nδ across the Fe-Fe bond

SH-FE-FE-NB 2 4.000 180.000 2.000

doesn't bend much but can bend

Cysteine ligand

Interactions of [2Fe-2S] with the Cysteine ligand

Bending of Cys.Sγ relative to the [2Fe-2S] plain

SH-FE-Sb-FE 1 0.380 180.000 -3.000

SH-FE-Sb-FE 1 1.150 0.000 1.000

Based on the values from gaff parameters: hc-c3-c2-c2

Sb-FE-FE-SH 1 1.000 180.000 2.000

Sb-Fe-Fe-Sγ: normal mode coupling not so clear. This is a mere guess.

Rotation of Cys.Cβ around the Sγ-Fe axis

CT-SH-FE-FE 1 0.300 180.000 3.000

Encourage a +/-120 deg position

CT-SH-FE-Sb 1 1.000 0.000 3.000

Cβ-Sγ-Fe-Sb: guess

Rotation of γ-methyl group around the Sγ-Cβ axis

FE-SH-CT-HC 1 0.156 0.000 3.000

Fe-Sγ-Cβ-Hβ: same as X -c3-c3-X (with X=H1)

FE-SH-CT-CT 1 0.156 0.000 3.000

Fe-Sγ-Cβ-Cα: same as X -c3-c3-X (with X=CT)

Rotation of Cβ relative to the neighboring Cysteine ligand

SH-FE-SH-CT 1 1.000 0.000 3.000

Sγ-Fe-Sγ-Cβ

Interactions of [2Fe-2S] with the Histidine ligand

Bending of Nδ relative the [2Fe-2S] plain

NB-FE-Sb-FE 1 0.380 180.000 -3.000

NB-FE-Sb-FE 1 1.150 0.000 1.000

Based on the values from gaff parameters: hc-c3-c2-c2

Sb-FE-FE-NB 1 1.000 180.000 2.000

normal mode coupling not so clear. This is a mere guess.

Rotation of the His ring around [2Fe-2S], rotation around the Fe-Nδ bond

Motion of the Cε1

Sb-FE-NB-CR 1 0.500 180.000 -12.000

Sb-FE-NB-CR 1 0.500 0.000 4.000

**Not so simple to pull out specific dih angs.

FE-FE-NB-CR 1 4.000 180.000 4.000

Data taken from QM calculation (with optimization) of the rotation of the His structure about the [2Fe-2S]: the barrier is actually much larger as the angle increases.

Out of plain bending of Fe and atoms in Histidine ring.

CR-NB-FE-NB 1 1.000 180.000 4.000

CR-> His:Cε1, QM scan.

FE-NB-CR-X 4 20.000 180.000 2.000

same as twice "X -CR-NB-X" with X=NA,H5,CW,CT

Motion of the Cδ1

Sb-FE-NB-CC 1 0.500 180.000 -12.000

Sb-FE-NB-CC 1 0.500 0.000 4.000

**Not so simple to pull out specific dih angs.

FE-FE-NB-CC 1 4.000 180.000 4.000

Data taken from QM calculation (with optimization) of the rotation of the His structure about the [2Fe-2S]: the barrier is actually much larger as the angle increases.

Out of plain bending of Fe and atoms in Histidine ring.

CC-NB-FE-NB 1 1.000 180.000 4.000

CC-> His:Cγ

FE-NB-CC-X 4 20.000 180.000 2.000

same as twice "X -CC-NB-X" with X=NA,H5,CW,CT

### S1 Listing 9: Details of non-Heme Iron dihedral angle parameters

Fe-His183

Out of plain bending parameters

FE-Np-CR-X 4 9.600 180.000 2.000

Same as gaff parameters: twice "X -CV-NB-X" with X=NA,H5,CC,H4

X -Np-FE-X 2 2.500 180.000 2.000

Dihedral angle in 2DE6: θ= 180, used same as gaff parameters: doubled X -c2-na-X; X1=OS, X2=CR,CV

Np-CR-NA-CC 1 0.625 180.000 2.000

Same as gaff parameters: X -c2-na-X

Np-CR-NA-H 1 0.625 180.000 2.000

Same as gaff parameters: X -c2-na-X

Np-CV-CC-NA 1 6.650 180.000 2.000

Same as gaff parameters: X -c2-c2-X

Np-CV-CC-CT 1 6.650 180.000 2.000

Same as gaff parameters: X -c2-c2-X

CR-Np-CV-X 2 9.500 180.000 2.000

Same as gaff parameters: X -cc-nc-X: X=CC,H4

CV-Np-CR-X 2 8.300 180.000 2.000

Same as gaff parameters: X -c2-n2-X, rrdr, X=NA,H5

Fe-His187

Out of plain bending parameters

FE-Nv-CR-X 4 9.600 180.000 2.000

same as Amber parameters: twice "X -CV-NB-X" with X=NA,H5,CC,H4

X -Nv-FE-X 2 2.500 180.000 2.000

Dihedral angle in 2DE6: θmin= -90,0,90, used same as gaff parameters: doubled X -c2-na-X; X1=OS, X2=CR,CV

Nv-CR-NA-X 2 4.650 180.000 2.000

Same as gaff parameters: half X -CR-NA-X; X=CC,H

Nv-CV-CC-X 2 10.300 180.000 2.000

Same as gaff parameters: half X -CC-CV-X; X=NA,CT

CR-Nv-CV-X 2 9.500 180.000 2.000

Same as gaff parameters: X -cc-nc-X; X=CC,H4

CV-Nv-CR-X 2 8.300 180.000 2.000

Same as gaff parameters: X -c2-n2-X; X=NA,H5)

Fe-Asp333

Rotation about the Fe

FE-OS-C -O2 1 0.625 180.000 2.000

Dihedral angle in 2DE6: θ= 208, used same as gaff parameters: X -c2-na-X

FE-OS-C -CT 1 0.625 180.000 2.000

Dihedral angle in 2DE6: θ= 154, used same as gaff parameters: X -c2-na-X)

OS-C -CT-HC 1 0.000 180.000 3.000

Same as gaff parameters: X -c -c3-X

His183-Fe-Asp333

C -OS-FE-Np 1 0.625 144.000 2.000

Dihedral angle in 2DE6: θ=144,used same as gaff parameters: X -c2-na-X

His187-Fe-Asp333

C -OS-FE-Nv 1 0.625 -6.000 2.000

Dihedral angle in 2DE6: θ= -6.0, used same as gaff parameters: X -c2-na-X)

His183-Fe-His187

Np-FE-Nv-CR 1 0.625 132.000 2.000

Dihedral angle in 2DE6: θ= 132, used same as gaff parameters: X -c2-na-X)

Np-FE-Nv-CV 1 0.625 110.000 2.000

Dihedral angle in 2DE6: θ= 110, used same as gaff parameters: X -c2-na-X

Nv-FE-Np-CV 1 0.625 66.000 2.000

Dihedral angle in 2DE6: θ= 66, used same as gaff parameters: X -c2-na-X

Nv-FE-Np-CR 1 0.625 32.000 2.000

Dihedral angle in 2DE6: θ= 32, used same as gaff parameters: X -c2-na-X

His183-Fe-water

Np-FE-OW-HW 1 0.625 75.000 3.000

Dihedral angle in 2DE6: θ= 75 and comparison of rmsd in other structures, used same as gaff parameters: X -c2-na-X

CV-Np-FE-OW 1 3.000 150.000 2.000

Dihedral angle in 2DE6: θ= 150, used same as gaff parameters: X -c2-na-X

CR-Np-FE-OW 1 3.000 150.000 2.000

Dihedral angle in 2DE6: θ= 150, used same as gaff parameters: X -c2-na-X

His187-Fe-water

Nv-FE-OW-HW 1 0.625 80.000 2.000

Dihedral angle in 2DE6: θ= 80 and comparison of rmsd in other structures, used same as gaff parameters: X -c2-na-X

CV-Nv-FE-OW 1 0.625 19.000 2.000

Dihedral angle in 2DE6: θ= 19, used same as gaff parameters: X -c2-na-X

CR-Nv-FE-OW 1 0.625 19.000 2.000

Dihedral angle in 2DE6: θ= 19, used same as gaff parameters: X -c2-na-X

Asp333-Fe-water

OS-FE-OW-HW 1 1.250 90.000 2.000

Dihedral angle in 2DE6: θ= 90, used same as gaff parameters: doubled X -c2-na-X

C -OS-FE-OW 1 3.625 218.000 2.000

Dihedral angle in 2DE6: θ= 218, used same as gaff parameters: X -c2-na-X

Fe-water

FE-OW-HW-HW 1 0.000 0.000 0.000

Meaningless, but must write something

## 4.5 Implementing the force field for MD simulations

To carry out MD simulations with the force field, assembly of the complex involves several steps.

- First, prepin files have to be constructed based on the partial charge and structure data discussed in the previous Sections. This requires the definition of unique structure names that can be grafted onto the amino acid sequence in the PDB files.
- Second, a frcmod file (force constants) is needed to describe the bond, angle and dihedral angle parameters and these must have name for the atoms that are unique and recognizable.
- Third, bonds must be assigned between the ligands and the [2Fe-2S] structure and also the non-heme Fe^2+^.

These files (Listings 5 and 6 in S1 File) should be selected based upon whether the structure to be modeled will be the oxidized state (S1 File, Listing 5) or the reduced state (S1 File, Listing 6). The ligands associated with the Rieske and ROC structure must be renamed in the PDB file to the respective residue names in the corresponding ligands in S1 File (Listings 5 or 6). The application tleap is used to form the bonds between the irons in the [2Fe-2S] (FES) and the two His ligands (HR1 and HR2) and the Cys ligand (CYR). Likewise, leap builds bonds between the Fe-OH2 element (FE2), the two His ligands (HI1 and HI2) and the Asp ligand (ASB).

PDB files have to be constructed by relabeling ligands and the heteroatoms corresponding to the ROC (FE2) and the Rieske (FES). Hydrogens should be assigned to the His residue in the PDB files according to the local environment. This was done with an in-house program in our lab. The numbering of the atoms and heteroatoms in the PDB file were redone to satisfy the indices of the individual chains. This was accomplished by simply loading the file with Leap, producing an output structure and finding the renumbered residues. The critical binding points had to be assigned with the bond option in Leap. After all these relabeling/renumbering steps, it was possible to build the coordinate and topology files correctly and to carry out the MD simulations.

An example of a script to assemble the dioxygenase structure 2DE5 is provided in Listing 10 in S1 File. The specific scripts for using tleap to build the complex (run by the script) are shown in Listing 11 (S1 File) along with a listing of the files containing various settings used for minimizing, warming up, equilibrating and doing production runs. These files were used to build production runs on the 2DE5/6/7 complexes.

This script, and the other related scripts for running leap, as well as an example of a completely prepared file of the 2DE5 structure (file 2DE5o_fixed.pdb) are all provided in S4 File as a zipped folder.

## S1 Listing 10: example script to build the 2DE5 complex

#!/bin/tcsh

# This code is also provided in the S4 Zipped folder as 2DE5_qm0S5_bldx.csh

# NOTE also. The script has been set up and tested ASSUMING that the

# user is submitting this as job to a server that uses TORQUE to do

# the queuing

# see

# http://www.adaptivecomputing.com/products/open-source/torque/

# If this is not the case, then variables such as ${PBS_NODEFILE} and

# ${NCPU} (and the related command line statements) must be modified

# accordingly. Otherwise, this script will simply not work!

# setup environmental variables to the particular system

setenv AMBERHOME /home/myroot/software/amber10/

setenv PMEMD_HOME /home/myroot/software/amber10/bin

setenv MPI_HOME /home/myroot/software/mpich_v1

setenv PATH /my/needed/path1:/my/needed/path2

setenv LD_LIBRARY_PATH /my/library/files

## run the terminal based leap program to construct the input file

tleap -f 2DE5_qp0S5.in

## minimize the structure (to remove clashes within the PDB structure)

${MPI_HOME}/bin/mpirun -np $NCPU -machinefile $PBS_NODEFILE ${AMBERHOME}/bin/sander.MPI -O \

-i min.in \

-o min_2DE5_qp0S5.out \

-p 2DE5_qp0S5.top \

-c 2DE5_qp0S5.crd \

-r min_2DE5_qp0S5.restrt \

-x min_2DE5_qp0S5.crd \

-e min_2DE5_qp0S5.en \

-inf min_2DE5_qp0S5.info

## make a new input PDB file from the minimization

${AMBERHOME}/bin/ambpdb -aatm -p 2DE5_qp0S5.top < min_2DE5_qp0S5.restrt \

> 2DE5_qp0S5min.pdb

## add water and ions to the structure

${AMBERHOME}/bin/tleap -f 2DE5_qp0S5_wbx.in

## minimize the waterbox structure

${MPI_HOME}/bin/mpirun -np $NCPU -machinefile $PBS_NODEFILE ${AMBERHOME}/bin/sander.MPI -O \

-i min_wbx.in \

-o min_2DE5_qp0S5_wbx.out \

-p 2DE5_qp0S5_wbx.top \

-c 2DE5_qp0S5_wbx.crd \

-r min_2DE5_qp0S5_wbx.restrt \

-x min_2DE5_qp0S5_wbx.crd \

-e min_2DE5_qp0S5_wbx.en \

-inf min_2DE5_qp0S5_wbx.info

## make a new input PDB file from the minimization

${AMBERHOME}/bin/ambpdb -aatm -p 2DE5_qp0S5_wbx.top < min_2DE5_qp0S5_wbx.restrt \

> 2DE5_qp0S5_wbxmin.pdb

# This completes building the 2DE5 complex in water with Na+.

# The next step is to begin MD simulation including warm up, equilibration

# and production run on the structure.

## do preparation run warming the waterbox-protein complex linearly

echo "begin preparation run 2DE5_qp0S5_wbx" > 2DE5_qp0S5_warmup.txt

date >> 2DE5_qp0S5_warmup.txt

${MPI_HOME}/bin/mpirun -np $NCPU -machinefile $PBS_NODEFILE \

${PMEMD_HOME}/pmemd -O \

-i prep_wbx_dT.in \

-o 2DE5_qp0S5_wbxprp.out \

-p 2DE5_qp0S5_wbx.top \

-c min_2DE5_qp0S5_wbx.restrt \

-r 2DE5_qp0S5_wbxprp.restrt \

-x 2DE5_qp0S5_wbxprp.crd \

-e 2DE5_qp0S5_wbxprp.en \

-inf 2DE5_qp0S5_wbxprp.info

## do preparation run warming the waterbox-protein complex linearly

echo "begin preparation run 2DE5_qp0S5_wbxeq" >> 2DE5_qp0S5_wbxeq.txt

date >> 2DE5_qp0S5_wbxeq.txt

${MPI_HOME}/bin/mpirun -np $NCPU -machinefile $PBS_NODEFILE \

${PMEMD_HOME}/pmemd -O \

-i prep_wbx_eq.in \

-o 2DE5_qp0S5_wbxeq.out \

-p 2DE5_qp0S5_wbx.top \

-c 2DE5_qp0S5_wbxprp.restrt \

-r 2DE5_qp0S5_wbxeq.restrt \

-x 2DE5_qp0S5_wbxeq.crd \

-e 2DE5_qp0S5_wbxeq.en \

-inf 2DE5_qp0S5_wbxeq.info

# calculate the equilibrium interactions

echo "begin production run 2DE5_qp0S5_00"

echo "begin production run 2DE5_qp0S5_00" >> 2DE5_qp0S5.txt

date >> 2DE5_qp0S5.txt

${MPI_HOME}/bin/mpirun -np $NCPU -machinefile $PBS_NODEFILE \

${PMEMD_HOME}/pmemd -O \

-i equil_300K.in \

-o 2DE5_qp0S5_00.out \

-p 2DE5_qp0S5_wbx.top \

-c 2DE5_qp0S5_wbxeq.restrt \

-r 2DE5_qp0S5_00.restrt \

-x 2DE5_qp0S5_00.crd \

-e 2DE5_qp0S5_00.en \

-inf 2DE5_qp0S5_00.info

echo "finished runs 2DE5_qp0S5_00 to 2DE5_qp0S5_00 (first production run)" \

>> 2DE5_qp0S5.txt

exit 0

## S1 Listing 11: example script to build the 2DE5 complex

### Build the first complex for 2DE5: balance the charge (add Na+/Cl-)

> cat 2DE5_qp0S5.in

source leaprc.ff99SB

addAtomTypes { { "Sb" "S" "sp3" } { "Nv" "N" "sp3" }{ "Np" "N" "sp3" } }

loadAmberParams oxygenase.frcmod

loadAmberPrep amber_qp0S5.prepin

# load in a properly edited and labeled file of 2DE5

x=loadPDB 2DE5o_setup.pdb

bond x.183.NE2 x.390.FE

bond x.187.NE2 x.390.FE

bond x.333.OD1 x.390.FE

bond x.574.NE2 x.781.FE

bond x.578.NE2 x.781.FE

bond x.724.OD1 x.781.FE

bond x.965.NE2 x.1172.FE

bond x.969.NE2 x.1172.FE

bond x.1115.OD1 x.1172.FE

bond x.69.SG x.391.FE1

bond x.90.SG x.391.FE1

bond x.71.ND1 x.391.FE2

bond x.93.ND1 x.391.FE2

bond x.460.SG x.782.FE1

bond x.481.SG x.782.FE1

bond x.462.ND1 x.782.FE2

bond x.484.ND1 x.782.FE2

bond x.851.SG x.1173.FE1

bond x.872.SG x.1173.FE1

bond x.853.ND1 x.1173.FE2

bond x.875.ND1 x.1173.FE2

bond x.1216.SG x.1279.FE1

bond x.1235.SG x.1279.FE1

bond x.1218.ND1 x.1279.FE2

bond x.1238.ND1 x.1279.FE2

bond x.1323.SG x.1386.FE1

bond x.1342.SG x.1386.FE1

bond x.1325.ND1 x.1386.FE2

bond x.1345.ND1 x.1386.FE2

bond x.1429.SG x.1491.FE1

bond x.1448.SG x.1491.FE1

bond x.1431.ND1 x.1491.FE2

bond x.1451.ND1 x.1491.FE2

addIons x Na+ 0

savePDB x 2DE5_qp1S5a.pdb

saveAmberParm x 2DE5_qp1S5a.top 2DE5_qp1S5a.crd

quit

### Build the first complex for 2DE5: add water

> cat 2DE5_qp0S5_wbx.in

source leaprc.ff99SB

addAtomTypes { { "Sb" "S" "sp3" } { "Nv" "N" "sp3" }{ "Np" "N" "sp3" } }

loadAmberParams oxygenase.frcmod

loadAmberPrep amber_qp0S5.prepin

x=loadPDB 2DE5_qp0S5min.pdb

bond x.183.NE2 x.390.FE

bond x.187.NE2 x.390.FE

bond x.333.OD1 x.390.FE

bond x.574.NE2 x.781.FE

bond x.578.NE2 x.781.FE

bond x.724.OD1 x.781.FE

bond x.965.NE2 x.1172.FE

bond x.969.NE2 x.1172.FE

bond x.1115.OD1 x.1172.FE

bond x.69.SG x.391.FE1

bond x.90.SG x.391.FE1

bond x.71.ND1 x.391.FE2

bond x.93.ND1 x.391.FE2

bond x.460.SG x.782.FE1

bond x.481.SG x.782.FE1

bond x.462.ND1 x.782.FE2

bond x.484.ND1 x.782.FE2

bond x.851.SG x.1173.FE1

bond x.872.SG x.1173.FE1

bond x.853.ND1 x.1173.FE2

bond x.875.ND1 x.1173.FE2

bond x.1216.SG x.1279.FE1

bond x.1235.SG x.1279.FE1

bond x.1218.ND1 x.1279.FE2

bond x.1238.ND1 x.1279.FE2

bond x.1323.SG x.1386.FE1

bond x.1342.SG x.1386.FE1

bond x.1325.ND1 x.1386.FE2

bond x.1345.ND1 x.1386.FE2

bond x.1429.SG x.1491.FE1

bond x.1448.SG x.1491.FE1

bond x.1431.ND1 x.1491.FE2

bond x.1451.ND1 x.1491.FE2

solvateBox x TIP3PBOX 10.0

charge x

savePDB x 2DE5_qp0S5_wbx.pdb

saveAmberParm x 2DE5_qp0S5_wbx.top 2DE5_qp0S5_wbx.crd

quit

### Amber files input files

First minimization with the complex and stabilizing ions:

> cat min.in

simple energy minimization of a complex without water and pbc

&cntrl

imin=1, nmropt=0,

ntx=1, irest=0, ntrx=1,

ntxo=1, ntpr=20, ntwr=2000, ntwx=0, ioutfm=0,

ntf=1, ntb=0, cut=12.0,

igb=0,

ntr=0,

maxcyc=20000, ncyc=1000, ntmin=2,

nstlim=2000, nscm=0, t=0.0, dt=0.0005,

temp0=300.0, tempi=0.0, ntt=0, tautp=1.0,

ntp=0, pres0=1.0, taup=1.0,

ntc=1, tol=1.0e-6,

ivcap=0, fcap=1.5,

/

Second minimization with the complex in a waterbox:

> cat min_wbx.in

energy minimization of [complex]_wbx

&cntrl

imin=1, nmropt=0,

ntx=1, irest=0, ntrx=1,

ntxo=1, ntpr=20, ntwr=2000, ntwx=0, ioutfm=0,

ntf=1, ntb=1, cut=8.0,

igb=0,

ntr=0,

maxcyc=2000, ncyc=1000, ntmin=2,

nstlim=2000, nscm=0, t=0.0, dt=0.0005,

temp0=300.0, tempi=0.0, ntt=0, tautp=1.0,

ntp=0, pres0=1.0, taup=1.0,

ntc=1, tol=1.0e-6,

ivcap=0, fcap=1.5,

/

&ewald

eedmeth=1,

/

Warm up the complex and waterbox to the desired temperature:

> cat prep_wbx_dT.in

warm up preparation of complex_wbox from 0 to 300 K using variable tempi

&cntrl

imin=0, nmropt=1,

ntx=1, irest=0, ntrx=1,

ntxo=1, ntpr=50, ntwr=100, iwrap=1, ntwx=5000, ioutfm=0,

ntf=2, ntb=2, cut=8.0,

igb=0,

ntr=0,

nstlim=600000, nscm=500, t=0.0, dt=0.002,

tempi=0.0, ntt=3, vlimit=20, tautp=1.0, gamma_ln=2.,

ntp=1, pres0=1.0, taup=1.0,

ntc=2, tol=1.0e-6,

/

&ewald

eedmeth=1,

/

# temperature regulation

&wt type='TEMP0', istep1=0, istep2=600000,

value1=0.0, value2=300., /

# bath regulation

&wt type='END' /

Equilibrate the complex and waterbox at the desired temperature:

> cat prep_wbx_eq.in

warm up preparation of complex_wbox from 0 to 300 K using variable tempi

&cntrl

imin=0, nmropt=0,

ntx=5, irest=1, ntrx=1,

ntxo=1, ntpr=50, ntwr=50, iwrap=1, ntwx=5000, ioutfm=0,

ntf=2, ntb=2, cut=8.0,

igb=0,

ntr=0,

nstlim=600000, nscm=500, t=0.0, dt=0.002,

tempi=300., temp0=300., ntt=3, vlimit=20, tautp=1.0, gamma_ln=2.,

ntp=1, pres0=1.0, taup=1.0,

ntc=2, tol=1.0e-6,

/

&ewald

eedmeth=1,

/

# temperature regulation

# must use nmropt=1 to have effect

&wt type='TEMP0', istep1=0, istep2=600000,

value1=0., value2=300., /

# bath regulation

# for Andersen thermostate, adjust every 4 ps

&wt type='END' /

Part of a production run:

equilibrium run using constant temperature scaling ntt=1

&cntrl

imin=0, nmropt=0,

ntx=5, irest=1, ntrx=1,

ntxo=1, ntpr=50, ntwr=500, iwrap=1, ntwx=2500, ioutfm=0,

ntf=2, ntb=2, cut=8.0,

igb=0,

ntr=0,

nstlim=500000, nscm=500, t=0.0, dt=0.002,

tempi=300., temp0=300., ntt=3, vlimit=20, tautp=1.0, gamma_ln=2.,

ntp=1, pres0=1.0, taup=1.0,

ntc=2, tol=1.0e-6,

/

&ewald

eedmeth=1,

/

# References

1. Jortner J (1976) Temperature-dependent activation-energy for electron-transfer between biological molecules. J Chem Phys 64: 4860-4867.

2. Bixon M, Jortner J (1997) Electron transfer via bridges. J Chem Phys 107: 5154-5170.

3. Johnson MD, Miller JR, Green NS, Closs GL (1989) Distance dependence of intramolecular hole and electron-transfer in organic radical ions. J Phys Chem 93: 1173-1176.

4. Hammes-Schiffer S, Stuchebrukhov AA (2010) Theory of coupled electron and proton transfer reactions. Chem Rev 110: 6939-6960.

5. Cukier RI, Nocera DG (1998) Proton-coupled electron transfer. Annu Rev Phys Chem 49: 337-369.

6. Layfield JP, Hammes-Schiffer S (2014) Hydrogen tunneling in enzymes and biomimetic models. Chem Rev 114: 3466-3494.

7. Bonin J, Costentin C, Robert M, Saveant JM, Tard C (2012) Hydrogen-bond relays in concerted proton-electron transfers. Acc Chem Res 45: 372-381.

8. Costentin C, Robert M, Saveant JM, Tard C (2010) Inserting a hydrogen-bond relay between proton exchanging sites in proton-coupled electron transfers. Angew Chem Int Ed Engl 49: 3803-3806.

9. Solomon EI, Brunold TC, Davis MI, Kemsley JN, Lee SK, et al. (2000) Geometric and electronic structure/function correlations in non-heme iron enzymes. Chem Rev 100: 235-350.

10. Straganz GD, Nidetzky B (2006) Variations of the 2-His-1-carboxylate theme in mononudear non-heme Fe-III oxygenases. Chembiochem 7: 1536-1548.

11. Nolan LC, O'Connor KE (2008) Dioxygenase- and monooxygenase-catalysed synthesis of cis-dihydrodiols, catechols, epoxides and other oxygenated products. Biotechnol Lett 30: 1879-1891.

12. Dokmanic I, Sikic M, Tomic S (2008) Metals in proteins: correlation between the metal-ion type, coordination number and the amino-acid residues involved in the coordination. Acta Crystallogr D Biol Crystallogr 64: 257-263.

13. Kovaleva EG, Lipscomb JD (2008) Versatility of biological non-heme Fe(II) centers in oxygen activation reactions. Nat Chem Biol 4: 186-193.

14. Pavel EG, Martins LJ, Ellis WR, Solomon EI (1994) Magnetic circular dichroism studies of exogenous ligand and substrate binding to the non-heme ferrous active site in phthalate dioxygenase. Chemistry & Biology 1: 173-183.

15. Solomon EI, Pavel EG, Loeb KE, Campochiaro C (1995) Magnetic circular dichroism spectroscopy as a probe of the geometric and electronic struture of non-heme ferrous enzymes. Coordin Chem Rev 144: 369-460.

16. Ohta T, Chakrabarty S, Lipscomb JD, Solomon EI (2008) Near-IR MCD of the nonheme ferrous active site in naphthalene 1,2-dioxygenase: correlation to crystallography and structural insight into the mechanism of Rieske dioxygenases. J Am Chem Soc 130: 1601-1610.

17. Lipscomb JD (2008) Mechanism of extradiol aromatic ring-cleaving dioxygenases. Curr Opin Struct Biol 18: 644-649.

18. Lipscomb JD, Hoffman BM (2005) Allosteric control of O_2_ reactivity in Rieske oxygenases. Structure 13: 684-685.

19. Bassan A, Blomberg MR, Siegbahn PE (2004) A theoretical study of the cis-dihydroxylation mechanism in naphthalene 1,2-dioxygenase. J Biol Inorg Chem 9: 439-452.

20. Harpel MR, Lipscomb JD (1990) Gentisate 1,2-dioxygenase from *Pseudomonas*. Substrate coordination to active site Fe^2+^ and mechanism of turnover. J Biol Chem 265: 22187-22196.

21. Mason JR, Cammack R (1992) The electron-transport proteins of hydroxylating bacterial dioxygenases. Annu Rev Microbiol 46: 277-305.

22. Chakrabarty S, Austin RN, Deng D, Groves JT, Lipscomb JD (2007) Radical intermediates in monooxygenase reactions of rieske dioxygenases. J Am Chem Soc 129: 3514-3515.

23. Tarasev M, Ballou DP (2005) Chemistry of the catalytic conversion of phthalate into its cis-dihydrodiol during the reaction of oxygen with the reduced form of phthalate dioxygenase. Biochemistry 44: 6197-6207.

24. Bacelo DE, Binning RC, Jr. (2009) DFT comparison of Fe^2+^ hydration in the binding sites of the ferroxidase center of bullfrog M ferritin. J Phys Chem A 113: 1189-1198.

25. Chen K, Que L, Jr. (2001) Stereospecific alkane hydroxylation by non-heme iron catalysts: mechanistic evidence for an Fe(V)=O active species. J Am Chem Soc 123: 6327-6337.

26. Neese F (2008) ORCA: an *ab initio*, density functional and semiempirical program package. In: Neese F, editor. 2.6, 2.7 and 2.8 ed. Bonn (Germany): Frank Neese theochem@thch.uni-bonn.de. pp. A manual for using the ORCA software package, <http://www.thch.uni-bonn.de/tc/orca/index.php>.

27. Frisch MJ, Trucks GW, Schlegel HB, Scuseria GE, Robb MA, et al. (2009) Gaussian 09. Gaussian 09, Revision A.1 ed. Wallingford CT, : Gaussian, Inc.

28. Frisch MJ, Trucks GW, Schlegel HB, Scuseria GE, Robb MA, et al. (2004) Gaussian 03. Gaussian 03, Revision D.02 ed. Wallingford CT, : Gaussian, Inc.

29. Szilagyi RK, Winslow MA (2006) On the accuracy of density functional theory for iron-sulfur clusters. J Comput Chem 27: 1385-1397.

30. Wachers AJH (1970) Gaussian basis set molecular wavefunctions containing third-row atoms. J Chem Phys 52: 1033-1036.

31. Hay PJ (1977) Gaussian basis sets for molecular calculations. The representation of 3d orbitals in transition-metal atoms. J Chem Phys 66: 4377-4384.

32. Raghavachari K, Trucks GW (1989) Highly correleated systems. Excitation energies of first row transition metals Sc-Cu. J Chem Phys 91: 1062-1065.

33. Guell M, Luis JM, Sola M, Swart M (2008) Importance of the basis set for the spin-state energetics of iron complexes. J Phys Chem A 112: 6384-6391.

34. Swart M, Sola M, Bickelhaupt FM (2009) A new all-round density functional based on spin states and S_N_2 barriers. J Chem Phys 131: 094103.

35. Chirlian LE, Francl MM (1987) Atomic charges derived from electrostatic potentials: a detailed study. J Comp Chem 8: 894-905.

36. Breneman CM, Wiberg KB (1990) Determining atom-centered monopoles from molecular electrostatic potentials. The need for high sampling density in formamide conformational analysis. J Comp Biol 11: 361-373.

37. Schaftenaar G, Noordik JH (2000) Molden: a pre- and post-processing program for molecular and electronic structure. J Comput-Aided Mol Design 14: 123-134.

38. Schafer A, Horn H, Ahlrichs R (1992) Fully optimized contracted Gaussian basis sets for atoms Li to Kr. J Chem Phys 97: 2571-2577.

39. Schafer A, Huber C, Ahlrichs R (1994) Fully optimized contracted Gaussian basis sets of triple zeta valence quality for atoms Li to Kr. J Chem Phys 100: 5829-5835.

40. Becke AD (1993) Density-functional thermochemistry .3. The role of exact exchange. J Chem Phys 98: 5648-5652.

41. Noodleman L, Case DA (1999) Density-functional theory of spin polarization and spin coupling in iron-sulfur clusters; Sykes AG, Cammack R, editors. New York: Academic Press, Inc.

42. Coucouvanis D, Salifoglou A, Kanatzidis MG, Simopoulos A, Papaefthymiou V (1984) Dimeric complexes containing the [Fe_2_S_2_]^2+^ cores coordinated by non-sulfur containing terminal ligands. The crystal and molecular structures of the Et_4_N^+^ salts of the [Fe_2_S_2_(o,o'-C_12_H_8_O_2_)_2_]^2-^ and [Fe_2_S_2_(CrH_4_N)_4_]^2-^ anions. J Am Chem Soc 106: 6081-6082.

43. Noodleman L, Peng CY, Case DA, Mouesca J-M (1995) Orbital interactions, electron delocalization and spin coupling in iron-sulfur clusters. Coordin Chem Rev 144: 199-244.

44. Scalmani G, Frisch MJ (2010) Continuous surface charge polarizable continuum models of solvation. I. General formalism. J Chem Phys 132: 114110.

45. Barroso J (2009) Polarizable Continuum Model (PCM) in G03. In: Barroso J, editor. pp. <http://joaquinbarroso.wordpress.com/2009/2009/2007/pcmg2003>

46. Weiner SJ, Kollman PA, Nguyen DT, Case DA (1986) An all atom force-field for simulations of proteins and nucleic-acids. J Comp Chem 7: 230-252.

47. Weiner SJ, Kollman PA, Case DA, Singh UC, Ghio C, et al. (1984) A new force-field for molecular mechanical simulation of nucleic-acids and proteins. J Am Chem Soc 106: 765-784.

48. Shakya SK, Gu W, Helms V (2005) Molecular dynamics simulation of truncated bovine adrenodoxin. Biopolymers 78: 9-20.

49. Cornell WD, Cieplak P, Bayly CI, Kollman PA (1993) Application of RESP charges to calculate conformational energies, hydrogen-bond energies, and free-energies of solvation. J Am Chem Soc 115: 9620-9631.

50. Cieplak P, Cornell WD, Bayly C, Kollman PA (1995) Application of the multimolecule and multiconformational RESP methodology to biopolymers - charge derivation for DNA, RNA, and proteins. J Comp Chem 16: 1357-1377.

51. Wang J, Wolf RM, Caldwell JW, Kollman PA, Case DA (2004) Development and testing of a general AMBER force field. J Comput Chem 25: 1157-1174.

52. Bayly CI, Cieplak P, Cornell WD, Kollman PA (1993) A well-behaved electrostatic potential based method using charge restraints for deriving atomic charges - the RESP model. J Phys Chem 97: 10269-10280.

53. Reed AE, Curtiss LA, Weinhold F (1988) Intermolecular interactions from a natural bond orbital, donor-acceptor viewpoint. Chem Rev 1988: 899-926.

54. Rotsaert FJ, Pikus JD, Fox BG, Markley JL, Sanders-Loehr J (2003) N-isotope effects on the Raman spectra of Fe_2_S_2_ ferredoxin and Rieske ferredoxin: evidence for structural rigidity of metal sites. J Biol Inorg Chem 8: 318-326.

55. Iwasaki T, Kounosu A, Kolling DR, Lhee S, Crofts AR, et al. (2006) Resonance Raman characterization of archaeal and bacterial Rieske protein variants with modified hydrogen bond network around the [2Fe-2S] center. Protein Sci 15: 2019-2024.

56. Tirrell TF, Paddock ML, Conlan AR, Smoll EJ, Jr., Nechushtai R, et al. (2009) Resonance Raman studies of the (His)(Cys)_3_ 2Fe-2S cluster of MitoNEET: comparison to the (Cys)_4_ mutant and implications of the effects of pH on the labile metal center. Biochemistry 48: 4747-4752.

57. Vittal KY, Hare J, Gewirth A, Czernuszewicz RS, Kimura T, et al. (1983) Resonance Raman spectra of spinach ferredoxin and adrenodoxin and of analogue complexes. J Am Chem Soc 105: 6462-6468.

58. Fu W, Drozdzewski PM, Davies MD, Sligar SG, Johnson MK (1992) Resonance Raman and magnetic circular dichroism studies of reduced [2Fe-2S] proteins. J Biol Chem 267: 15502-15510.

59. Ozaki Y, Nagayama K, Kyogoku Y, Hase T, Matsubara H (1983) Resonance Raman-spectroscopic study on the iron sulfur proteins containing [2Fe-2S] clusters. Febs Lett 152: 236-240.

60. Andersson KK, Cox DD, Que L, Jr., Flatmark T, Haavik J (1988) Resonance Raman studies on the blue-green-colored bovine adrenal tyrosine 3-monooxygenase (tyrosine hydroxylase). Evidence that the feedback inhibitors adrenaline and noradrenaline are coordinated to iron. J Biol Chem 263: 18621-18626.

61. Oehrstroem L, Machaud-Soret I (1996) Quantum chemical approach to the assignment of iron-catecholate vibrations and isotopic substitution shifts. J Am Chem Soc 118: 3283.

62. Proshlyakov DA, Henshaw TF, Monterosso GR, Ryle MJ, Hausinger RP (2004) Direct detection of oxygen intermediates in the non-heme Fe enzyme taurine/alpha-ketoglutarate dioxygenase. J Am Chem Soc 126: 1022-1023.

63. Pyrz JW, Roe AL, Stern LJ, Que L (1985) Model studies of iron tyrosinate proteins. J Am Chem Soc 107: 614-620.

64. Dave BC, Czernuszewicz RS, Prickril BC, Kurtz DM, Jr. (1994) Resonance Raman spectroscopic evidence for the FeS_4_ and Fe-O-Fe sites in rubrerythrin from Desulfovibrio vulgaris. Biochemistry 33: 3572-3576.
